# Targeting N-acetyltransferase 10 reprograms Treg function to enhance anti–PD-1 immunotherapy sensitivity in murine lung tumors

**DOI:** 10.1172/JCI189034

**Published:** 2026-11-02

**Authors:** Xuewei Qi, Dan Cui, Xiao Li, Wenfan Fu, Handong Ma, Wenting Guo, Jiabao Wang, Jiaxi Song, Jiaxin Ma, Jing Yang, Haoyu Yu, Yuting Hong, Qian Wang, Susu Luo, Jia Wang, Baixing Wu, Shanshan Li, Hao Cheng, Min Cheng, Bin Li, Kaiming Zhang, Jia Li, Fengyin Li

**Affiliations:** 1 State Key Laboratory of Immune Response and Immunotherapy, School of Basic Medical Sciences, Division of Life Sciences and Medicine, University of Science and Technology of China, Hefei, China.; 2 Guangzhou National Laboratory, Xing Dao Huan Bei Road, Guangzhou International Bio Island, Guangzhou, China.; 3 Ministry of Education Key Laboratory for Cellular Dynamics, Center for Advanced Interdisciplinary Science and Biomedicine of Institute of Health and Medicine (IHM), Division of Life Sciences and Medicine, University of Science and Technology of China, Hefei, China.; 4 Department of Chest Surgery, Affiliated Cancer Hospital and Institute of Guangzhou Medical University, Guangzhou, China.; 5 Guangdong Provincial Key Laboratory of Malignant Tumor Epigenetics and Gene Regulation, Guangdong-Hong Kong Joint Laboratory for RNA Medicine, Medical Research Center, Sun Yat-Sen Memorial Hospital, Sun Yat-Sen University, Guangzhou, China.; 6 Affiliated Cancer Hospital and Institute of Guangzhou Medical University, Guangzhou, China.; 7 Guangzhou Medical University–Guangzhou Institutes of Biomedicine and Health Joint School of Life Sciences, Guangdong-Hong Kong-Macau Joint Laboratory for Cell Fate Regulation and Diseases, Guangzhou Medical University, Guangzhou, China.; 8 Center for Immune-related Disease at Shanghai Institute of Immunology, Department of Respiratory and Critical Care Medicine of Ruijin Hospital, Shanghai Jiao Tong University School of Medicine, Shanghai, China.; 9 Department of Geriatrics, Gerontology Institute of Anhui Province, The First Affiliated Hospital of University of Science and Technology of China, Division of Life Sciences and Medicine, University of Science and Technology of China, Hefei, China.; 10 State Key Laboratory of Respiratory Disease, National Clinical Research Center for Respiratory Disease, Guangzhou Institute of Respiratory Health, The First Affiliated Hospital of Guangzhou Medical University, Guangzhou, China.; 11 Center for Advanced Interdisciplinary Science and Biomedicine of IHM, Division of Life Sciences and Medicine, University of Science and Technology of China, Hefei, China.

**Keywords:** Cell biology, Oncology, Adaptive immunity

## Abstract

A significant challenge for improving the efficacy of immune checkpoint blockade (ICB) therapy is the concurrent activation of Tregs, which can dampen antitumor responses. Thus, elucidating the mechanisms that maintain Treg-suppressive function within the tumor microenvironment is critical. Here, we identified an accumulation of N-acetyltransferase 10–modified (NAT10-modified) RNA, particularly in NAT10^+^FOXP3^+^ Tregs, in human non–small cell lung cancer. Genetic ablation of *Nat10* in tumor-infiltrating (TI) Tregs, or pharmacological inhibition of NAT10 expression, significantly reduced the PD-1^+^ Treg/CD8^+^ T cell ratio. These effects were accompanied by an increase in IFN-γ^+^ Tregs, a reduction in the immunosuppressive activity of Tregs, and inhibition of tumor progression. Mechanistically, NAT10 promotes TI Treg infiltration and activation independently of its RNA-modifying function by directly interacting with FOXP3 and chromatin, modulating local 3D chromatin contacts. Combined administration of anti–PD-1 antibodies and a NAT10 inhibitor further impaired TI Treg–mediated immunosuppression, leading to enhanced tumor control. Our findings demonstrate that NAT10 is a key regulator of TI Treg fragility and function and that its inhibition may enhance the therapeutic efficacy of ICB.

## Abstract 2

NAT10 reshapes Treg function to boost anti-PD-1 immunotherapy efficacy in mouse lung tumors

## Introduction

Increasing evidence indicates that elevated frequencies of tumor-infiltrating (TI) Tregs correlate with poor prognosis in patients with cancer. Tregs, a subset of CD4^+^ T cells defined by the expression of the transcription factor FOXP3, are crucial for maintaining immune homeostasis and preventing autoimmunity (1–4); however, they also suppress antitumor immunity and hinder cancer immunotherapy (5–7). These TI Tregs are highly suppressive (8), and their accumulation is linked to adverse clinical outcomes in multiple cancer types (9–12). Notably, functionally heterogeneous Treg subpopulations contribute to early lung tumorigenesis, metastasis, and immunotherapy resistance (13–15).

Given these findings, TI Tregs are considered a major barrier to effective immunotherapy. However, systemic Treg depletion carries a high risk of autoimmunity and inflammation, underscoring the need for strategies that selectively target TI Tregs (8). Efforts to inhibit TI Tregs have focused on surface molecules and signaling pathways critical to their stability and function, including NRP1, CXCR3, CBM signalosome, NF-κB, and EZH2, which also synergize with PD-1 blockade (16–20). Therefore, understanding the mechanisms governing TI Treg accumulation and suppressive function is essential for improving immunotherapy outcomes.

FOXP3 is indispensable for Treg identity and suppressive function; however, its expression alone does not ensure full lineage commitment or functional maturation, especially under tumor-associated stress (13). FOXP3 reprograms cellular metabolism by inhibiting Myc and glycolysis while promoting oxidative phosphorylation and NAD^+^ oxidation, enabling Treg survival in glucose-deprived, lactate-rich tumor microenvironments (TMEs) (21). The disruption of FOXP3^+^ Tregs and the transcription of *Foxp3* itself, enhanced by Ku70, can impact antitumor immunity (22, 23). This advantage enables cancer cells to evade immune destruction within the TME (21). However, the mechanism by which FOXP3 influences Treg reprogramming in the TME remains largely uncharacterized. Particularly noteworthy is the growing evidence suggesting that anti–PD-1 checkpoint immunotherapy may enhance Treg infiltration and activation, potentially leading to immune-related adverse events (24).

In parallel, RNA epigenetics has emerged as a crucial layer of gene regulation that modulates RNA fate through posttranscriptional modifications (25). Recent studies have shown that RNA modifications influence T cell expansion (26) and are required for TI Treg survival and function (27). Among these, N-acetyltransferase 10 (NAT10), the first characterized enzyme catalyzing N4-acetylcytidine (ac4C) mRNA modifications, has been shown to regulate T cell proliferation and antiviral responses (28) and is involved in cycling T cell–driven autoimmunity in mice (29). However, whether NAT10 plays a role in regulating Tregs in the context of antitumor immunity remains largely unknown.

In this study, we demonstrate that NAT10 supported Treg infiltration and activation within the TME. Using mice with Treg-specific *Nat10* deletion, we showed that *Nat10* deficiency enhanced antitumor immunity by impairing TI Treg–suppressive function and improved the efficacy of PD-1 blockade. Mechanistically, we revealed that (a) NAT10 interacted with FOXP3 and chromatin to regulate *Cxcr4* expression and promote Treg tumor homing; (b) FOXP3 required NAT10’s chromatin-binding function to activate the Treg gene program; and (c) anti–PD-1 and chemotherapy induced MHC-II expression in tumor cells and enhanced *Nat10* mRNA levels in TI Tregs, promoting costimulatory and TNF receptor (TNFR) superfamily molecule expression for facilitating TI Treg activation and functional maturation. Together, our findings establish NAT10 as a key modulator of TI Treg function and a promising target for improving cancer immunotherapy.

## Results

### NAT10 is essential for the activation of intratumoral Tregs.

To investigate the role of RNA epigenetic modifications in intratumoral Tregs, we analyzed single-cell RNA-seq (scRNA-seq) data from patients with non–small cell lung cancer (NSCLC) (GSE99254), identifying 11,769 TI lymphocytes (TILs). From this original dataset, we first identified 5,547 CD4^+^ T cells and 2,612 Tregs (Supplemental Figure 1, A and B; supplemental material available online with this article; https://doi.org/10.1172/JCI189034DS1). Given the enrichment of CD4^+^FOXP3^+^CTLA4^+^ Tregs in tumors (30), we further focused on this subset (1,146 cells) and computed Treg activation scores based on canonical markers (*LAG3*, *PDCD1*, *CXCR3*, *TNFRSF18*, *TNFRSF4*, *TNFRSF9*, *CCR8*, *CTLA4*, *ICOS*, *ENTPD1*, *LAYN*, *IL2RA*, and *FOXP3*). Clusters 1 and 3 showed markedly higher activation scores than clusters 0 and 2 (Figure 1, A and B). Differential expression analysis between these groups revealed distinct gene expression patterns (Figure 1C). To identify regulators of Treg activation, we calculated Pearson correlation coefficients between Treg activation scores and the expression of RNA epigenetic modifiers. Genes that were both highly expressed in clusters 1 and 3 and showed strong positive correlations were considered potential activators of these clusters. Among these, *NAT10* was most significantly upregulated (FDR = 0.01) and displayed the strongest correlation with Treg activation (*r* = 0.33) (Figure 1D), outperforming other modifiers such as *METTL3*, *METTL4*, *METTL6*, *METTL8*, and *METTL14* (Supplemental Table 1).

These findings were corroborated using Tregs from treatment-naive patients with lung cancer (GSE235500) (Supplemental Figure 1, C–H). Immunofluorescence staining confirmed the presence of NAT10^+^CD4^+^FOXP3^+^ Tregs infiltrating NSCLC tumor tissues (Figure 1E, Supplemental Figure 2A, Supplemental Figure 3, and Supplemental Table 2), with some tumor cells also expressing NAT10, consistent with previous reports (31). Quantitative immunofluorescence analysis revealed that, on average, 20% of NAT10^+^ cells were CD4^+^FOXP3^+^ Tregs, while 45% of CD4^+^FOXP3^+^ Tregs were NAT10^+^ (Figure 1F).

To evaluate the clinical relevance of *NAT10* expression, we analyzed transcriptomic data from 439 patients with NSCLC treated with atezolizumab (anti–PD-L1) (32, 33). Focusing on samples with *FOXP3* ≥2 transcripts per million (TPM) (*n* = 269), we calculated the *NAT10/FOXP3* ratio and stratified patients into high and low groups based on the median value (*n* = 134 vs. 135) (34). Kaplan-Meier and Cox regression analyses revealed that a higher *NAT10/FOXP3* ratio was significantly associated with poorer survival (*P* = 0.0029) (Figure 1G). A similar study in the paclitaxel-treated chemotherapy cohort (*n* = 372) showed a marginal association (*P* = 0.052) (Figure 1H). Multivariate analysis with available data for the *NAT10/FOXP3* TPM ratio, PD-L1 expression, immune infiltration score, and histological subtype showed that the *NAT10/FOXP3* TPM ratio remained significantly associated with survival in atezolizumab-treated (*n* = 269, *P* = 0.049) and chemotherapy (*n* = 61, *P* = 0.021) patients with NSCLC. Detailed patient information is provided in the Supplemental Data Set 1 (Excel for multivariant survival). Together, these results suggest a correlation between elevated *NAT10* signal in *FOXP3*^+^‑rich tumor regions and poorer clinical outcomes. The bulk‑derived *NAT10/FOXP3* TPM ratio exhibits an independent statistical association with survival among patients with NSCLC treated with anti–PD-L1 or chemotherapy.

In mice lung tumor model, immunofluorescence analysis confirmed the presence of NAT10^+^ FOXP3^+^ Tregs (Supplemental Figure 2B). Given that TI Tregs may arise from CD4^+^ T cells activated by antigen presentation via MHC molecules on dendritic or tumor cells, we examined whether *Nat10* expression in TI Tregs, or MHC molecule expression in antigen-presenting cells, was induced during anti–PD-1, cisplatin, or paclitaxel therapy (Supplemental Figure 4A). These treatments markedly upregulated *Nat10* expression in TI Tregs (Figure 1I). Additionally, anti–PD-1 and paclitaxel treatments upregulated MHC-II (*mH2-A* and *mH2-E*) and MHC-I (*mH2-D* and *mH2-K*) expression in tumor cells (Supplemental Figure 4, B and C) but not in dendritic cells (Supplemental Figure 4, D and E). These data suggest that drug therapy–induced MHC-II expression on tumor cells may facilitate CD4^+^ T cell activation and promote iTreg (iTreg) differentiation in the TME.

### Nat10 ablation in TI Tregs suppresses tumor progression.

To assess the requirement of NAT10 in Tregs, we designed single guide RNAs (sgRNAs) targeting exons 4–5, which are critical for protein function (35), and generated *Nat10*^flox^ mice via CRISPR. LoxP sites were confirmed by genomic sequencing (Supplemental Figure 5A). To investigate whether *Nat10* ablation destabilizes intratumoral Tregs, we generated *Foxp3*^GFP-cre-ERT2^ × *Nat10*^fl/fl^ mice in which tamoxifen administration triggers FOXP3^+^ Treg-specific deletion of *Nat10* and simultaneous GFP expression. As expected, GFP was observed exclusively in CD4^+^FOXP3^+^ Tregs from both the spleen and TILs of B16F10-bearing mice but not in CD4^+^FOXP3^–^ non-Tregs or CD8^+^ T cells (Supplemental Figure 5, B and C).

Subsequent evaluation revealed that NAT10 expression was diminished in Tregs from tamoxifen-treated *Nat10*^fl/fl^ *Foxp3*^GFP-cre-ERT2^ mice (Figure 2, A and B). These mice exhibited markedly delayed tumor growth and prolonged survival compared with *Nat10*^+/+^ *Foxp3*^GFP-cre-ERT2^ controls (Figure 2, C–G, and Supplemental Figure 5, D–I). Flow cytometry analysis of TI Tregs showed a significant reduction in both frequency and number in *Nat10*-cKO mice relative to controls, regardless of tumor models (Figure 2H and Supplemental Figure 6, A and C), whereas FOXP3 protein levels remained unchanged (Figure 2I and Supplemental Figure 6B). In contrast, the frequency and number of Tregs in the spleen were comparable between *Nat10*-Ctrl and *Nat10*-cKO mice (Supplemental Figure 7, A and B), suggesting that *Nat10* deletion selectively affects intratumoral but not systemic Treg populations. These findings indicate that *Nat10* ablation may restrict Treg infiltration or iTreg differentiation specifically within the TME.

In both lung and subcutaneous tumor models, *Nat10*-cKO mice exhibited significantly reduced ratios of TI Tregs to CD8^+^ T cells and TI PD-1^+^ Tregs to PD-1^+^ CD8^+^ T cells compared with controls (Supplemental Figure 7, C and D). Frequency and expression of key activation and immunosuppressive markers, including PD-1, CD25, CXCR3, KLRG1, CTLA4, NRP1, and ICOS, were markedly downregulated in TI Tregs from *Nat10*-cKO mice (Figure 2, J and K, Supplemental Figure 6, D–J, and Supplemental Figure 7, E–I). Notably, although the number of TI CD8^+^ T cells remained unchanged, their frequency significantly increased in the *Nat10*-cKO group (Supplemental Figure 8A). Both the frequency and number of CD44^hi^CD62L^–^ effector CD8^+^ T cells were significantly higher in *Nat10*-cKO mice (Supplemental Figure 8B), along with a marked elevation in CD44 protein levels (Supplemental Figure 8C). Together, these results demonstrate that conditional *Nat10* deletion in Tregs reduces their accumulation and suppressive phenotype within tumors, excluding FOXP3 expression, while enhancing the activation of cytotoxic CD8^+^ T cells. This suggests that NAT10 plays a key role in maintaining intratumoral Treg function and phenotype.

### Nat10 ablation in TI Tregs enhances antitumor immunity.

IFN-γ is known to induce intratumoral Treg functional fragility, impairing their suppressive capacity without disrupting FOXP3 expression (36). Similarly, deletion of *Nrp1*, *Pdcd1*, or *Jmjd1c* enhances IFN-γ production and promotes Treg fragility, thereby improving antitumor immunity (20, 37, 38). In our experiments, TI Tregs from *Nat10*-cKO mice secreted significantly more IFN-γ following PMA/ionomycin (Figure 3A) or anti-CD3/anti-CD28 (Supplemental Figure 8D) stimulation, indicating increased functional fragility. To further investigate whether *Nat10* deficiency influences Treg fragility, we performed RNA-seq on TI Tregs from control and *Nat10*-cKO mice and analyzed the data by using both curated gene set enrichment analysis (39). The MSigDB GSE14308 Th1-versus-natural Treg signatures showed that the GSE14308_T_H_1_VS_NATURAL_Treg_UP gene set was enriched in *Nat10*-deficient TI Tregs (normalized enrichment score [NES] = 1.92; FDR *q* = 6.69 × 10^–7^) (Supplemental Figure 8E), whereas the reciprocal GSE14308_T_H_1_VS_NATURAL_Treg_Down gene set was enriched in control TI Tregs (NES = –1.68; FDR *q* = 4.75 × 10^–5^) (Supplemental Figure 8F). Together, these data indicate that *Nat10*-deficient TI Tregs display a Th1-skewed transcriptional profile with attenuation of multiple Treg-associated genes, consistent with increased Treg fragility.

To further examine the suppressive capacity of *Nat10*-deficient Tregs, we performed both in vivo and in vitro assays. In vivo, *Rag1*^−/−^ mice were adoptively transferred with CD45.1^+^ naive CD4^+^ T cells alone or with WT or *Nat10*-deficient Tregs, followed by tamoxifen. Mice receiving *Nat10*-deficient Tregs were less protected from weight loss compared with those receiving WT Tregs, indicating impaired immunoregulatory function (Figure 3B). In vitro, CellTrace Violet–labeled (CTV-labeled) CD45.1^+^CD8^+^ T cells were cocultured with WT or *Nat10*-deficient TI Tregs for 72 hours. CD8^+^ T cells cocultured with *Nat10*-deficient Tregs proliferated significantly more than those cocultured with WT Tregs (Figure 3C), confirming that loss of *Nat10* weakens Treg-mediated suppression of CD8^+^ T cell activation. Moreover, TI CD8^+^ T cells from mice with *Nat10*-deficient Tregs secreted higher levels of granzyme B, IFN-γ, perforin, and TNF-α (Figure 3, D–G), indicating an enhanced cytotoxic potential. Taken together, these findings demonstrate that *Nat10* deletion increases Treg fragility and diminishes their suppressive function on conventional CD4^+^ and CD8^+^ T cells, thereby enhancing antitumor immunity. This highlights NAT10 as a key regulator of Treg fragility and immunosuppression within the TME.

### NAT10 is essential for the differentiation of iTregs in tumors.

To assess whether NAT10 is intrinsically required for iTreg development, we generated *Nat10*^fl/fl^ *Ox40*^cre^ mice. Since OX40 is expressed in activated but not naive CD4^+^ T cells, this model avoided confounding effects on T cell homeostasis. CTV-labeled naive CD4^+^ T cells were cultured with TGF-β and IL-2 for 96 hours to induce iTreg differentiation (Supplemental Figure 9A). *Nat10*-deficient CD4^+^ T cells showed a significantly reduced frequency of iTregs compared with controls (Supplemental Figure 9B). Both control and *Nat10*-deficient CD4^+^ cells divided up to 6 times (Supplemental Figure 9D) but a greater proportion of *Nat10*-deficient iTregs stalled at the first division (Supplemental Figure 9C). When *Nat10* was deleted in naive CD4^+^ T cells via *Cd4*^cre^, cells failed to divide beyond the first round (Supplemental Figure 9E), suggesting that NAT10 is essential for initiating iTreg differentiation.

To determine whether NAT10 is required for iTreg differentiation in vivo, we conditionally deleted *Nat10* in TI Tregs using tamoxifen in B16F10-bearing mice (Supplemental Figure 10A). After 14 days, the frequency and number of Tregs from *Nat10*-deficient mice were significantly reduced compared with controls (Supplemental Figure 10B), despite unaltered FOXP3 protein expression (Supplemental Figure 10C). Analysis of the Treg-specific demethylated region revealed an approximately 20% reduction in CpG demethylation in *Nat10*-deficient Tregs compared with controls (Supplemental Figure 10D), suggesting compromised epigenetic commitment. To confirm this, we adoptively transferred naive CD4^+^ T cells from *Nat10*^fl/fl^ *Ox40*^cre^ and control mice into CD45.1^+^ recipients bearing B16F10 tumors (Supplemental Figure 10E). After 14 days, the frequency and number of Tregs derived from *Nat10*-deficient donors were markedly reduced compared with controls (Supplemental Figure 10F), although FOXP3 expression was unchanged (Supplemental Figure 10G). These results suggest that NAT10 is potentially required for iTreg differentiation from activated CD4^+^ T cells within the TME.

Indeed, in tumors from *Nat10* cKO mice bearing B16F10, the frequency and number of CD44^hi^CD62L^–^ effector and CD44^+^CD69^+^ activated Tregs were significantly reduced compared with controls (Supplemental Figure 11, A, B, and D). Concomitantly, the frequency of naive-like Tregs were increased, although their numbers remained unchanged (Supplemental Figure 11C). Consistently, *Nat10*-deficient Tregs exhibited markedly lower CD44 and CD69 expression (Supplemental Figure 11, E and F). To evaluate proliferative capacity, Tregs were sorted and labeled with CTV (Supplemental Figure 11G). Flow cytometry data revealed that *Nat10*-deficient Tregs displayed a significantly lower rate of cell division compared with controls (Supplemental Figure 11H). Collectively, these results demonstrate that NAT10 is critical not only for iTreg differentiation but also for maintaining activated and effector Treg populations within the TME.

### NAT10 interacts with FOXP3 to promote iTreg differentiation in the TME.

To explore the mechanisms underlying the impaired Treg upon *Nat10* deletion, we examined the localization of the NAT10 protein. Western blot revealed that NAT10 was predominantly nuclear in Tregs, with no detectable cytoplasmic expression (Supplemental Figure 12A). TI Tregs from lung tumor model were sorted for ac4C RNA immunoprecipitation sequencing (RIP-seq) analysis. The ac4C signal intensity was comparable between *Nat10*-deficient and control TI Tregs (Supplemental Figure 12B). Furthermore, stratifying genes based on high or low ac4C signals showed no marked downregulation in *Nat10*-deficient cells regardless of ac4C enrichment (Supplemental Figure 12C). These results suggest that NAT10’s role in TI Treg differentiation is largely independent of its RNA acetyltransferase activity via ac4C modification.

Next, we performed CUT&Tag profiling on TI Tregs sorted from lung tumor model. Comparative analysis of NAT10 and FOXP3 CUT&Tag datasets revealed that 34.9% of NAT10 peaks overlapped with FOXP3 binding sites (clusters 1 and 2), while 65.1% represented NAT10-only regions lacking FOXP3 enrichment (cluster 3) (Figure 4A). Cobound regions exhibited elevated H3K27ac and assay for transposase-accessible chromatin with high-throughput sequencing (ATAC-seq) signals, along with reduced DNA methylation (Figure 4, B–D, red lines), indicating increased chromatin accessibility and transcriptionally activity. Whereas NAT10-only sites were depleted of activation marks and showed high DNA methylation (Figure 4, B–D, blue lines). Importantly, NAT10-FOXP3 cobound regions were enriched in promoters of Treg signature genes (Figure 4, E and F), whereas NAT10-only binding sites were enriched in conventional CD4^+^ T cell genes (Supplemental Figure 12D). These findings suggest that NAT10 cooperates with FOXP3 to regulate Treg-specific gene expression and iTreg differentiation.

Consistent with prior reports of FOXP3 binding at H3K27ac-enriched regions, we observed cooccupancy of NAT10 and H3K27ac (Supplemental Figure 12, E and F). Correlation analyses demonstrated strong associations between NAT10 and FOXP3 as well as between NAT10 and H3K27ac (Spearman coefficients = 0.52 and 0.76, respectively; Figure 4, G and H), suggesting synergistic regulation of Treg activation in the TME. Protein co-IP confirmed the physical interaction between NAT10 and FOXP3 in TI Tregs (Figure 4, I and J). FOXP3 CUT&Tag analysis revealed a marked loss of peaks within clusters 1 and 2 in *Nat10*-deficient TI Tregs (Figure 4K). In contrast, regions lacking FOXP3 in cluster 3 remained unaffected (Supplemental Figure 12G). H3K27ac CUT&Tag analysis identified 2,898 peaks with reduced H3K27ac enrichment in *Nat10*-deficient Tregs (Figure 4L). These peaks were associated with genes involved in T cell aggregation, activation, differentiation, and immune regulation (Supplemental Figure 12, H and I). In summary, our data demonstrate that NAT10 cooperates with FOXP3 to direct the transcriptional and epigenetic programs essential for iTreg differentiation and function in the TME. Loss of *Nat10* disrupts FOXP3 binding and chromatin activation, thereby impairing TI iTreg generation and maintenance.

### NAT10 facilitates natural Treg recruitment and iTreg activation via direct DNA binding, independent of RNA acetylation.

To further investigate the impaired suppressive capacity of TI Tregs following *Nat10* ablation, we integrated Treg activation and function markers with NAT10 and H3K27ac CUT&Tag data. Our analysis revealed strong enrichment of enhancers and superenhancers at gene loci critical for Treg costimulation and activation in TI Tregs, including *Tnfrsf18* (GITR), *Tnfrsf4* (OX40), *Pdcd1* (PD-1), *Tnfrsf9* (4-1BB), *Icos*, and *Cxcr4* (Figure 5A). Analysis of our 3D genome data showed that NAT10, together with FOXP3, participates in loop formation at loci such as *Cxcr4*, which promotes Treg recruitment to the TME (40). Furthermore, the chromosome loops with anchors cobinding with NAT10 and FOXP3 were impaired in *Nat10*-deficient Tregs (Figure 5B). CUT&Tag and ATAC-seq analyses demonstrated a marked reduction in H3K27ac signal and chromatin accessibility at *Cxcr4* loci in *Nat10*-deficient TI Tregs, accompanied by a marked decrease in FOXP3 binding (Figure 5C). FOXP3 CUT&Tag-qPCR confirmed that FOXP3 enrichment at the *Cxcr4* locus was substantially reduced in *Nat10*-deficient Tregs (Figure 5D). Flow cytometry further demonstrated a significant decrease in CXCR4 protein expression in *Nat10*-deficient TI Tregs (Figure 5E). As CXCR4 is a key homing receptor for immune cells migrating to the bone marrow, a known reservoir for tumor antigen–specific and TI Tregs in patients with cancer (41), we examined CXCR4^+^ Treg populations in the bone marrow of mice bearing B16F10 lung tumors. The frequency of CXCR4^+^ cells and CXCR4 protein levels were significantly reduced in *Nat10*-deficient Tregs compared with controls (Supplemental Figure 13A). To further assess NAT10’s role in Treg accumulation within tumors, we cotransferred *Nat10*-cKO Tregs (CD45.2^+^) and control Tregs (CD45.1^+^ CD45.2^+^) into tumor-bearing mice (Figure 5F). Flow cytometry data revealed significantly lower frequencies and numbers of CD45.2^+^ *Nat10* cKO Tregs compared with CD45.1^+^CD45.2^+^ control Tregs within tumors (Figure 5G). These data collectively suggest that NAT10 promotes Treg recruitment to the TME, potentially through modulating local chromatin‑loop interactions and establishing Treg-specific superenhancer landscapes.

*Nat10* ablation also markedly reduced H3K27ac enrichment and chromatin accessibility at *Pdcd1*, *Tnfrsf9*, *Tnfrsf4*, *Tnfrsf18*, and *Icos* loci (Figure 5, H and K, and Supplemental Figure 13, B and E). FOXP3 CUT&Tag-qPCR confirmed a marked reduction in FOXP3 occupancy at these loci in *Nat10*-deficient TI Tregs (Figure 5, I and L, and Supplemental Figure 13G). Correspondingly, flow cytometry data revealed reduced protein expression of PD-1, 4-1BB, OX40, GITR, and ICOS in *Nat10*-deficient TI Tregs (Figure 5, J and M, and Supplemental Figure 13, C, D and F), indicating that NAT10 is essential for sustaining the expression of key Treg activation and immunosuppressive markers in the TME.

To examine whether NAT10 exerts a similar role in humans, we performed CUT&Tag on healthy human CD4^+^CD25^–^ T cells and in vitro iTregs (Supplemental Figure 14A). iTregs showed increased enrichment of NAT10, FOXP3, and H3K27ac signals compared with CD4^+^CD25^–^ T cells (Supplemental Figure 14, B–D). Comparative analysis of NAT10 and FOXP3 profiles revealed that genomic regions cooccupied by both factors in iTregs exhibited higher signal intensity following the differentiation of CD4^+^CD25^–^ T cells (Supplemental Figure 14E). Regions cooccupied by NAT10 and FOXP3 exhibited higher signal intensity in iTregs, particularly near genes involved in T cell costimulation and activation, such as *TNFRSF9*, *PDCD1*, *ICOS*, *TNFRSF18*, *TNFRSF4*, and *CXCR4*, which were also identified as *Nat10*-target genes in murine TI Tregs (Supplemental Figure 14, F–J). These results suggest that NAT10 and FOXP3 may play conserved roles in shaping chromatin accessibility at activation- and immunosuppression-associated loci during human iTreg differentiation.

To further determine whether NAT10 was required for the expression of these activation- and immunosuppression-associated markers during CD4^+^ T cell activation, we utilized *Ox40*^cre^ mice to delete *Nat10* in activated CD4^+^ T cells. Naive CD4^+^ T cells from *Nat10*^+/+^ *Ox40*^cre^ and *Nat10*^fl/fl^ *Ox40*^cre^ mice were sorted and activated in vitro (Supplemental Figure 15A). Surprisingly, *Nat10*-deficient activated CD4^+^ T cells expressed comparable levels of PD-1, 4-1BB, ICOS, OX40, GITR, and CXCR4 to controls (Supplemental Figure 15, B–H), indicating that NAT10 is dispensable for the expression of these markers in activated CD4^+^ T cells.

### NAT10 was required for activation- and immunosuppression-associated gene expression in iTregs.

To confirm NAT10’s DNA-binding capacity, we first purified the NAT10 protein (Figure 6A) and performed electrophoretic mobility shift assays (EMSAs) with 8 candidate DNA sequences (Supplemental Table 3). Among these, DNA2, DNA5, and DNA8 showed strong binding affinity for NAT10, as evidenced by pronounced band shifts (Supplemental Figure 16, A–H). Sequence alignment of DNA2, DNA5, and DNA8 identified a conserved 46-nucleotide motif (termed DNA9), which was used in binding analyses and confirmed NAT10’s DNA-binding capability (Figure 6B).

Although NAT10 is an RNA acetyltransferase, it lacks canonical DNA-binding motifs such as zinc fingers or helix-turn-helix domains. Instead, it appears to bind DNA via electrostatic interactions mediated by positively charged residues within exons 4–5 (pink region), distinct from the its RNA acetyltransferase catalytic residues H537/Y538 (green region) (42) (Figure 6C). Deletion of exons 4–5 markedly impaired DNA-binding ability (Figure 6D), whereas mutation of H537A/Y538A had no effect (Figure 6, E and F), indicating that NAT10 can directly bind DNA independent of its acetyltransferase activity. To determine whether NAT10 directly regulates downstream target gene expression via its genomic DNA-binding activity, we constructed a luciferase reporter plasmid incorporating the promoter region of a NAT10-bound activation-associated gene, including the 46 bp conserved motif (Supplemental Figure 16I). Cotransfection of this reporter with a *Nat10* overexpression plasmid into HEK293T cells significantly increased luciferase activity compared with the empty vector (Supplemental Figure 16J). These data suggest that NAT10 can directly promote the transcription of activation- and immunosuppression-related genes in iTregs via genomic DNA binding.

To further elucidate the role of NAT10 in iTreg differentiation, we expressed WT *Nat10*, a catalytically inactive mutant (mut*Nat10*_H537A/Y538A_), and a DNA-binding mutant (exon 4–5 deletion, mut*Nat10*_exon4–5_) in differentiating iTregs (Figure 6G). The iTregs from *Nat10*^fl/fl^ *Ox40*^cre^ mice showed significantly reduced expression of PD-1, 4-1BB, ICOS, OX40, GITR, and CXCR4 compared with *Nat10*^+/+^ *Ox40*^cre^ controls. Remarkably, ectopic expression of WT *Nat10* or mut*Nat10*_H537A/Y538A_ restored expression of these proteins, whereas mut*Nat10*_exon4–5_ did not (Figure 6, H–J, and Supplemental Figure 16, L–N). In addition, the purified mutNAT10_H537A/Y538A_ protein showed a marked reduction of its acetyltransferase activity compared with WT NAT10 protein (Supplemental Figure 17, A and B). The molecular size peak chart and electrophoresis results demonstrated that the integrity and stability of mutNAT10_exon4–5_ was similar with mutNAT10_H537A/Y538A_ and WT NAT10 (Supplemental Figure 17, A and C). The AlphaFold3 structure prediction showed high confidence of mutNAT10_exon4–5_, compared with WT NAT10 (Supplemental Figure 17D). Meanwhile, compared with control iTregs with pMigR1 and *Nat10*-deficient iTregs with WT *Nat10*, we observed that both the expressed mutants did not alter its protein localization in the differentiated iTregs (Supplemental Figure 16K). These results indicate that exon 4–5 deletion does not affect NAT10 protein structure, integrity, and localization in cells. Collectively, these findings suggest that NAT10 regulates the expression of iTreg activation- and suppression-associated genes through direct DNA binding, independent of its RNA acetyltransferase activity.

To evaluate whether NAT10 regulates antigen-specific activated CD4^+^ T cell differentiation, we crossed *Nat10*^fl/fl^ *Ox40*^cre^ mice with Smarta TCR transgenic mice (specific for LCMV GP61-80), generating Smarta/*Nat10^fl/fl^* and Smarta/*Nat10*^+/+^ *Ox40*^cre^ controls. CD45.2^+^ Smarta CD4^+^ T cells from these mice were transferred into CD45.1 recipient mice, followed by chronic LCMV-Cl13 infection to simulate a tumor-like microenvironment (Supplemental Figure 18A). At day 30 after infection, donor-derived CD4^+^ T cells showed no significant differences in the proportions of CXCR5^–^SLAM^hi^ Th1 cells and CXCR5^+^ SLAM^lo^ T follicular helper cells between the two groups (Supplemental Figure 18B). However, the frequency of FOXP3^+^ Tregs was markedly decreased in the *Nat10*-deficient group, despite no change in FOXP3 protein expression levels (Supplemental Figure 18, C and D). Western blot further revealed reduced phosphorylation of TCR signaling mediators ZAP70 and AKT in activated CD4^+^ T cells from the *Nat10*-deficient group, while total protein levels remained unchanged (Supplemental Figure 18E). Collectively, these findings suggest that *Nat10* deletion in OX40^+^ activated CD4^+^ T cells impairs iTreg differentiation in response to antigen stimulation, without affecting the expression of FOXP3 itself.

*NAT10* was induced in Treg subsets during iTreg differentiation (Figure 1D, clusters 1 and 3) and was essential for iTreg generation in tumor-bearing (Supplemental Figure 10, E–G) and chronic infection hosts (Supplemental Figure 18, A–D). Mechanistically, NAT10 promotes the expression of costimulatory molecules and members of the TNFR superfamily, thereby enhancing TCR signal transduction required for Treg activation and immunosuppressive function (Supplemental Figure 18F). Furthermore, anti–PD-1 immunotherapy and chemotherapy induce *Nat10* expression in TI Tregs (Figure 1I), accompanied by increased MHC-II expression in tumor cells (Supplemental Figure 4B). Together, these data suggest that NAT10 facilitates the differentiation of tumor antigen–activated CD4^+^ T cells into iTregs within the TME in response to immunotherapy or chemotherapy.

### Intratumoral Treg activation and immunosuppressive function are critically dependent on the NAT10 protein.

To evaluate NAT10’s role in TI Treg function, we treated B16F10-bearing C57BL/6 mice daily with the NAT10 inhibitor remodelin from day 4 after inoculation for 6 days (Supplemental Figure 19A). Remodelin did not affect body weight at either dosage (Supplemental Figure 19B) but markedly reduced NAT10 protein levels without altering FOXP3 expression versus the vehicle group (Supplemental Figure 19C), consistent with previous reports (43, 44). Remodelin dose-dependently delayed tumor growth and extended survival (Supplemental Figure 19, D–F). By day 14 after inoculation, the frequency and number of TI Tregs were markedly lower in groups treated with high doses (200 mg/Kg) of remodelin compared with those treated with low doses (100 mg/Kg) or vehicle (Supplemental Figure 19G). Additionally, the expression of key Treg markers, such as CD25 and PD-1, was reduced in a dose-dependent manner (Supplemental Figure 19, H and I). The frequency ratios of Tregs to CD8^+^ T cells and PD-1^+^ Tregs to PD-1^+^ CD8^+^ T cells were also diminished (Supplemental Figure 19, J and K).

To assess potential autoimmune effects of systemic *Nat10* deletion in Tregs, we administered tamoxifen to tumor-free *Nat10*^+/+^*Foxp3*^cre-ERT2^ and *Nat10*^fl/fl^*Foxp3*^cre-ERT2^ mice, and neither group showed signs of autoimmunity (Supplemental Figure 20, A–E). Moreover, remodelin treatment did not affect lung metastasis or subcutaneous tumor growth in *Rag1*^–/–^ mice (Supplemental Figure 20, F–I), indicating that remodelin’s antitumor effects are lymphocyte-dependent. Collectively, these results indicate that remodelin selectively and dose-dependently impairs TI Tregs, supporting the 200 mg/Kg dose for further studies.

To further dissect the pharmacological effects of NAT10 inhibition on TI Treg phenotype and function, we treated *Nat10*^+/+^*Foxp3*^cre-ERT2^ and *Nat10*^fl/fl^*Foxp3*^cre-ERT2^ mice with tamoxifen, with or without remodelin (Supplemental Figure 21A). Compared with the *Nat10*^+/+^*Foxp3*^cre-ERT2^ and vehicle group, *Nat10*^+/+^*Foxp3*^cre-ERT2^ and remodelin mice exhibited significantly fewer tumor nodules (Supplemental Figure 21B), reduced frequency and number of Tregs (Supplemental Figure 21C), and lower CD25 and PD-1 expression (Supplemental Figure 21, D and E). However, in *Nat10*^fl/fl^*Foxp3*^cre-ERT2^ mice, remodelin did not further reduce tumor burden, Treg frequency or number, or Treg-associated marker expression compared with vehicle (Supplemental Figure 21, B–E), suggesting that *Nat10* loss and pharmacological inhibition may converge on overlapping pathways. Importantly, cytotoxic CD8^+^ T cells from *Nat10*^+/+^*Foxp3*^cre-ERT2^ and remodelin, *Nat10*^fl/fl^*Foxp3*^cre-ERT2^ and vehicle, and *Nat10*^fl/fl^*Foxp3*^cre-ERT2^ and remodelin groups showed comparably elevated levels of effector molecules, including granzyme B, IFN-γ, perforin, and TNF-α, relative to the *Nat10*^+/+^*Foxp3*^cre-ERT2^ and vehicle control groups (Supplemental Figure 22, A–D). In summary, these findings demonstrate that NAT10 is essential for TI Treg expansion, activation, and immunosuppressive function. Pharmacological inhibition of NAT10 with remodelin effectively suppresses TI Treg activity and enhances antitumor CD8^+^ T cell responses, underscoring its therapeutic potential to overcome immune suppression in the TME.

### Pharmacological inhibition of NAT10 enhances anti–PD-1 immunotherapy by selectively targeting TI Tregs.

To assess the synergistic efficacy of NAT10 inhibition and anti–PD-1 immunotherapy in enhancing antitumor responses, B16F10-bearing C57BL/6 mice were treated with remodelin (200 mg/Kg) and/or anti–PD-1 antibody (200 μg) at indicated time points, starting on day 7 after tumor inoculation (Figure 7A). Anti–PD-1 alone moderately controlled tumor progression and prolong survival compared with vehicle. In contrast, both remodelin alone and its combination with anti–PD-1 significantly suppressed tumor growth and extended survival, with the combination treatment demonstrating the greatest efficacy (Figure 7, B and C). These results suggest that targeting NAT10 in Tregs can potentiate the effectiveness of immune checkpoint therapy in cancer. Flow cytometry analysis revealed that both remodelin alone and its combination with anti–PD-1 significantly reduced the frequency and number of TI Tregs, compared with vehicle or anti–PD-1 alone (Figure 7D). Immunofluorescence staining showed accumulation of NAT10^+^FOXP3^+^ Tregs surrounding tumor cells in lung tumors (Figure 7E). Notably, the frequency of NAT10^+^FOXP3^+^ Tregs was significantly lower in the remodelin-treated group than in the vehicle or anti–PD-1-treated groups, whereas anti–PD-1 alone had no effect on this population (Figure 7F). TI Tregs from combination-treated mice exhibited the lowest CD25 and PD-1 expression (Figure 7, G and H). Additionally, remodelin markedly reduced the ratio of Tregs to CD8^+^ T cells and the ratio of PD-1^+^ Tregs to PD-1^+^ CD8^+^ T cells (Figure 7, I and J), indicating enhanced CD8^+^ T cell activation. Furthermore, CD8^+^ T cells from combination-treated mice produced the highest levels of cytotoxic effectors, such as granzyme B, IFN-γ, perforin, and TNF-α (Supplemental Figure 23, A–D). This highlights the critical role of NAT10 inhibition in reversing Treg-mediated suppression of CD8^+^ T cell activity.

To further elucidate whether remodelin-induced TI Treg impairment depends on its direct effect on these cells, we treated *Nat10*^+/+^*Foxp3*^cre-ERT2^ and *Nat10*^fl/fl^*Foxp3*^cre-ERT2^ mice with tamoxifen, with or without anti–PD-1 (Supplemental Figure 24, A and B). The *Nat10*^fl/fl^*Foxp3*^cre-ERT2^ and anti–PD-1 groups showed a markedly reduced lung tumor burden compared with groups treated with monotherapies or controls (Supplemental Figure 24C), accompanied by decreased TI Treg frequency and number (Supplemental Figure 24D) and attenuated CD25 and PD-1 expression (Supplemental Figure 24, E and F). Combination therapy also led to a robust increase in functional CD8^+^ T cells, with the highest frequencies and protein levels of granzyme B, IFN-γ, perforin, and TNF-α compared with other treatment groups (Supplemental Figure 25, A–D), indicating reinvigoration of the antitumor CD8^+^ T cell compartment. In conclusion, NAT10 inhibition in TI Tregs is essential for overcoming their suppressive influence on cytotoxic CD8^+^ T cells. When combined with anti–PD-1 therapy, NAT10 blockade enhances antitumor immunity by suppressing Treg function and restoring CD8^+^ T cell activity. Thus, NAT10 represents a promising target to synergize with checkpoint blockade therapies in the TME.

## Discussion

Among RNA modifications, N6-methyladenosine (m6A) is the most extensively studied, while knowledge of ac4C remains limited. ac4C is a highly conserved eukaryotic RNA acetylation present in tRNA, rRNA, and mRNA (45). Human NAT10 is the key enzyme for ac4C formation; with both acetyltransferase and RNA-binding activity, it catalyzes ac4C at wobble cytidines to stabilize mRNA and enhance translation efficiency (46). ac4C is involved in oocyte maturation, aging, myocardial infarction, systemic lupus erythematosus, and cancers (35, 47–50). NAT10 is linked to progression of hepatocellular carcinoma, melanoma, bladder, and gastric cancers (50–53), but studies focus on tumor cells, with little exploration in immune cells especially Tregs. The role of NAT10-mediated mRNA acetylation in Treg function and its therapeutic potential in the TME are unclear.

This study shows NAT10 is critical for Treg immunosuppressive function. NAT10^+^ Tregs were found in human and murine lung tumors. Treg-specific *Nat10*-knockout mice showed suppressed tumor growth, with reduced TI Treg infiltration, increased fragility, and impaired suppressive capacity, enhancing antitumor immunity. IFN-γ–iTreg fragility is essential for effective anti–PD-1 therapy (17, 20). Consistently, *Nat10*-deficient Tregs upregulated IFN-γ, and NAT10 inhibition improved anti–PD-1 efficacy. Loss of PD-1/CXCR3 in Tregs weakens CD8^+^ T cell suppression (19, 37); similarly, *Nat10*-deficient Tregs failed to inhibit CD8^+^ T cells, driving antitumor responses. NAT10 is key for Treg-mediated CD8^+^ T cell suppression and tumor immune evasion. Mechanistically, our data raise the possibility that NAT10 facilitates Treg recruitment into the TME, likely via influencing local chromatin-loop contacts at target gene loci. In the future, many more HiC experiments and much more analysis will be required to further evaluate whether NAT10 broadly shapes chromatin-loop architecture across the genome.

High TI Treg levels correlate with poor cancer prognosis (6, 54). Selective intratumoral Treg depletion controls tumor growth without autoimmunity (16, 18, 55–57), but global Treg ablation paradoxically enhances immunosuppression (57, 58). Local/transient NAT10 inhibition may target TI Treg_s_ while preserving systemic immune tolerance. NAT10 binding sites contain a conserved 46-nucleotide high-GC motif prone to R-loop formation (59). NAT10 acts as a cofactor with FOXP3 to promote Treg costimulatory molecules and TNFR superfamily expression. Treg-specific *Nat10* deletion reduced H3K27ac, chromatin accessibility, and FOXP3 binding at Treg functional genes. NAT10 may recruit FOXP3 to drive Treg differentiation and function, representing a conceptual advance for immunotherapy. NAT10 supports iTreg division, effector state, and expression of PD-1, 4-1BB, GITR, OX40, and ICOS. Anti–PD-1 or chemotherapy (cisplatin, paclitaxel) markedly induced *Nat10* in TI Tregs. In the TME, these treatments upregulate tumor MHC-II, enhancing antigen presentation to CD4^+^ T cells and promoting iTreg differentiation (60). NAT10/FOXP3 cooperatively regulate Treg costimulatory molecules, supported by human in vitro data (no human cancer Treg functional assays yet). Additionally, given that B16F10 and LLC models were used to assess the broader immunoregulatory and therapeutic effects of our approach in immunocompetent tumor models, rather than to fully model NSCLC-specific disease biology, this represents a limitation of the present study, and further in-depth investigations are required in future work.

Immune checkpoint blockade–iTreg activation limits immunotherapy efficacy. IL-2 analogs combined with checkpoint blockade enhance CD8^+^ T cell activity (61–63), but TI Tregs remain a major immunosuppressive factor. Systemic Treg inhibition causes severe autoimmunity, and TI Treg expansion drives therapy resistance. Anti–PD-1/chemotherapy induce *Nat10* in TI Tregs, and NAT10 is required for iTreg differentiation and function (not CD4^+^ T cell activation), sustaining Treg proliferation in the TME. NAT10 inhibitors suppress NAT10^+^ Treg expansion, supporting targeted TI Treg therapy. Combining IL-2 analogs and NAT10 inhibitors warrants further study. Future studies using NSCLC-relevant immunocompetent or genetically engineered lung cancer models will be necessary to further validate the therapeutic relevance of this strategy in NSCLC.

Inhibiting NAT10 destabilizes TI Tregs to trigger antitumor immunity. Combining NAT10 inhibition with anti–PD-1 improves tumor control and may increase patient response rates to immunotherapy/chemotherapy.

## Methods

Additional methods may be found in the supplemental materials.

### Sex as a biological variable.

Both sexes were included in animal and human studies, but sex was not considered as a biological variable; no sex‑based analyses were performed, and the findings are reported without sex stratification.

### Mice.

*Nat10*^fl/fl^ mice were generated by GemPharmatech Co. Ltd. The *Rag1*^–/–^, B6.SJL (CD45.1), C57BL/6, *Foxp3*^GFP-cre-ERT2^, *Cd4*^cre^, *Ox40*^cre^ (*Tnfrsf4*^cre^), and Smarta mice were obtained from The Jackson Laboratory.

### Human samples.

Primary tumor tissues from 19 human patients with NSCLC were obtained from the Affiliated Cancer Hospital and Institute of Guangzhou Medical University.

### Cell lines.

The B16F10, LLC and HEK293T cell lines were provided by Rongbin Zhou at University of Science and Technology of China. All cell lines were cultured at 37°C under 5% CO_2_ in RPMI 1640 or DMEM medium (BI), supplemented with 10% fetal bovine serum (BI) and 1% Penicillin-Streptomycin (Gibco).

### Statistics.

GraphPad Prism 8.0.2 was used for data analysis, and results are presented as mean ± SEM. Two-group comparisons were performed using 2-tailed unpaired Student’s *t* test, while multiple‑group comparisons were assessed by 1-way ANOVA. Tumor or weight growth curves were analyzed by 2-way ANOVA. Survival curves were compared using log-rank test. *P* values of less than 0.05 were considered significant.

### Study approval.

All animal experiments were reviewed and approved by the Ethics Committee of the University of Science and Technology of China (approval USTCACUC27120125018). Human tumor tissue samples were obtained from patients who provided written informed consent, under protocols approved by the Ethics Committee of the Affiliated Cancer Hospital and Institute of Guangzhou Medical University (approval GYZL-2024-KY20).

### Data availability.

The ATAC-seq, CUT&Tag, whole-genome bisulfite sequencing (WGBS), ac4C RIP-seq, RNA-seq, and high-resolution chromosome-conformation capture (Hi-C) data are deposited at the Gene Expression Omnibus under accession GSE254341. Values for all data points in graphs are reported in the Supporting Data Values file.

## Manuscript information

### Author contributions.

XQ performed most experiments and data analysis with help from HM, Jiabao Wang, JS, JM, JY, HY, and YH. DC and JL analyzed the ATAC-seq, CUT&Tag, WGBS, ac4C RIP-seq, RNA-seq, Hi-C, and scRNA-seq data. HC prepared *Foxp3*^GFP-cre-ERT2^ mice. S Luo and Jia Wang supported Hi-C library construction. WG and BW purified the NAT10 protein; XL and S Li performed the EMSA and acetyltransferase activity assay. WF and QW provided clinical samples. FL, JL, KZ, BL, and MC supervised the overall study and compiled the manuscript. The order of the co–first authors was determined by their relative contributions to the study. XQ performed most of the experiments and data analysis and is therefore listed first. DC analyzed the ATAC-seq, CUT&Tag, WGBS, ac4C RIP-seq, RNA-seq, Hi-C, and scRNA-seq data and is listed second. XL performed the EMSA and acetyltransferase activity assay and is listed third. WF provided the clinical samples and is listed fourth. HM performed immunofluorescence staining of human and mouse tumor tissues and is listed last as the fifth co-first author. All authors have reviewed and approved this author order.

### AI disclosure.

AI was used for two purposes. First, AI was used for protein structure prediction using AlphaFold3, from July 1, 2024 to July 3, 2024. Second, AI was used for language polishing of the manuscript using DeepSeek-V3, in 2026. No AI tools were used for research design, data extraction, analysis, or synthesis, nor for the preparation of figures, graphics, or other content.

### Conflict of interest.

The authors have declared that no conflict of interest exists.

### Funding.

This study was supported by the following:

- University of Science and Technology of China Research Funds of the Double First-Class Initiative (YD9100002508 to FL).
- Center for Advanced Interdisciplinary Science and Biomedicine of IHM (QYPY20230027 to FL).
- Ministry of Science and Technology of China (2023YFC2306400 to FL, 2022YFA1302700 to KZ, and 2022YFC2303700 to KZ and S Li).
- National Natural Science Foundation of China (32371345 to KZ, 82370148 to JL, 32301044 and 32471301 to S Li).
- Anhui Provincial Natural Science Foundation (2308085QC80 to SL).
- Center for Advanced Interdisciplinary Science and Biomedicine of IHM (QYPY20220019 to KZ).
- Medical Scientific Research Foundation of Guangdong (A2024277 to QW).
- R&D Program of Guangzhou National Laboratory (GZNL2023A02011 to JL).

## Supplementary Material

Supplemental data

Supplemental data set 1

Unedited blot and gel images

Supporting data values

## Figures and Tables

**Figure 1:**
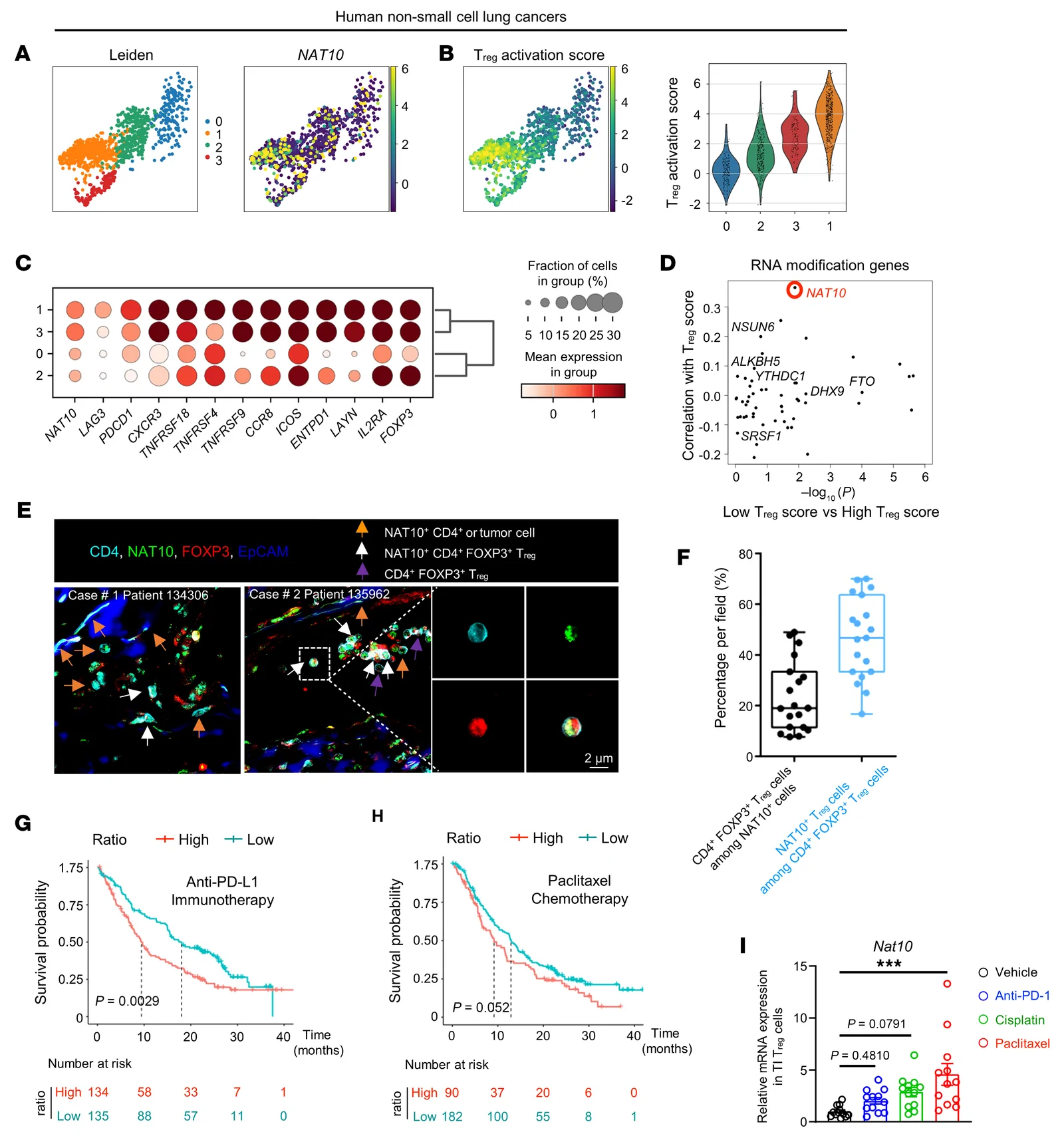
Increased NAT10 expression in Tregs in human and mouse cancer. (**A**) UMAP representation of the cells subclustered by CD4^–^FOXP3^–^CTLA4 Tregs (1,146 cells) expressing *NAT10* in the GSE99254 dataset. (**B**) Calculating the Treg activation score for each cell using classical Treg activation markers (left: UMAP, right: Violin plot). (**C**) Dot plot showing the classical Treg activation markers in the *NAT10* differentiated expression clusters. (**D**) Comparison of the correlation coefficient between the expression of each RNA epigenetic modification gene and its Treg activation score. (**E**) Immunofluorescence staining of human NSCLC tumor sections used the markers CD4, NAT10, FOXP3, and EpCAM to identify CD4^+^ T cells, CD4^+^FOXP3^+^ Tregs, NAT10^+^CD4^+^FOXP3^+^ Tregs, and EpCAM^+^ tumor cells. Scale bar: 2 μm. (**F**) Percentage per field of CD4^+^FOXP3^+^ Tregs among NAT10^+^ cells or NAT10^+^ Tregs among CD4^+^FOXP3^+^ Tregs (*n* = 19). (**G** and **H**) Kaplan-Meier plot of survival analysis using *NAT10* expression in anti–PD-L1 immunotherapy (**G**) and paclitaxel chemotherapy (**H**) of the NSCLC cohort. (**I**) Real-time PCR showing relative mRNA expression of *Nat10* in TI Tregs after anti–PD-L1, cisplatin, or paclitaxel treatment (*n* = 12). Data represent 2–3 independent experiments; mean ± SEM. *P* values were calculated using 1-way ANOVA (**I**). ***P < 0.001.

**Figure 2:**
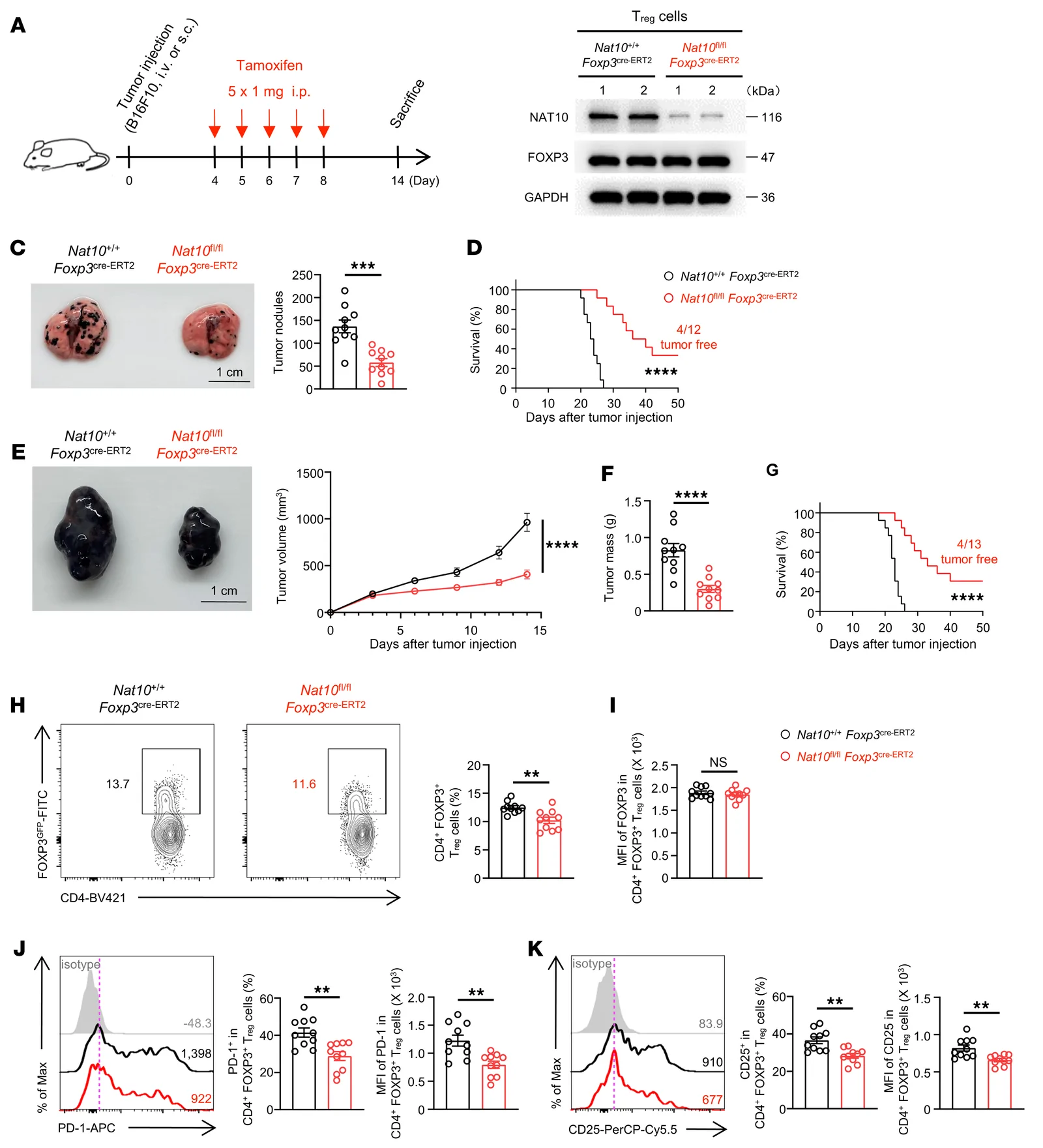
*Nat10* deficiency in TI Tregs shows delayed tumor growth and prolonged survival. (**A**) Schematic of the experiment. (**B**) Western blot for the NAT10 protein level in Tregs from *Nat10*^+/+^ *Foxp3*^cre-ERT2^ and *Nat10*^fl/fl^ *Foxp3*^cre-ERT2^ mice treated with tamoxifen. (**C**) Quantification of tumor nodules in the lungs of *Nat10*^+/+^ *Foxp3*^cre-ERT2^ and *Nat10*^fl/fl^ *Foxp3*^cre-ERT2^ mice at day 14 after intravenous injection with B16F10 cells (*n* = 10). Scale bar: 1 cm. (**D**) Survival curve of *Nat10*^+/+^ *Foxp3*^cre-ERT2^ and *Nat10*^fl/fl^ *Foxp3*^cre-ERT2^ mice intravenously injected with B16F10 cells (*n* = 12). (**E** and **F**) Tumor growth rate (**E**) and tumor mass (**F**) in *Nat10*^+/+^ *Foxp3*^cre-ERT2^ and *Nat10*^fl/fl^ *Foxp3*^cre-ERT2^ mice at day 14 after subcutaneous injection with B16F10 cells (*n* = 10). Scale bar: 1 cm. (**G**) Survival curve of *Nat10*^+/+^ *Foxp3*^cre-ERT2^ and *Nat10*^fl/fl^ *Foxp3*^cre-ERT2^ mice subcutaneously injected with B16F10 cells (*n* = 13). (**H** and **I**) Representative flow cytometry plots (left) and quantification of the percentage (right) of TI Tregs (**H**) and MFI of FOXP3 (**I**) in *Nat10*^+/+^ *Foxp3*^cre-ERT2^ and *Nat10*^fl/fl^ *Foxp3*^cre-ERT2^ mice intravenously injected with B16F10 cells (*n* = 10). (**J** and **K**) Representative histograms (left) and quantification of the percentages (middle) and MFI (right) of PD-1 (**J**) and CD25 (**K**) in TI Tregs from *Nat10*^+/+^ *Foxp3*^cre-ERT2^ and *Nat10*^fl/fl^ *Foxp3*^cre-ERT2^ mice intravenously injected with B16F10 cells (*n* = 10). Data represent 2–3 independent experiments; mean ± SEM. *P* values were calculated using 2-tailed unpaired Student’s *t* test (**C**, **F**, and **H**–**K**), 2-way ANOVA (**E**), and log-rank test (**D** and **G**). **P < 0.01, ****P < 0.0001.

**Figure 3:**
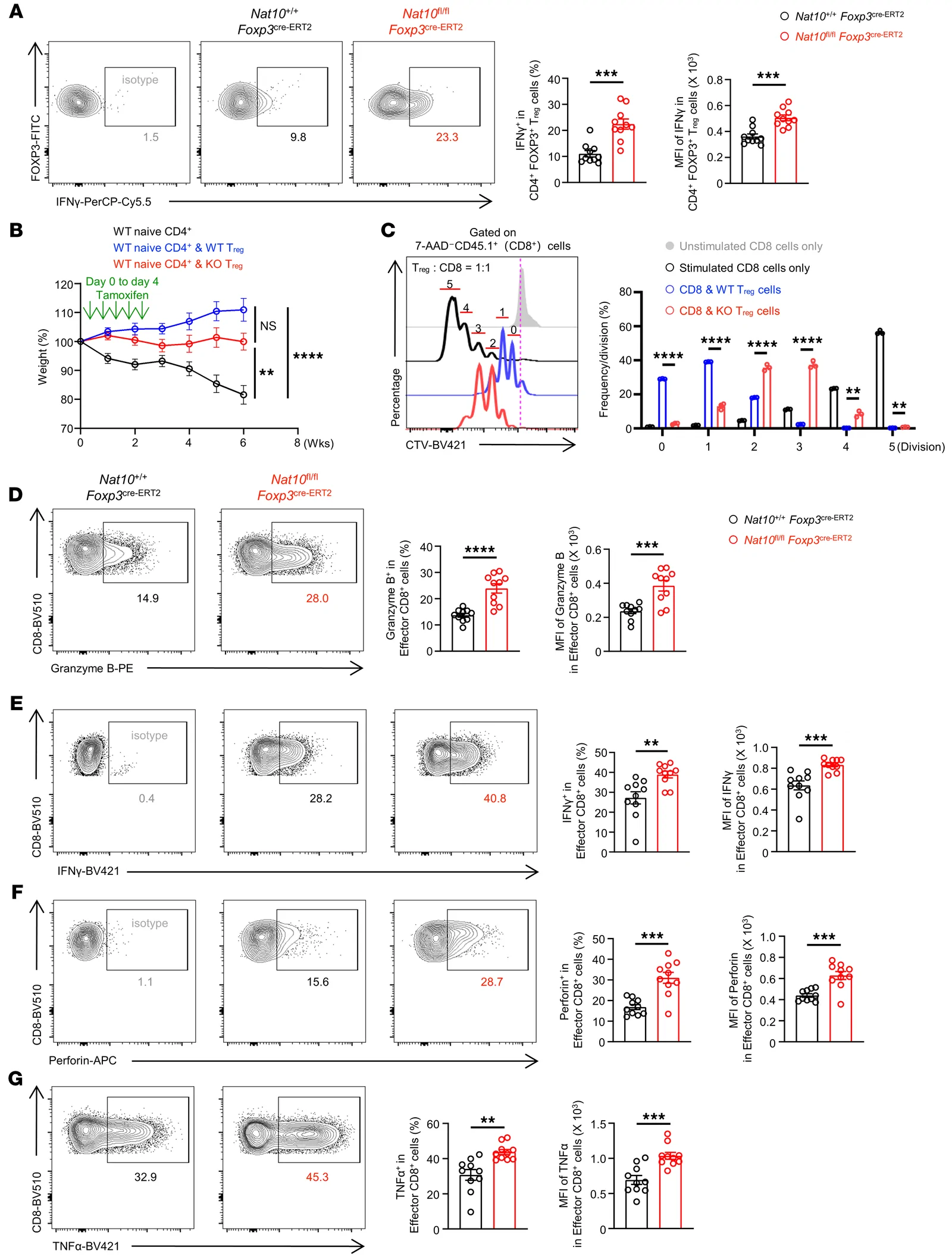
NAT10 is essential for preventing the fragility and suppressive function of Tregs. (**A**) Representative flow cytometry plots (left) and quantification of the percentages (middle) and MFI (right) of IFN-γ in TI Tregs after ex vivo stimulation with PMA and ionomycin from *Nat10*^+/+^ *Foxp3*^cre-ERT2^ and *Nat10*^fl/fl^ *Foxp3*^cre-ERT2^ mice intravenously injected with B16F10 cells (*n* = 10). (**B**) Body weight changes in *Rag1*^−/−^ recipient mice transferred with naive conventional CD4^+^ cells isolated from the spleens of CD45.1 mice alone or with Tregs isolated from the spleens of *Nat10*^+/+^ *Foxp3*^cre-ERT2^ and *Nat10*^fl/fl^ *Foxp3*^cre-ERT2^ mice (*n* = 6). *Rag1*^−/−^ mice were intraperitoneally injected with tamoxifen as indicated. (**C**) Representative histogram of CTV (left) and quantification of the percentages (right) of each division in naive CD8^+^ T cells isolated from the spleens of CD45.1 mice. Cells were cultured for 72 hours alone or with TI Tregs isolated from *Nat10*^+/+^ *Foxp3*^cre-ERT2^ and *Nat10*^fl/fl^ *Foxp3*^cre-ERT2^ mice in the presence or absence of anti-CD3 and anti-CD28 antibodies (*n* = 3). (**D**–**G**) Representative flow cytometry plots (left) and quantification of the percentages (middle) and MFI (right) of granzyme B (**D**), IFN-γ (**E**), perforin (**F**), and TNF-α (**G**) in TI CD8^+^ cells after ex vivo stimulation from *Nat10*^+/+^ *Foxp3*^cre-ERT2^ and *Nat10*^fl/fl^ *Foxp3*^cre-ERT2^ mice intravenously injected with B16F10 cells (*n* = 10). Data represent 2–3 independent experiments; mean ± SEM. *P* values were calculated using 2-tailed unpaired Student’s *t* test (**A** and **C**–**G**) and 2-way ANOVA (**B**). **P < 0.01, ***P < 0.001, ****P < 0.0001.

**Figure 4:**
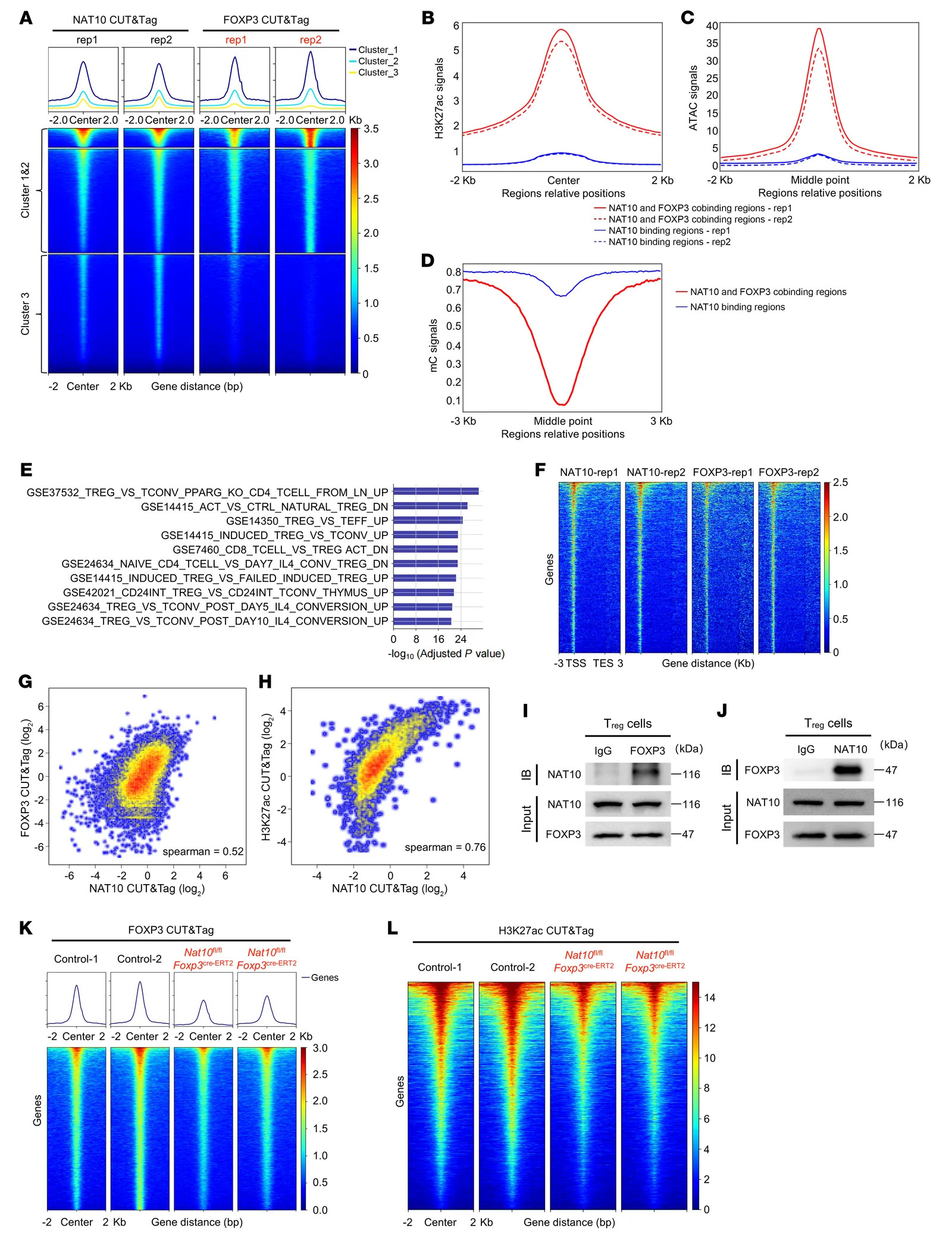
NAT10 interacts with FOXP3 to directly regulate the activation of TI Tregs. (**A**) Heatmaps showing NAT10 and FOXP3 CUT&Tag peaks resolved into 3 clusters by K-means clustering. (**B**) Curve plot showing the H3K27ac CUT&Tag signals in clusters 1 and 2 (red) and cluster 3 (blue) peak region. (**C**) Curve plot displaying the chromosome accessibility signals in clusters 1 and 2 (red) and cluster 3 (blue) peak region. (**D**) Curve plot displaying the mean methylated cytosine signals in clusters 1 and 2 (red) and cluster 3 (blue) peak region. (**E**) Gene set enrichment analysis of the candidate genes within 2,000 of clusters 1 and 2 peak regions. (**F**) Heatmaps showing the NAT10 and FOXP3 CUT&Tag signals enriched in the promoter regions of Treg signature genes. (**G**) Scatterplot showing the correlation between the NAT10 and FOXP3 enrichment signals on H3K27ac peak regions. (**H**) Scatterplot showing the correlation between the NAT10 and H3K27ac genome-wide enrichment signals. (**I**) FOXP3 pull-down NAT10 in TI Tregs by the co-IP experiment. (**J**) NAT10 pull-down FOXP3 in TI Tregs by the co-IP experiment. (**K**) Heatmaps exhibiting reduced FOXP3 peaks in *Nat10*^fl/fl^ *Foxp3*^cre-ERT2^ TI Tregs. (**L**) Heatmaps showing reduced H3K27ac enrichment in *Nat10*^fl/fl^ *Foxp3*^cre-ERT2^ TI Tregs.

**Figure 5:**
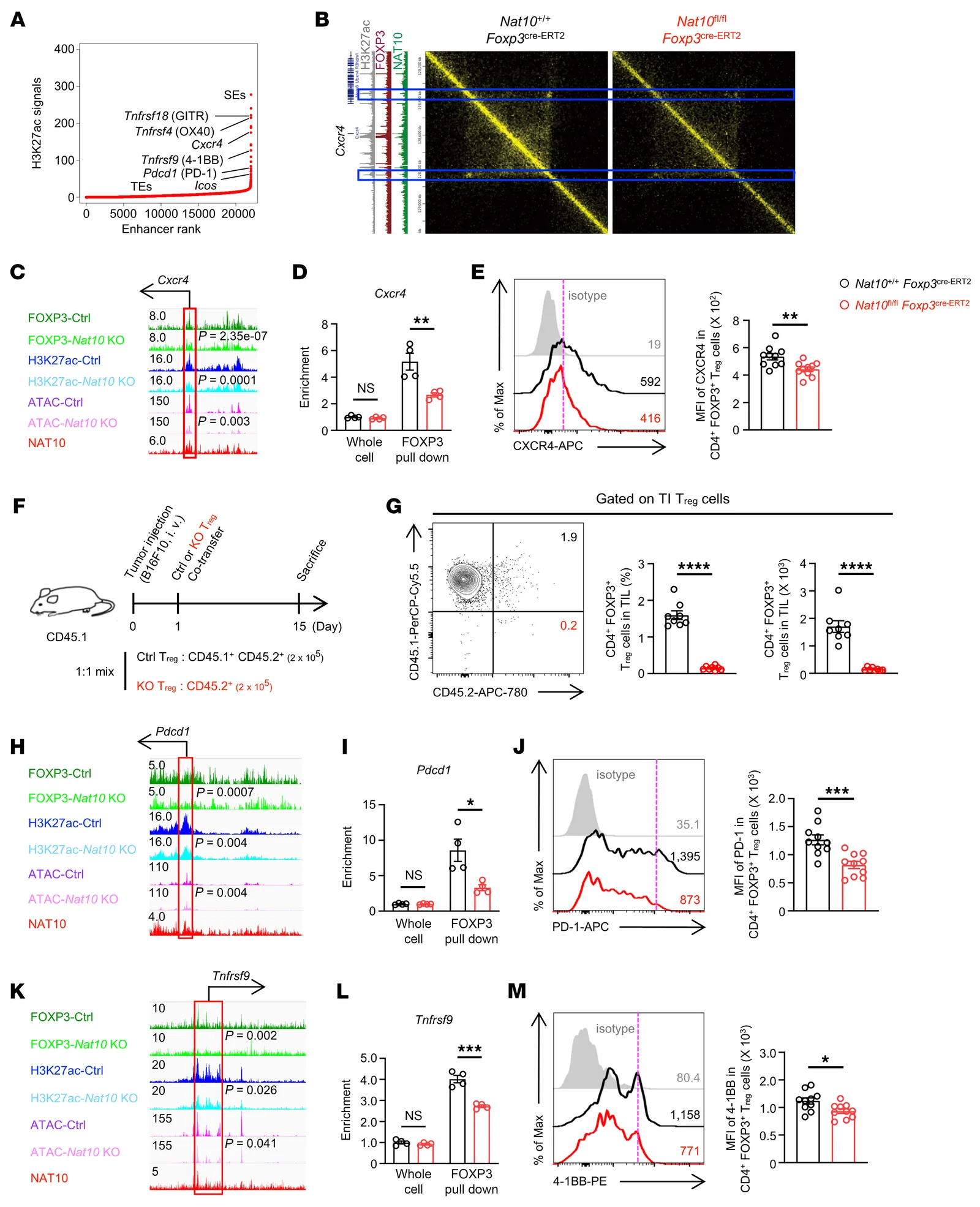
NAT10 interacts with FOXP3 to facilitate the Treg superenhancer establishment. (**A**) Analysis of TI Tregs in Figure 4. Treg-specific super-enhancers are associated with Treg signature genes, including *Tnfrsf18* (GITR), *Tnfrsf4* (OX40), *Cxcr4*, *Tnfrsf9* (4-1BB), *Pdcd1* (PD-1), and *Icos*. (**B**) Chromosome loops with anchors cobinding with NAT10 and FOXP3 in WT and *Nat10*-deficient Tregs. Representative Hi-C contact maps at the *Cxcr4*-containing region in WT and *Nat10*-deficient Tregs at 5 kb resolution. Blue boxes highlight the local region used for comparison. (**C**) NAT10-CUT&Tag, FOXP3-CUT&Tag, H3K27ac-CUT&Tag, and ATAC-seq tracks at the *Cxcr4* gene locus. (**D**) qPCR showing the enrichment of FOXP3 at the promoter of *Cxcr4* in TI Tregs (*n* = 4). (**E**) Representative histogram (left) and quantification of the MFI (right) of CXCR4 in TI Tregs (*n* = 10). (**F**) Schematic of the experiment. (**G**) Representative flow cytometry plot (left) and quantification of the percentages (middle) and number (right) of TI Tregs in lung tumors of the CD45.1 mice adoptively transferred with WT or *Nat10*-deficient Tregs (*n* = 8). (**H**) NAT10-CUT&Tag, FOXP3-CUT&Tag, H3K27ac-CUT&Tag, and ATAC-seq tracks at the *Pdcd1* gene locus. (**I**) qPCR showing the enrichment of FOXP3 at the promoter of *Pdcd1* in TI Tregs (*n* = 4). (**J**) Representative histogram (left) and quantification of the MFI (right) of PD-1 in TI Tregs (*n* = 10). (**K**) NAT10-CUT&Tag, FOXP3-CUT&Tag, H3K27ac-CUT&Tag, and ATAC-seq tracks at the *Tnfrsf9* gene locus. (**L**) qPCR showing the enrichment of FOXP3 at the promoter of *Tnfrsf9* in TI Tregs (*n* = 4). (**M**) Representative histogram (left) and quantification of the MFI (right) of 4-1BB in TI Tregs (*n* = 10). Data represent 2–3 independent experiments; mean ± SEM. *P* values were calculated using 2-tailed unpaired Student’s *t* test (**D**, **E**, **G**, **I**, **J**, **L**, and **M**). *P < 0.05, **P < 0.01, ***P < 0.001, ****P < 0.0001.

**Figure 6:**
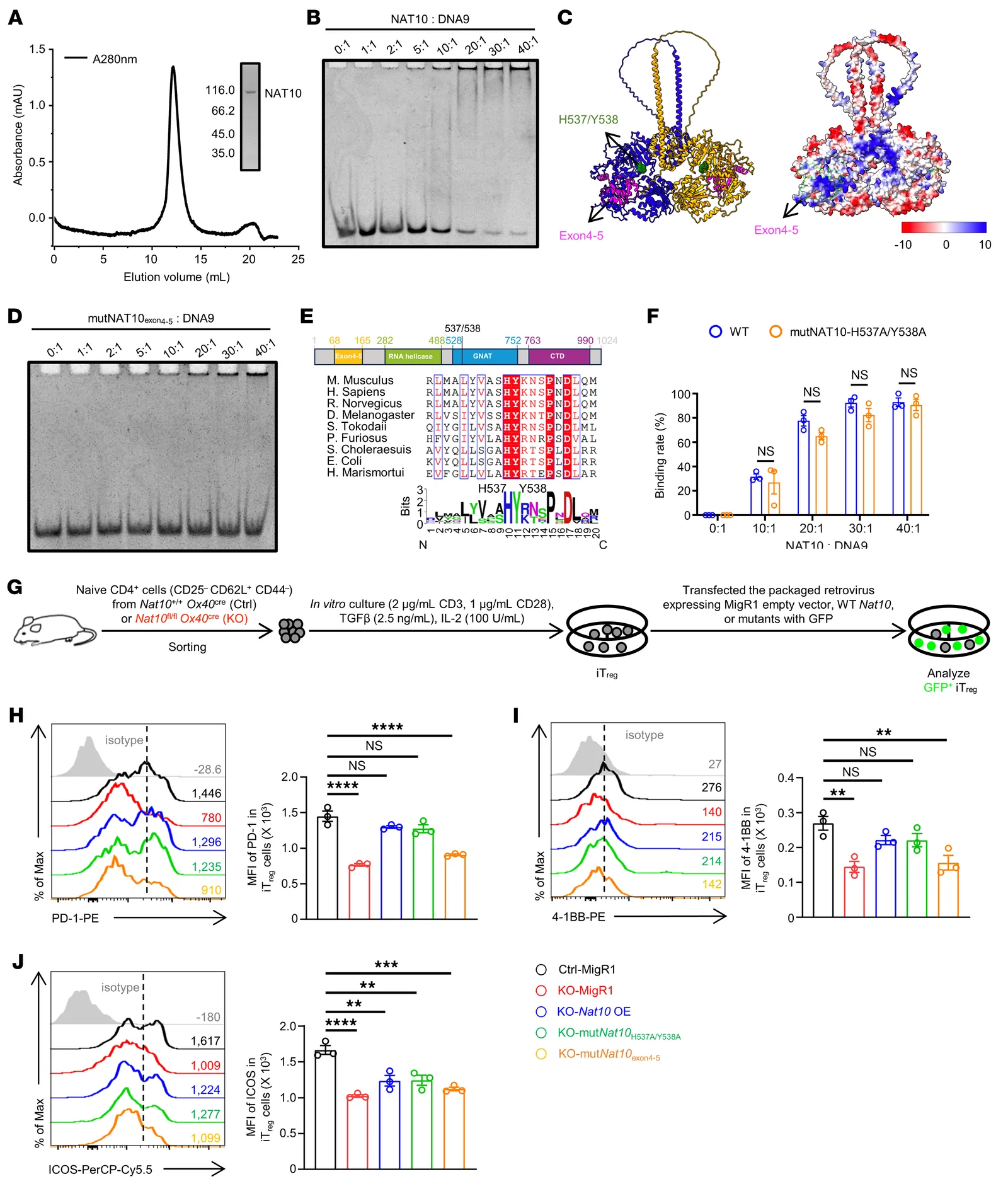
NAT10 promotes iTreg differentiation by positively transcriptional regulation of activation-associated genes. (**A**) Size-exclusion chromatography analysis of mouse NAT10. (**B**) EMSA analysis of WT NAT10 binding to DNA. (**C**) Left: Three-dimensional structure of dimerized NAT10 predicted by AlphaFold3. Two monomers of NAT10 are shown in blue and orange. Right: Surface electrostatic potential of the NAT10 dimer. The positively charged residues and acetyl-CoA-binding residues are colored in pink and green, respectively. (**D**) EMSA analysis of mutant NAT10 (exon 4–5 deletion) binding to DNA. (**E**) Sequence conservation analysis. The sequence logo, which depicts the relative frequency of amino acids at each position in a multiple sequence alignment, includes color-coded residues based on their chemical properties. (**F**) Compared with the WT NAT10 protein, the EMSA analysis of mutant NAT10 (H537A/Y538A) binding to DNA. The binding rate was determined by calculating the proportion of remaining free DNA, as measured by a grayscale value obtained using ImageJ (NIH) (*n* = 3). (**G**) The schematic of the experiment. (**H**–**J**) Representative histograms (left) and quantification of the MFI (right) of PD-1 (**H**), 4-1BB (**I**), and ICOS (**J**) in iTregs of *Nat10*^+/+^ *Ox40*^cre^ and *Nat10*^fl/fl^ *Ox40*^cre^ mice (*n* = 3). Data represent 2–3 independent experiments; mean ± SEM. *P* values were calculated using 2-tailed unpaired Student’s *t* test (**F**) and 1-way ANOVA (**H**–**J**). **P < 0.01, ***P < 0.001, ****P < 0.0001.

**Figure 7:**
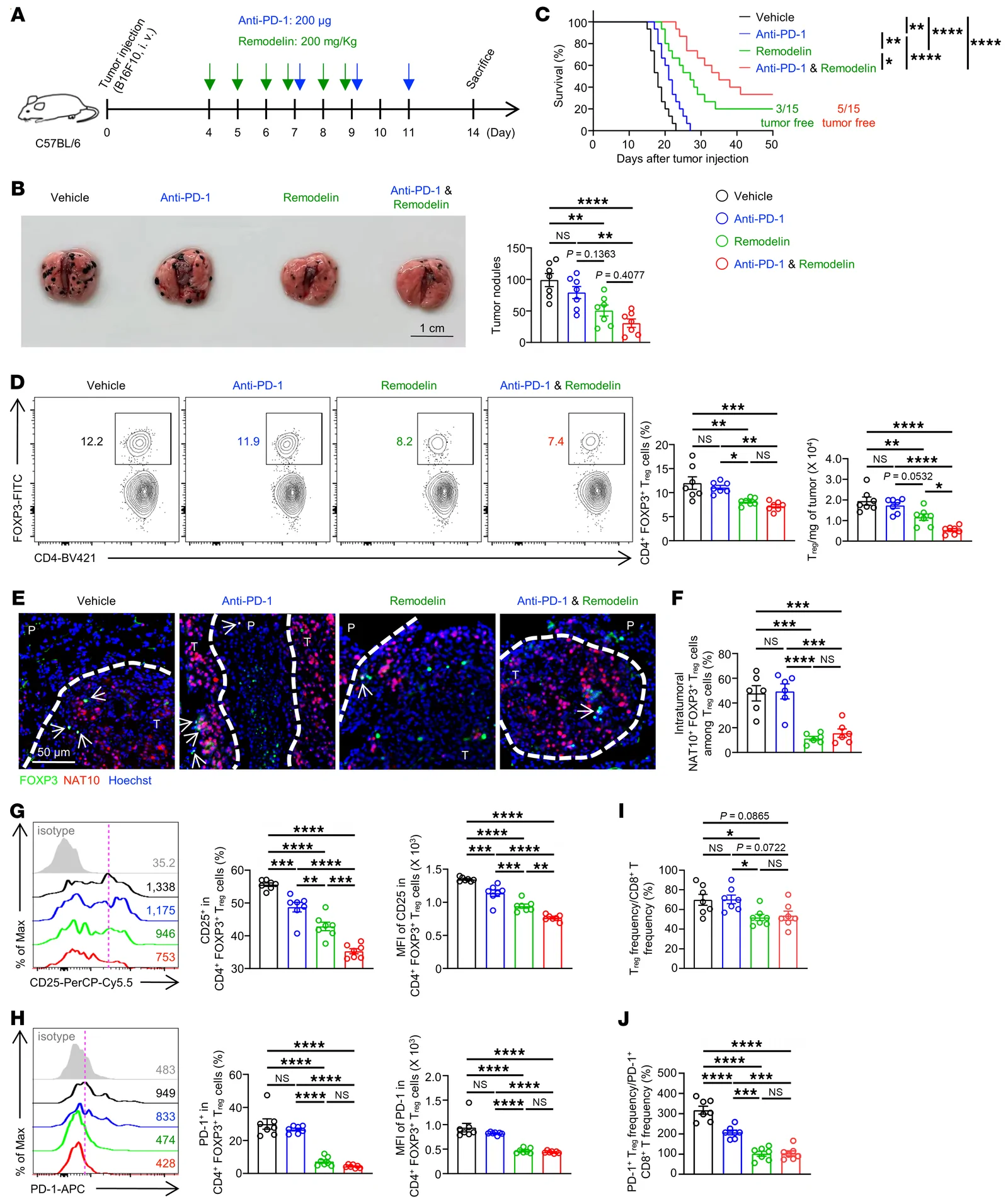
Inhibition of NAT10 synergizes with anti–PD-1 therapy to enhance control of tumor growth. (**A**) Schematic of the experiment. (**B**) Quantification of tumor nodules in the lungs of C57BL/6 mice treated with vehicle, anti–PD-1, remodelin, and anti–PD-1 and remodelin at day 14 after intravenous injection with B16F10 cells (*n* = 7). Scale bar: 1 cm. (**C**) Survival curve of B16F10 tumor-bearing C57BL/6 mice treated with vehicle, anti–PD-1, remodelin, and anti–PD-1 and remodelin (*n* = 15). (**D**) Representative flow cytometry plots (left) and quantification of the percentage (middle) and number (right) of TI Tregs in B16F10 tumor-bearing C57BL/6 mice treated with vehicle, anti–PD-1, remodelin, and anti–PD-1 and remodelin (n = 7). (**E** and **F**) Representative immunofluorescence staining (**E**) and quantification of the percentage (**F**) of intratumoral NAT10^+^ FOXP3^+^ Tregs (*n* = 6). P, peri-tumoral tissue; T, tumor tissue. (**G** and **H**) Representative histograms (left) and quantification of the percentages (middle) and MFI (right) of CD25 (**G**) and PD-1 (**H**) in TI Tregs from B16F10 tumor-bearing C57BL/6 mice treated with vehicle, anti–PD-1, remodelin, and anti–PD-1 and remodelin (*n* = 7). (**I** and **J**) Ratio of Tregs to CD8^+^ T cells (**I**) and PD-1^+^ Tregs to CD8^+^ T cells (**J**) in B16F10 tumor-bearing C57BL/6 mice treated with vehicle, anti–PD-1, remodelin, and anti–PD-1 and remodelin (*n* = 7). Data represent 2–3 independent experiments; mean ± SEM. *P* values were calculated using 1-way ANOVA (**B**, **D**, and **F**–**J**) and log-rank test (**C**). *P < 0.05, **P < 0.01, ***P < 0.001, ****P < 0.0001.

## Version 1 11/02/2026

Electronic publication
