# Supplementary material for: Targeting N-acetyltransferase 10 reprograms Treg function to enhance anti–PD-1 immunotherapy sensitivity in murine lung tumors
Source: J Clin Invest. 2026 Nov 2;136(21):e189034. doi: 10.1172/JCI189034 (PMC13722382; doi:10.1172/JCI189034)
Supplement: Supplementary file 1 [file jci-136-189034-s239.pdf]

Supplemental Methods

Tamoxifen treatment

Tamoxifen (1 g; Sigma-Aldrich) was suspended in 10 mL of absolute ethanol and 90 mL of olive oil (Aladdin). Mice were intraperitoneally injected with 1 mg tamoxifen per mouse at indicated time points.

Tumor models and treatments

For subcutaneous models, B16F10 cells (2×10^5) or LLC cells (1×10^6) were injected subcutaneously into mice. Tumor volume was calculated as length × width × width / 2 every 2-3 days. For metastasis models, B16F10 cells (2×10^5) or LLC cells (1×10^6) were injected intravenously via the tail vein. Mice were euthanized on day 14 post-tumor cell injection, and visible nodules on lung lobes were quantified.

Remodelin (Selleck) was dissolved in DMSO and diluted in Tween 80 (Sigma Aldrich) and 2-hydroxypropyl-β-cyclodextrin (Sigma Aldrich) to a final formulation of 20% DMSO, 15% Tween 80, and 65% cyclodextrin, then administered intragastrically at 100 or 200 mg/Kg at indicated time points. Anti-PD-1 antibody (clone 29F.1A12, Bio X Cell, BE0273) or rat IgG2a isotype control (clone 2A3, Bio X Cell, BE0089) was injected intraperitoneally at 200 μg per mouse in 200 μL PBS at indicated time points. Cisplatin (Selleck) was injected intraperitoneally at 5 mg/Kg per mouse in saline at indicated time points. Paclitaxel (Selleck) was dissolved in DMSO and diluted in PEG300 (MCE), Tween 80 (Sigma Aldrich), and ddH_2O to a final formulation of 5% DMSO, 40% PEG300, 5% Tween 80, and 50% ddH_2O, then administered

intraperitoneally at 10 mg/Kg at indicated time points.

Preparation of single-cell suspensions, antibody staining, and flow cytometry

To obtain tumor and lung tissue-infiltrating cells, mice were euthanized, and the tumor and lung were meticulously removed and mechanically cut into small pieces using surgical scissors. Digestion of the tumor and lung in DMEM medium supplemented with 1 mg/mL collagenase type IV and 10 U/mL DNase I was performed for 1 hour or 30 minutes at 37 °C in a shaking incubator (200 rpm). Leukocytes were isolated by suspension in 40% percoll and subjected to density gradient centrifugation. The resulting cell suspension was filtered using a 70 μm cell strainer. Isolated cells were utilized for various assays. The spleen and lymph nodes were crushed through a 70 μm cell strainer, and red blood cells were lysed with RBC lysis buffer. Bone marrow cells were obtained by inserting a sterile syringe into the bone marrow cavity and rinsing with PBS, and red blood cells were lysed with RBC lysis buffer.

Single-cell suspensions were stained with fluorochrome-conjugated antibodies. For intracellular cytokine staining, cells were initially incubated for 5-6 hours with 50 ng/mL PMA (Sigma-Aldrich), 1 μg/mL ionomycin (Sigma-Aldrich), and 1:1000 monensin at 37 °C in RPMI 1640 medium containing 10% FBS. Following surface antibody labeling, cells were fixed using the transcription factor buffer set (BD Pharmingen) or Foxp3/transcription factor staining buffer set (eBioscience) according to the manufacturer's instructions and subsequently stained with antibodies. After the washing step, cells were collected using a flow cytometer (Beckman CytoFLEX) and

analyzed with FlowJo V10 software.

Staining utilized the following antibody: PerCP-Cy5.5 anti-CD3e (145-2C11, BD, 561108), FITC anti-CD4 (RM4-5, BioLegend, 100510), PE anti-CD4 (RM4-5, BioLegend, 100512), BV421 anti-CD4 (RM4-5, BD, 740007), BV510 anti-CD4 (RM4-5, BD, 563106), PE anti-CD8 (53-6.7, BioLegend, 100708), BV510 anti-CD8 (53-6.7, BD, 563068), PE-Cy7 anti-CD11c (HL3, BD, 558079), PE anti-CD25 (PC61.5, eBioscience, 12-0251-83), PerCP-Cy5.5 anti-CD25 (PC61.5, eBioscience, 45-0251-82), PE-Cy7 anti-CD25 (PC61.5, eBioscience, 25-0251-82), APC anti-CD25 (PC61.5, eBioscience, 17-0251-82), APC-Cy7 anti-CD25 (PC61.5, BioLegend, 102025), PerCP-Cy5.5 anti-CD44 (IM7, BD, 560570), PE-Cy7 anti-CD44 (IM7, BD, 560569), BV510 anti-CD45 (30-F11, BD, 563891), PerCP-Cy5.5 anti-CD45.1 (A20, BioLegend, 110728), APC anti-CD45.1 (A20, BioLegend, 110714), PerCP-Cy5.5 anti-CD45.2 (104, BioLegend, 109827), PE-Cy7 anti-CD45.2 (104, eBioscience, 25-0454-82), APC-eFluor 780 anti-CD45.2 (104, eBioscience, 47-0454-82), PE anti-CD45RB (C363-16A, BioLegend, 103307), PE anti-CD62L (MEL-14, BioLegend, 104407), PerCP-Cy5.5 anti-CD62L (MEL-14, eBioscience, 45-0621-82), APC anti-CD69 (H1.2F3, BD, 560689), APC anti-OX40/CD134 (OX-86, eBioscience, 17-1341-82), PE anti-4-1BB/CD137 (17B5, eBioscience, 12-1371-82), APC anti-CD146 (ME-9F1, BioLegend, 134712), PE-Cy7 anti-SLAM/CD150 (TC15-12F12.2, BioLegend, 115914), BV421 anti-CTLA4/CD152 (UC10-4B9, BioLegend, 106312), PE-Cy7 anti-CXCR3/CD183 (CXCR3-173, BioLegend, 126516), APC anti-CXCR4/CD184 (2B11, eBioscience, 17-9991-82), BV421 anti-CXCR5/CD185 (L138D7, BioLegend, 145512), PerCP-Cy5.5

anti-ICOS/CD278 (C398.4A, BioLegend, 313518), PE anti-PD-1/CD279 (J43, eBioscience, 12-9985-83), APC anti-PD-1/CD279 (J43, BD, 562671), BV421 anti-PD-1/CD279 (29F.1A12, BioLegend, 135221), PE anti-NRP1/CD304 (3DS304M, eBioscience, 12-3041-82), APC anti-GITR/CD357 (DTA-1, BioLegend, 126312), FITC anti-FOXP3 (FJK-16s, eBioscience, 11-5773-82), PE anti-Granzyme B (QA16A02, BioLegend, 372208), PerCP-Cy5.5 anti-IFNγ (XMG1.2, eBioscience, 45-7311-80), BV421 anti-IFNγ (XMG1.2, BioLegend, 505830), PE-Cy7 anti-KLRG1 (2F1, eBioscience, 25-5893-80), APC anti-KLRG1 (2F1, BD, 561620), PE anti-Perforin (eBioOMAK-D, eBioscience, 12-9392-80), APC anti-Perforin (S16009A, BioLegend, 154304), APC anti-TCRβ (H57-597, eBioscience, 17-5961-82), BV421 anti-TNFα (MP6-XT22, BioLegend, 506328).

***In vivo* suppression assay**

For the *in vivo* suppression assay, naive $CD4^+$ T_{conv} cells ($TCR\beta^+$ $CD4^+$ $CD45RB^+$ $CD25^-$) from CD45.1 mice and T_{reg} cells ($TCR\beta^+$ $CD4^+$ GFP^+) from *Nat10*$^{+/+}$ *Foxp3*$^{GFP\text{-}cre\text{-}ERT2}$ or *Nat10*$^{fl/fl}$ *Foxp3*$^{GFP\text{-}cre\text{-}ERT2}$ mice were sorted. *Rag1*$^{-/-}$ recipients were injected intravenously with 4×10^5 naive $CD4^+$ Tconv cells alone or with 2×10^5 T_{reg} cells. Tamoxifen (1 mg) was administered intraperitoneally at indicated time points. Recipient mice were monitored and weighed every 2-3 days post-transfer.

***Ex vivo* suppression assay**

For the *ex vivo* suppression assay, $CD8^+$ T cells from CD45.1 mice and TI T_{reg} cells

($CD4^+$ GFP^+) from *Nat10*$^{+/+}$ *Foxp3*$^{GFP\text{-}cre\text{-}ERT2}$ or *Nat10*$^{fl/fl}$ *Foxp3*$^{GFP\text{-}cre\text{-}ERT2}$ mice injected intravenously with B16F10 cells were sorted. Enriched $CD8^+$ T cells were labeled with 5 μM CellTrace Violet (Invitrogen, C34557). A total of 1×10^5 $CD8^+$ T cells were cultured alone or with 1×10^5 T_{reg} cells, stimulated with 4 μg/mL anti-CD3e (145-2c11, BD Pharmingen, 553058) and 2 μg/mL anti-CD28 (37.51, BD Pharmingen, 553294) for 72 hours in a 96-well plate.

***In vivo* differentiation assay**

For the *in vivo* iT_{reg} differentiation assay, naive $CD4^+$ T cells ($CD4^+$ $CD25^-$ $CD62L^+$ $CD44^-$) from *Nat10*$^{+/+}$ *Ox40*^{cre} or *Nat10*$^{fl/fl}$ *Ox40*^{cre} mice were sorted. B16F10-bearing CD45.1 mice were injected intravenously with 2×10^6 naive $CD4^+$ T cells. On day 14 post-transfer, the proportion of T_{reg} cells in the tumor was analyzed.

***In vitro* differentiation assay**

For the *in vitro* iT_{reg} differentiation assay, naive $CD4^+$ T cells ($CD4^+$ $CD25^-$ $CD62L^+$ $CD44^-$) from *Nat10*$^{+/+}$ *Ox40*^{cre} or *Nat10*$^{fl/fl}$ *Ox40*^{cre} mice were sorted. Enriched naive $CD4^+$ T cells were labeled with 5 μM CellTrace Violet (Invitrogen, C34557). A total of 5×10^5 naive $CD4^+$ T cells were cultured, stimulated with 2 μg/mL anti-CD3e (145-2c11, BD Pharmingen, 553058), 1 μg/mL anti-CD28 (37.51, BD Pharmingen, 553294), 100 U/mL IL-2 (S10970055), and 2.5 ng/mL TGFβ (UA040172) for 96 hours in a 96-well plate.

In vitro proliferation assay

For the *in vitro* $CD4^+$ T cells proliferation assay, naive $CD4^+$ T cells ($CD4^+$ $CD25^-$ $CD62L^+$ $CD44^-$) from *Nat10*$^{+/+}$ *Ox40*^{cre} or *Nat10*$^{fl/fl}$ *Ox40*^{cre} mice and *Nat10*$^{+/+}$ *Cd4*^{cre} or *Nat10*$^{fl/fl}$ *Cd4*^{cre} mice were sorted. Enriched naive $CD4^+$ T cells were labeled with 5 μM CellTrace Violet (Invitrogen, C34557). A total of 5 × 10^5 naive $CD4^+$ T cells were cultured, stimulated with 2 μg/mL anti-CD3e (145-2c11, BD Pharmingen, 553058) and 1 μg/mL anti-CD28 (37.51, BD Pharmingen, 553294) for 72 hours in a 96-well plate.

For the *in vitro* T_{reg} cells proliferation assay, TI T_{reg} cells ($CD4^+$ $CD25^+$ GFP^+) from *Nat10*$^{+/+}$ *Foxp3*$^{GFP\text{-}cre\text{-}ERT2}$ or *Nat10*$^{fl/fl}$ *Foxp3*$^{GFP\text{-}cre\text{-}ERT2}$ mice injected intravenously with B16F10 cells were sorted. Enriched T_{reg} cells were labeled with 5 μM CellTrace Violet (Invitrogen, C34557). A total of 1 × 10^5 T_{reg} cells were cultured, stimulated with 2 μg/mL anti-CD3e (145-2c11, BD Pharmingen, 553058), 1 μg/mL anti-CD28 (37.51, BD Pharmingen, 553294), 100 U/mL IL-2 (S10970055), and 2 ng/mL TGFβ (UA040172) for 72 hours in a 96-well plate.

Co-transfer assay

For the co-transfer assay, T_{reg} cells from CD45.1/CD45.2 (WT) and CD45.2 (*Nat10*$^{fl/fl}$ *Foxp3*$^{GFP\text{-}cre\text{-}ERT2}$, *Nat10* cKO) mice were sorted. B16F10-bearing CD45.1 mice were injected intravenously with 2 × 10^5 WT and 2 × 10^5 *Nat10* cKO T_{reg} cells. On day 14 post-transfer, the proportion of T_{reg} cells in the tumor was analyzed.

Adoptive transfer and chronic infection

For adoptive transfer, naïve Smarta $CD4^+$ T cells from *Nat10*$^{+/+}$ *Ox40*^{cre} or *Nat10*$^{fl/fl}$ *Ox40*^{cre} Smarta TCR-transgenic mice were harvested. To characterize T cell responses at 30 dpi, 5×10^4 Smarta $CD4^+$ T cells were intravenously injected into CD45.1 recipient mice, followed by intraperitoneal infection with 2×10^6 p.f.u. of LCMV-Cl13.

Luciferase reporter assay

For transactivation analysis, the promoter of TI T_{reg} activation-associated genes (46-bp conserved motif) was cloned into pGL3.basic vector. Renilla vectors were co-transfected into HEK293T cells for normalization. Cells were lysed, and dual luciferase assay (Promega) was performed according to the manufacturer's instructions.

Retroviral constructs and transduction

Mouse WT and mutant *Nat10* cDNAs were cloned into pMigR1-GFP vector (Addgene, #27490). Retrovirus was packaged in HEK293T cells. A total of 5×10^5 naive $CD4^+$ T cells were cultured, stimulated with 2 μg/mL anti-CD3e (145-2c11, BD Pharmingen, 553058), 1 μg/mL anti-CD28 (37.51, BD Pharmingen, 553294), 100 U/mL IL-2 (S10970055), and 2.5 ng/mL TGFβ (UA040172) in a 96-well plate. Cells were infected with retrovirus (2500 rpm, 37 °C, 90 minutes) at 24 hours and 48 hours, and collected at 96 hours for analysis of the GFP^+ T_{reg} cells.

Quantitative PCR with reverse transcription

For gene expression analysis, RNA was extracted using Trizol, quantified and reverse-transcribed into cDNA using HiScript III 1st strand cDNA synthesis kit (Vazyme). RT-qPCR was performed using SYBR qPCR Master Mix (Vazyme). Results were normalized to *Gapdh* and calculated as $2^{-\Delta\Delta Ct}$. Primers used are as follows:

m*Gapdh*-Forward (5’−3’): CATCACTGCCACCCAGAAGACTG

m*Gapdh*-Reverse (5’−3’): ATGCCAGTGAGCTTCCCGTTCAG

m*H2-Ab1*-Forward (5’−3’): CACTCTGGTCTGTTCGGTGAC

m*H2-Ab1*-Reverse (5’−3’): CCTCTCCCTGATGAGGGGTC

m*H2-Eb1*-Forward (5’−3’): GCGGAGAGTTGAGCCTACG

m*H2-Eb1*-Reverse (5’−3’): AGGCCCGTGGACACAATTC

m*H2-K*-Forward (5’−3’): ATACCTGAAGAACGGGAACG

m*H2-K*-Reverse (5’−3’): TGATGTCAGCAGGGTAGAAGC

m*H2-D*-Forward (5’−3’): CGGCTATGTGGACAACAAG

m*H2-D*-Reverse (5’−3’): GTCTCCCGCTCCCAATAC

m*Nat10*-Forward (5’−3’) ATGACCTGCTGTGCCTGGATTG

m*Nat10*-Reverse (5’−3’) GCTGGAGGAAAACTTCAGAGGC

Quantitative PCR with genomic DNA

For qPCR assay of gene enrichment after CUT&Tag, primers used are as follows:

m*Tnfrsf4*-Forward (5’−3’) GTGAAGGCAGAAAGCAGAC

m*Tnfrsf4*-Reverse (5’−3’) CCACTGGGGTAGGTATGT

m*Tnfrsf9*-Forward (5’−3’) CACATGGTTCACAGGGAT

m*Tnfrsf9*-Reverse (5’−3’) AAGTCTCAACCCTTCCGA

m*Tnfrsf18*-Forward (5’−3’) GCTGCTTGGAACACGTTC

m*Tnfrsf18*-Reverse (5’−3’) ACTGGATGGTGGGCAGAGA

m*Pdcd1*-Forward (5’−3’) TCGTACCTGGGAGATGGA

m*Pdcd1*-Reverse (5’−3’) AACCAGAGCTGACCACAGG

m*Cxcr4*-Forward (5’−3’) ATGTTGGAACCTCCGCCA

m*Cxcr4*-Reverse (5’−3’) CCACACCTGACCCTAATG

m*Icos*-Forward (5’−3’) TCTTAGCACCAGATCCTC

m*Icos*-Reverse (5’−3’) GGTTAGCAATGCAGAGTT

Western blot

Whole protein was extracted using RIPA lysis buffer (Beyotime) containing proteinase and phosphatase inhibitors. Nuclear protein and cytoplasmic protein were extracted using Nuclear and Cytoplasmic Extraction Reagents (Thermo Fisher Scientific) and protease inhibitors. Lysates were resolved by SDS-PAGE and transferred to a PVDF membrane (Millipore) for incubation with primary and secondary antibodies. The protein bands were detected using Phototope Horseradish Peroxidase Western Blot Detection kit (Thermo Fisher Scientific). Primary antibodies: NAT10 (1:2,000, ab194297), FOXP3 (1:1,000, ab215206), H3 (1:2,000, ab1791), ZAP70 (1:1,000, ET1706-42), Phospho-ZAP70 (1:1,000, 2717S), AKT (1:1,500, ET1609-47), Phospho-AKT (1:1,500, ET1607-73) and GAPDH (1:100,000, AC033).

Secondary antibodies: Rabbit Anti-mouse IgG (58802S) and Goat Anti-rabbit IgG (7074P2) at 1:10,000.

Co-immunoprecipitation (Co-IP)

Whole protein was extracted using NP-40 lysis buffer (Beyotime) containing proteinase and phosphatase inhibitors. Magnetic beads (MEC) were separated using a magnetic stand, and immunoprecipitated samples were analyzed by immunoblotting with anti-IgG (2729S), anti-NAT10 (ab194297), and anti-FOXP3 (ab215206). The cell lysates were probed with anti-FOXP3 or anti-NAT10 as input.

Immunofluorescence staining

Tumor tissue or cells from humans and mice were sectioned, fixed, and washed. Slides were incubated with BSA and Triton X-100 solution for 1 hour, then incubated overnight at 4 ℃ with primary antibodies: Rabbit anti-NAT10 (1:200, ab194297, abcam), Mouse anti-FOXP3 (1:200, MA5-14662, Invitrogen), Biotin anti-human CD4 (1:200, 300504, BioLegend), Biotin anti-mouse CD4 (1:200, 100508, BioLegend). After washes, slides were incubated with secondary antibodies: Donkey anti-Rabbit 488 (1:200, A-21206, Invitrogen), Donkey anti-Rabbit 647 (1:200, A-31573, Invitrogen), Donkey anti-Mouse 555 (1:200, A-31570, Invitrogen), Mouse Anti-Biotin 647 (1:200, 200-602-211, Jackson), Goat anti-Rat 555 (1:200, A-21434, Invitrogen) for 1 hour. After washes, they were incubated with conjugated antibodies: BV421-labeled anti-EpCAM/CD326 (1:200, 324220, BioLegend), FITC-labeled anti-FOXP3 (1:200,

11-5773-82, eBioscience) for 1 hour. Hoechst staining following by washes, and images were acquired on Zeiss LSM 980.

T_{reg}-specific demethylated region analysis (TSDR)

TI T_{reg} cells ($CD4^+$ GFP^+, 2×10^5) from *Nat10*$^{+/+}$ *Foxp3*$^{GFP\text{-}cre\text{-}ERT2}$ or *Nat10*$^{fl/fl}$ *Foxp3*$^{GFP\text{-}cre\text{-}ERT2}$ mice were sorted for TSDR analysis. DNA was extracted using Ezup Column Animal Genomic DNA Purification Kit (Sangon, B518251) and methylation quantified using EZ DNA Methylation-Gold™ Kit (ZYMO RESEARCH, D5005). Bisulfite treatment converts unmethylated cytosines into uracil, while methylated cytosines remain unchanged. The methylation profile can be determined by PCR and sequencing.

ATAC-seq

TI T_{reg} cells ($CD4^+$ $CD25^+$ GFP^+, 5×10^4) from *Nat10*$^{+/+}$ *Foxp3*$^{GFP\text{-}cre\text{-}ERT2}$ or *Nat10*$^{fl/fl}$ *Foxp3*$^{GFP\text{-}cre\text{-}ERT2}$ mice were sorted for ATAC-seq analysis. DNA was extracted using High-Sensitivity Open Chromatin Profile Kit 2.0 (Novoprotein, N248) and amplified using Index kit for Illumina (Novoprotein, N239). DNA fragments in the range of 200-800 bp were recovered from 2% E-Gel EX Agarose Gels (Invitrogen). The libraries were sequenced on Illumina HiSeq sequencing systems in paired-end 150 bp reads.

CUT&Tag

TI T_{reg} cells ($CD4^+$ $CD25^+$ GFP^+, 1×10^5) from *Nat10*$^{+/+}$ *Foxp3*$^{GFP\text{-}cre\text{-}ERT2}$ or *Nat10*$^{fl/fl}$ *Foxp3*$^{GFP\text{-}cre\text{-}ERT2}$ mice, and T_{conv} cells ($CD4^+$ $CD25^-$, 1×10^5) or iT_{reg} cells (1×10^5) from human peripheral blood were prepared for CUT&Tag analysis. DNA was extracted using Hyperactive Universal CUT&Tag Assay Kit (Vazyme, TD904-01) and amplified using TruePrep Index Kit V2 for Illumina (Vazyme, TD202). Primary antibodies (NAT10 ab194297 at 1:50, H3K27ac ab4729 at 1:50, and FOXP3 14-4774-82 at 1:25) and secondary antibodies (anti-Rabbit Ab207-01 at 1:100 or anti-Mouse Ab208-01 at 1:100) were used. The libraries were paired-end sequencing on Illumina NovaSeq platform.

Whole-genome bisulfite sequencing (WGBS)

TI T_{reg} cells ($CD4^+$ $CD25^+$ GFP^+, 1×10^5) from *Nat10*$^{+/+}$ *Foxp3*$^{GFP\text{-}cre\text{-}ERT2}$ or *Nat10*$^{fl/fl}$ *Foxp3*$^{GFP\text{-}cre\text{-}ERT2}$ mice were sorted for WGBS analysis. Cells were lysed in 50 µL lysis buffer (25.5 µL 2× M-Digestion Buffer, 5.1 µL Proteinase K at 20 mg/mL, and 19.4 µL nuclease-free H_2O) at 50°C for 1 hour. The lysates were then fragmented to an average DNA size of ~550 bp using a Covaris S220 focused ultrasonicator. Fragmented DNA was subjected to bisulfite conversion using the Hieff® Mag Superfast DNA Methylation Bisulfite Kit (Yeasen, 12229). WGBS libraries were prepared from bisulfite-converted DNA using the EpiArt® DNA Methylation Library Kit for Illumina V3 (Vazyme, NE103). Adapter-ligated DNA was amplified, purified, and subjected to sequencing on an Illumina platform.

ac4C RIP-seq

TI T_{reg} cells ($CD4^+$ $CD25^+$ GFP^+, 1×10^6) from *Nat10*$^{+/+}$ *Foxp3*$^{GFP\text{-}cre\text{-}ERT2}$ or *Nat10*$^{fl/fl}$ *Foxp3*$^{GFP\text{-}cre\text{-}ERT2}$ mice were sorted for ac4C RIP analysis. RNA was extracted using RNeasy UCP Micro Kit (Qiagen). The ac4C-RIP-seq service was provided by CloudSeq (Shanghai, China).

RNA-seq

TI T_{reg} cells ($CD4^+$ $CD25^+$ GFP^+, 1×10^5) from *Nat10*$^{+/+}$ *Foxp3*$^{GFP\text{-}cre\text{-}ERT2}$ or *Nat10*$^{fl/fl}$ *Foxp3*$^{GFP\text{-}cre\text{-}ERT2}$ mice were sorted for RNA-seq analysis. RNA was extracted using RNeasy UCP Micro Kit (Qiagen). The RNA-seq service was provided by Novogene (Tianjin, China).

High-resolution chromosome-conformation capture (Hi-C)

TI T_{reg} cells ($CD4^+$ $CD25^+$ GFP^+, 1×10^6) from *Nat10*$^{+/+}$ *Foxp3*$^{GFP\text{-}cre\text{-}ERT2}$ or *Nat10*$^{fl/fl}$ *Foxp3*$^{GFP\text{-}cre\text{-}ERT2}$ mice were sorted for Hi-C analysis. Cells were crosslinked with 1% formaldehyde (25 °C, 10 min), lysed in buffer (10 mM Tris-HCl pH 8.0, 10 mM NaCl, 0.2% NP-40, protease inhibitors) at 4 °C for 15 min to collect nuclei. Nuclei were treated with 0.5% SDS (62 °C, 10 min), quenched with Triton X-100, and digested with NUB I (37 °C, overnight), followed by heat inactivation (65 °C, 20 min). DNA ends were blunted and biotin-labeled using Klenow with biotin-14-dATP and dNTPs, then ligated with T4 ligase. After reverse crosslinking and sonicated, biotinylated fragments were captured with streptavidin magnetic beads. Libraries were constructed

via end-repair, adaptor ligation, and PCR; 200-500 bp fragments were gel-purified and sequenced on Illumina HiSeq in paired-end 150 bp mode.

Protein expression and purification

Plasmid encoding *Nat10* was purchased from Tsingke Biotechnology. The *Nat10* gene was ligated into pET-22b vector between *Nde*I and *Xho*I. *Nat10*$_{\mathrm{H537A/Y538A}}$ and *Nat10*$_{\mathrm{exon4\text{-}5\ deletion}}$ mutation were introduced by PCR and assembled into the linearized pET-22b vector using ClonExpress II One Step Cloning Kit (Vazyme). The *Nat10* or mutants were transformed into Escherichia coli BL21 (DE3) to produce the target protein. E. coli cells were cultured in LB medium with 50 mg/L kanamycin at 37 °C until the OD600 reached 0.6-0.8, then the bacteria were induced with 0.2 mM isopropyl-β-d-thiogalactoside (IPTG) and incubated at 16 °C for 18 hours. Bacteria were collected by centrifugation, resuspended in buffer (20 mM Tris-HCl, pH 7.0, 500 mM NaCl, and 25 mM imidazole, pH 7.0), and lysed by high-pressure treatment. Cell extracts were centrifuged at 4 °C, 13,000 rpm for 1 hour. Supernatants were purified with Ni-NTA, and the target protein was washed with lysis buffer and then eluted with elution buffer (20 mM Tris-HCl, pH 7.0, 500 mM NaCl, and 500 mM imidazole). Eluted proteins were concentrated by centrifugal ultrafiltration, loaded onto a pre-equilibrated Superdex 200 Increase 10/300 GL column, and eluted at a flow rate of 0.3 mL/min with buffer (20 mM Tris-HCl, pH 7.0, 250 mM NaCl). Peak fractions were analyzed by SDS-PAGE (15%, w/v) and stained with Coomassie Brilliant Blue R-250. Purified fractions were pooled, concentrated and quantified by A_{280}.

EMSA

An aliquot of 100 nM DNA was mixed with increasing protein concentrations from 0 to 40 times the DNA concentration with eight gradients in 10 μL buffer (20 mM HEPES, pH 7.0, 250 mM NaCl) at 4 °C for 3 hours. Electrophoresis was performed with 6% native PAGE gels in 1 × Tris-borate-EDTA (TBE) buffer at 4 °C. Gels were scanned using a Typhoon FLA-9000 (GE Healthcare).

***In vitro* acetyltransferase activity assay**

Acetyltransferase activity was measured in a 30 μL reaction volume containing 50 mM HEPES-Na, pH 7.5, 50 mM NaCl, 5 mM $MgCl_2$, 1 mM DTT, 1 mM ATP, 0.5 μM recombinant protein, 1 μM RNA substrate, and 1 μL of 0.1 μCi/μL 3H-labeled Acetyl-CoA (Acetyl Coenzyme A, [Acetyl-3H]; PerkinElmer, USA). The reaction products were extracted using RNA purification and concentration kit (TR115; Genstone Biotech, CN) and transferred into scintillation vials containing 5 mL of scintillation fluid (Ultima Gold™, PerkinElmer). Radioactivity was measured using a liquid scintillation counter (Beckman Coulter, LS6500, USA) to quantify the 3H-acetylated products.

Bioinformatic analysis

For dataset GSE99254, quality control was performed to filter out low-quality cells (low total read counts, high mitochondrial gene expression, or insufficient transcript coverage). Dimensionality reduction was used to visualize overall structure,

and k-means clustering was applied to identify cell subpopulations based on gene expression profiles. The results were visualized using heatmaps, violin plots, and scatter plots. Scanpy was used for clustering and visualization.

For dataset GSE235500, quality control excluded low-quality cells with 200 detected genes or more than 10% mitochondrial gene content. The data were normalized, log-transformed, highly variable genes were identified, and scaled. Batch effects were corrected using Harmony (v0.1.1). Dimensionality reduction was performed via principal component analysis (PCA), followed by visualization with Uniform Manifold Approximation and Projection (UMAP). Clustering was conducted using the Leiden algorithm at a resolution of 0.5. Cell type annotation was based on the expression of canonical marker genes and cross-referenced with publicly available single-cell atlases. Differentially expressed genes (DEGs) were identified using the Wilcoxon rank-sum test with Benjamini-Hochberg correction for multiple testing. All visualizations were generated using Scanpy's built-in plotting functions or customized with Matplotlib and Seaborn.

Supplemental Figure 1

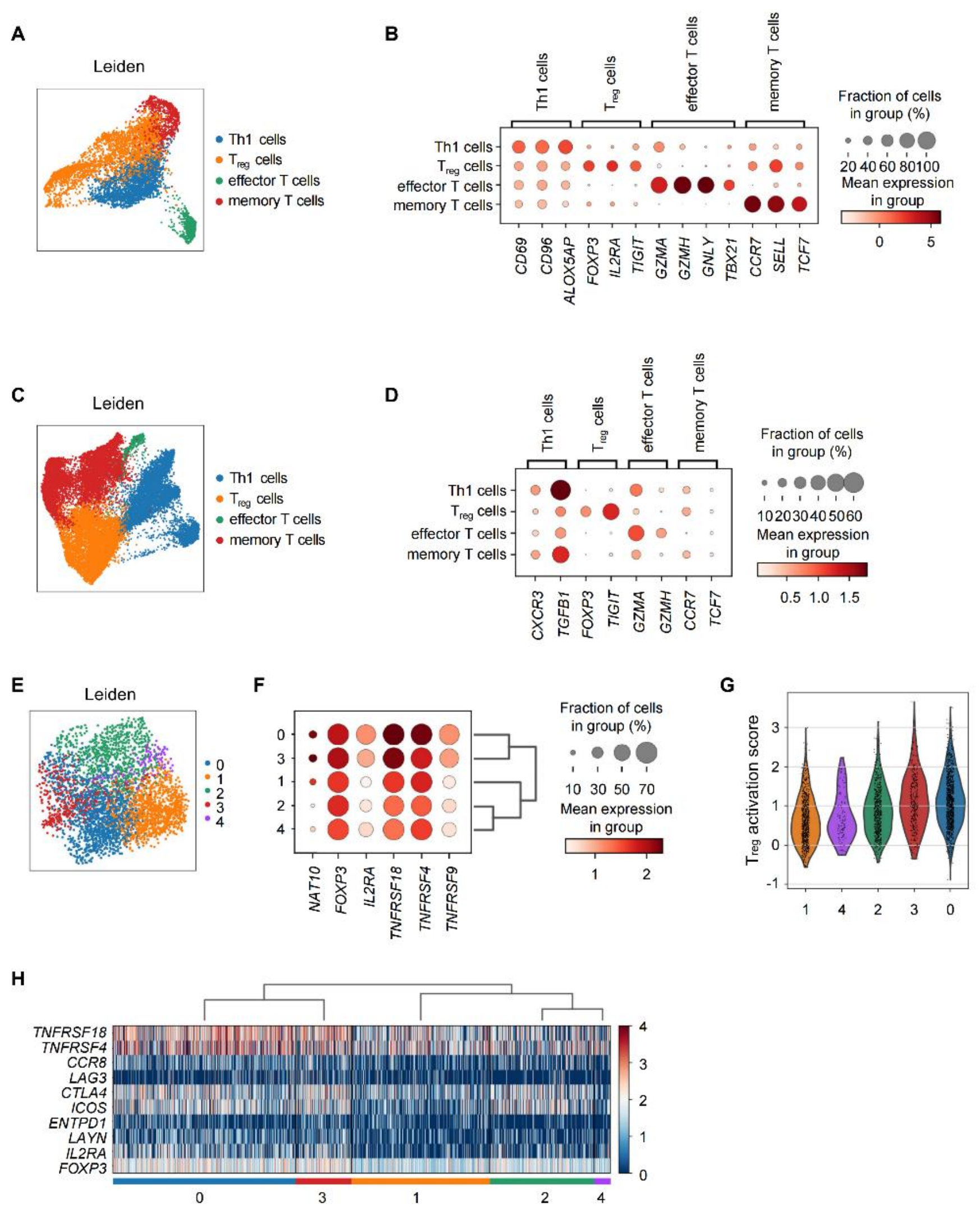

Supplemental Figure 1. Increased *NAT10* expression in T_{reg} cells of human lung cancers.

(A) UMAP representation of the $CD4^+$ T cells (5,547 cells) depicted in different colors by cell types in the GSE99254 dataset (11,769 cells total). **(B)** Dot plot displaying the expression of marker genes of specific $CD4^+$ T cell clusters in the GSE99254 dataset. **(C)** UMAP representation of the $CD4^+$ T cells (22,079 cells) depicted in different colors by cell types in the GSE235500 dataset (144,330 cells total). **(D)** Dot plot displaying the expression of marker genes of specific $CD4^+$ T cell clusters in the GSE235500

dataset. **(E)** UMAP representation of the cells depicted in different colors by subclusters of *FOXP3* positive T_{reg} cells (2,989 cells) in the GSE235500 dataset. **(F)** Dot plot displaying the expression of *NAT10*, *FOXP3*, *IL2RA*, *TNFRSF18*, *TNFRSF4*, and *TNFRSF9* in different subclusters of T_{reg} cells in the GSE235500 dataset. **(G)** T_{reg} activation scores were calculated by the expression pattern of T_{reg} cell signature genes. **(H)** Heat map showing the expression of signature genes used to calculate the T_{reg} activation scores.

Supplemental Figure 2

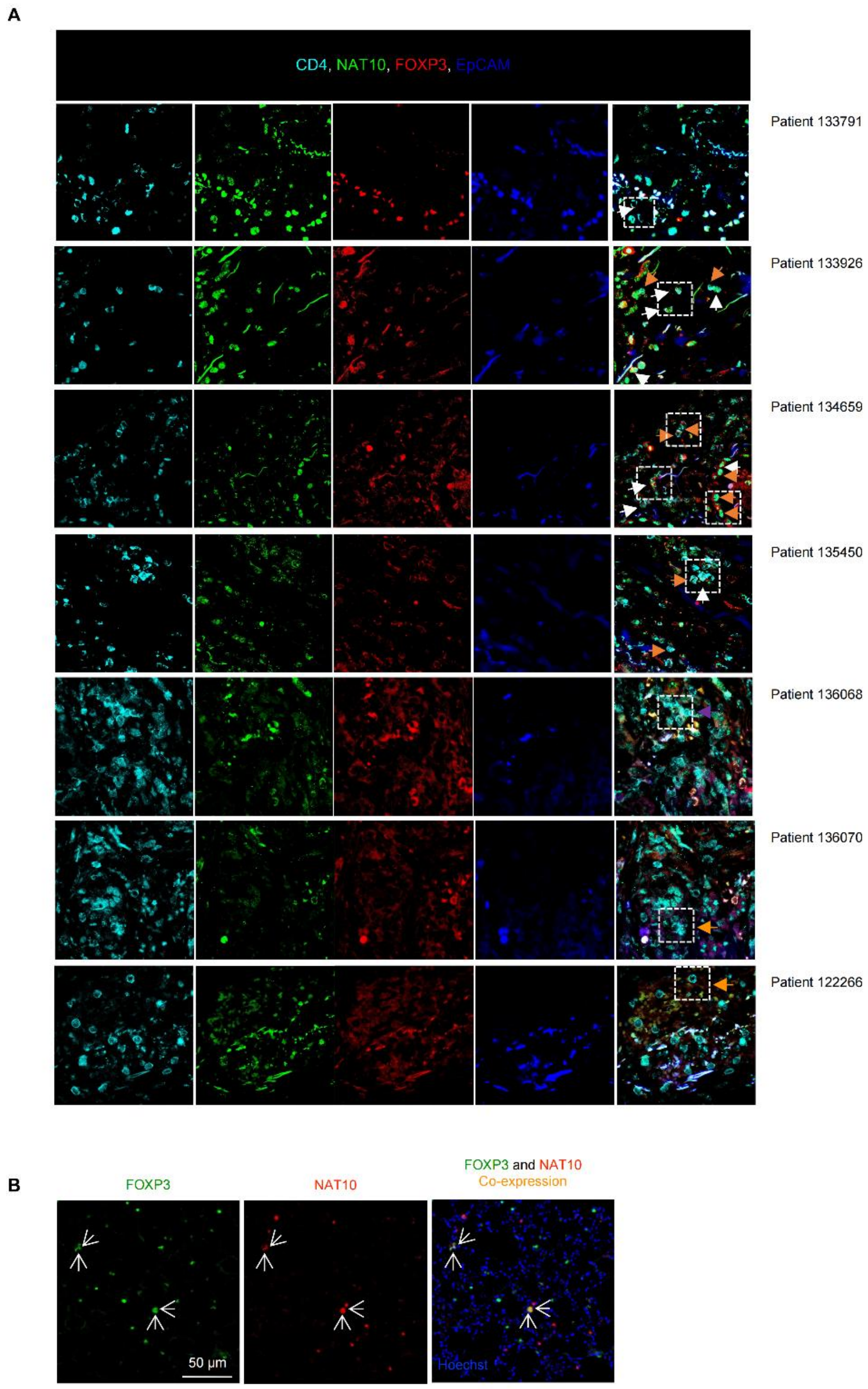

Supplemental Figure 2. Immunofluorescence staining shows NAT10$^+$ T$_{reg}$ cells in cancer tissues.

(A) Immunofluorescence staining of lung tissues from human NSCLC. **(B)** Immunofluorescence staining of lung tissues from mice intravenously injected with B16F10 cells.

Supplemental Figure 3

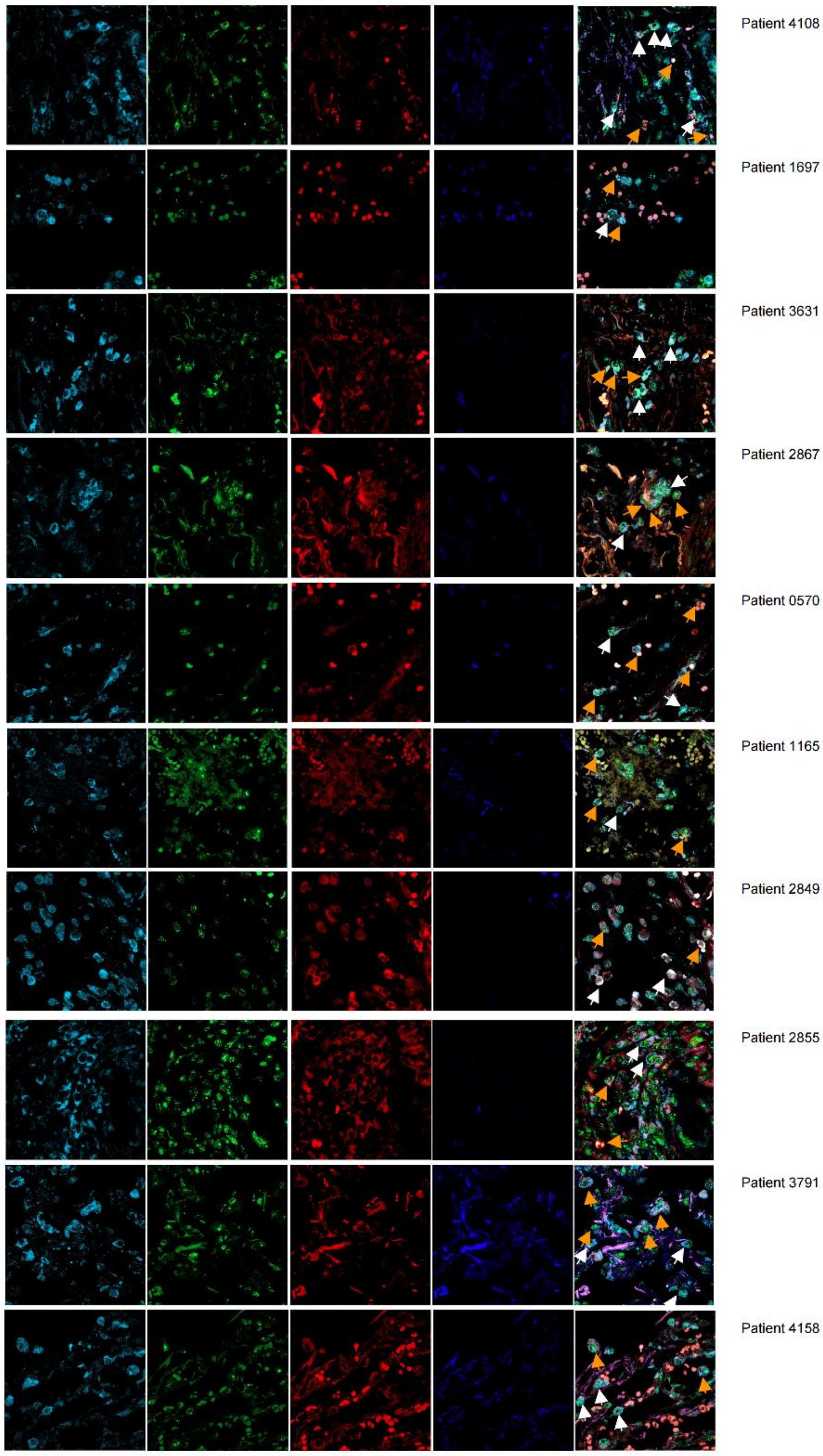

Supplemental Figure 3. Immunofluorescence staining shows NAT10^{+} T$_{reg}$ cells in cancer tissues.

Immunofluorescence staining of lung tissues from human NSCLC.

Supplemental Figure 4

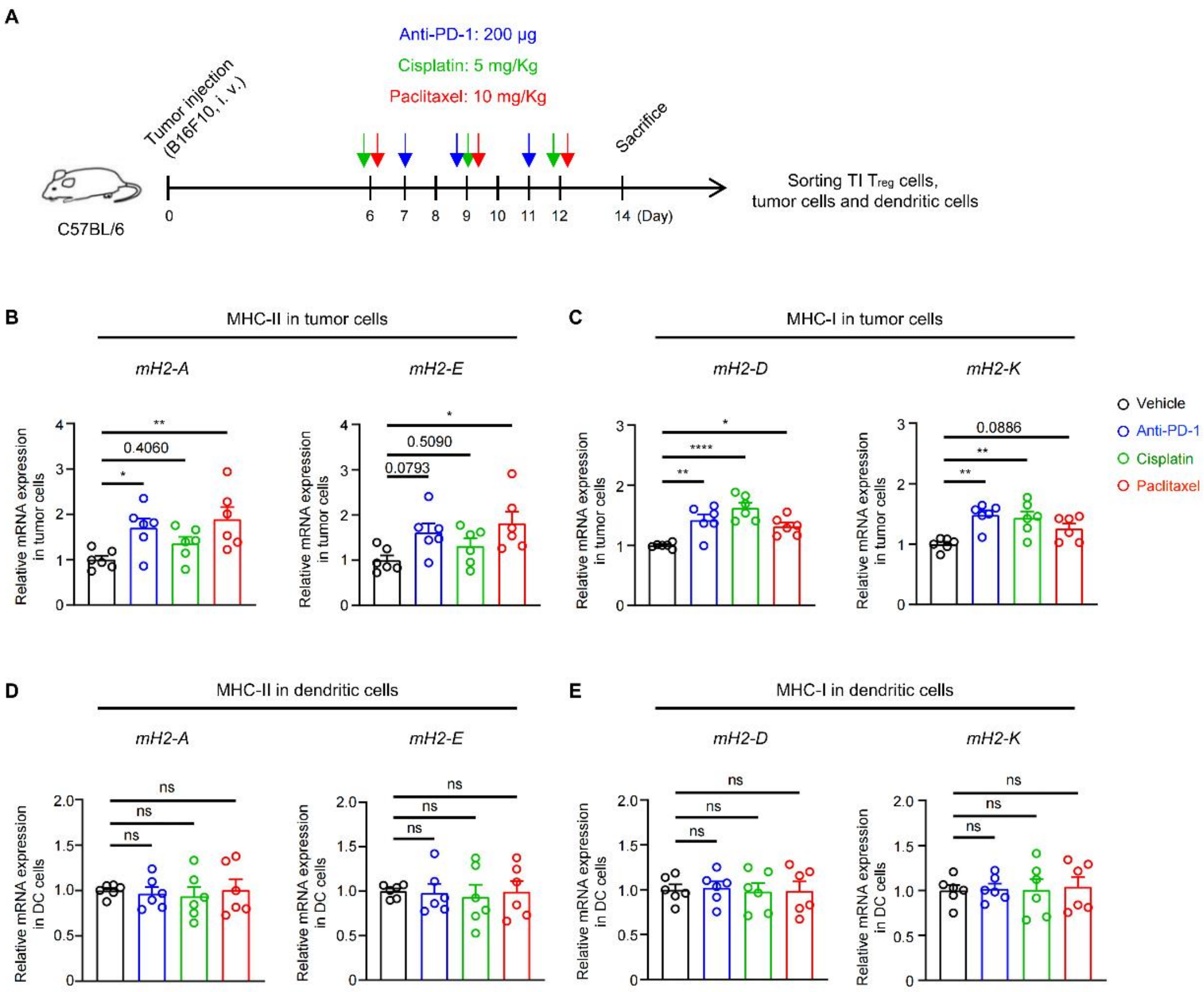

Supplemental Figure 4. Anti-PD-1 immunotherapy and chemotherapy induce MHC-II molecules expression of B16F10 tumor cells.

(A) Schematic of the experiment. **(B and C)** Real-time PCR showing relative mRNA expression of MHC-II (**B**) and MHC-I (**C**) in tumor cells after anti-PD-1, Cisplatin, or Paclitaxel treatment (n = 6). **(D and E)** Real-time PCR showing relative mRNA expression of MHC-II (**D**) and MHC-I (**E**) in dendritic cells after anti-PD-1, Cisplatin, or Paclitaxel treatment (n = 6). Data represent 2-3 independent experiments, mean ± SEM. P values were calculated using one-way ANOVA (**B-E**).

Supplemental Figure 5

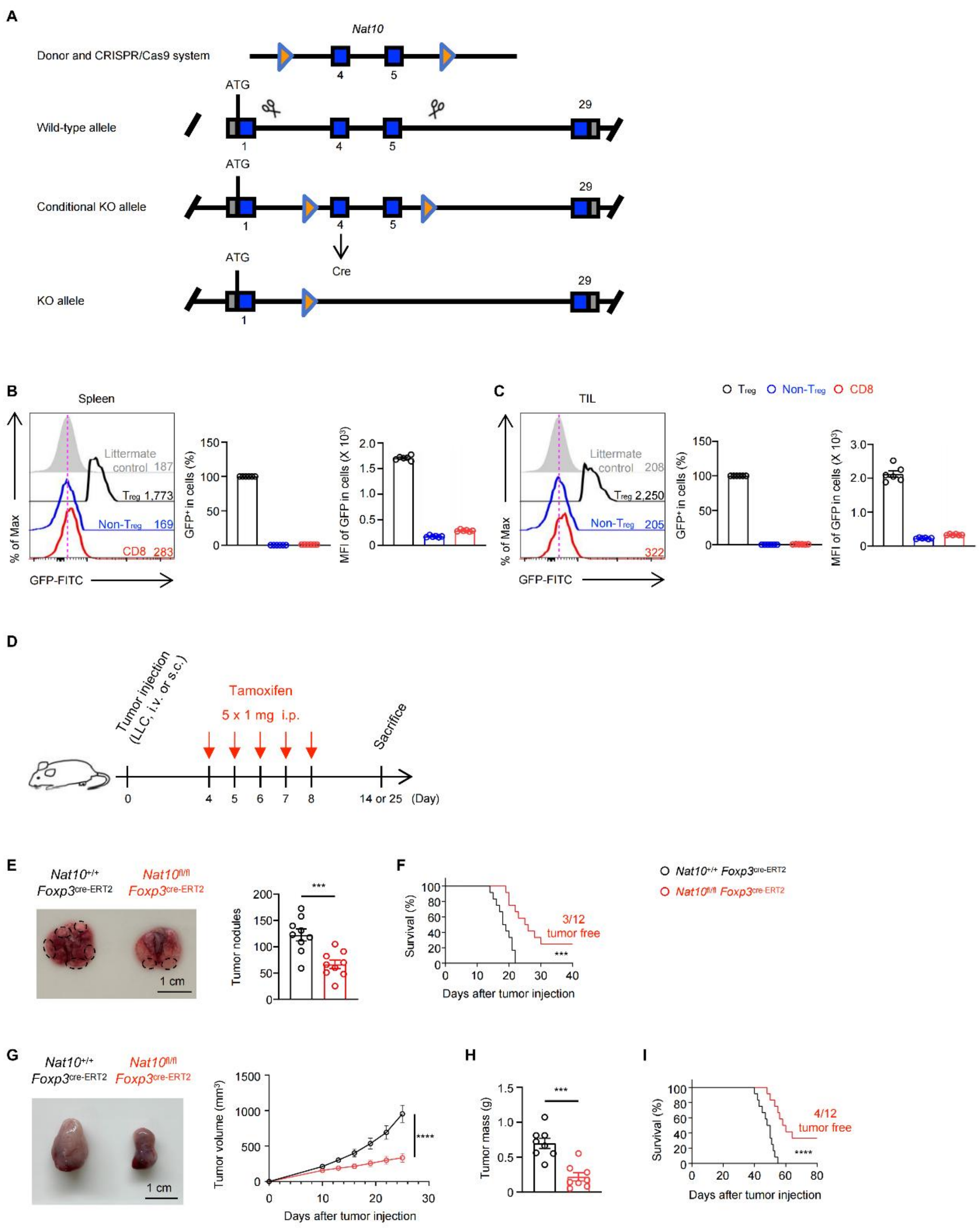

Supplemental Figure 5. Mice with *Nat10* genetic ablation in TI T_{reg} cell limit tumor progression.

(A) Establishment of the *Nat10*^{flox} mice using CRISPR/Cas9. **(B and C)** Representative histograms (left), quantification of the percentages (middle) and MFI (right) of GFP

(FOXP3) in $CD4^+$ GFP^+ T_{reg} cells, $CD4^+$ GFP^- non-T_{reg} cells and $CD8^+$ T cells from the spleen **(B)** and TIL **(C)** of *Foxp3*$^{cre\text{-}ERT2}$ mice intravenously injected with B16F10 cells (n = 6). **(D)** Schematic of the experiment. **(E)** Quantification of tumor nodules in the lungs of *Nat10*$^{+/+}$ *Foxp3*$^{cre\text{-}ERT2}$ and *Nat10*$^{fl/fl}$ *Foxp3*$^{cre\text{-}ERT2}$ mice at day 14 post-intravenous injection with LLC cells (n = 9). **(F)** Survival curve of *Nat10*$^{+/+}$ *Foxp3*$^{cre\text{-}ERT2}$ and *Nat10*$^{fl/fl}$ *Foxp3*$^{cre\text{-}ERT2}$ mice intravenously injected with LLC cells (n = 12). **(G and H)** Tumor growth rate **(G)** and tumor mass **(H)** in *Nat10*$^{+/+}$ *Foxp3*$^{cre\text{-}ERT2}$ and *Nat10*$^{fl/fl}$ *Foxp3*$^{cre\text{-}ERT2}$ mice at day 25 post-subcutaneous injection with LLC cells (n = 8). **(I)** Survival curve of *Nat10*$^{+/+}$ *Foxp3*$^{cre\text{-}ERT2}$ and *Nat10*$^{fl/fl}$ *Foxp3*$^{cre\text{-}ERT2}$ mice subcutaneously injected with LLC cells (n = 12). Data represent 2-3 independent experiments, mean ± SEM. P values were calculated using two-tailed unpaired Student's *t*-test **(E, H),** two-way ANOVA **(G)** and log-rank test **(F, I)**.

Supplemental Figure 6

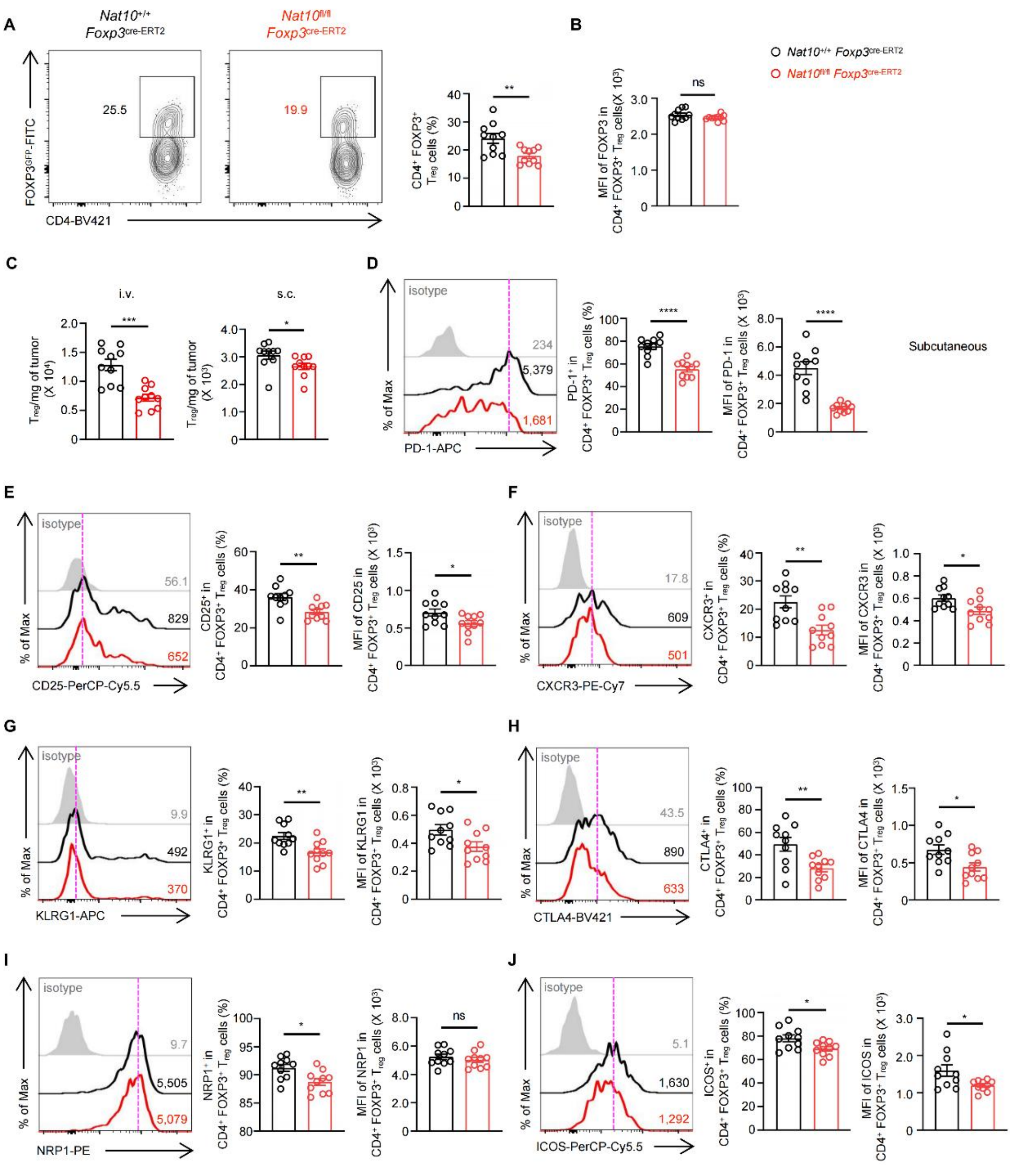

Supplemental Figure 6. ***Nat10*** **ablation in TI T_{reg} cell reduces phenotypic markers associated with T_{reg} cell function.**

(A and B) Representative flow cytometry plots (left) and quantification of the percentage (right) of TI T_{reg} cells **(A)**, and MFI of FOXP3 **(B)** in *Nat10*$^{+/+}$ *Foxp3*$^{\text{cre-ERT2}}$ and *Nat10*$^{\text{fl/fl}}$ *Foxp3*$^{\text{cre-ERT2}}$ mice subcutaneously injected with B16F10 cells (n = 10). **(C)** The number of TI T_{reg} cells, relative to tumor weight (n = 10). **(D-J)** Representative

histograms (left), quantification of the percentages (middle) and MFI (right) of PD-1 **(D)**, CD25 **(E)**, CXCR3 **(F)**, KLRG1 **(G)**, CTLA4 **(H)**, NRP1 **(I)**, and ICOS **(J)** in TI T_{reg} cells from *Nat10*$^{+/+}$ *Foxp3*$^{cre\text{-}ERT2}$ and *Nat10*$^{fl/fl}$ *Foxp3*$^{cre\text{-}ERT2}$ mice subcutaneously injected with B16F10 cells (n = 10). Data represent 2-3 independent experiments, mean ± SEM. P values were calculated using two-tailed unpaired Student's *t*-test **(A-J)**.

Supplemental Figure 7

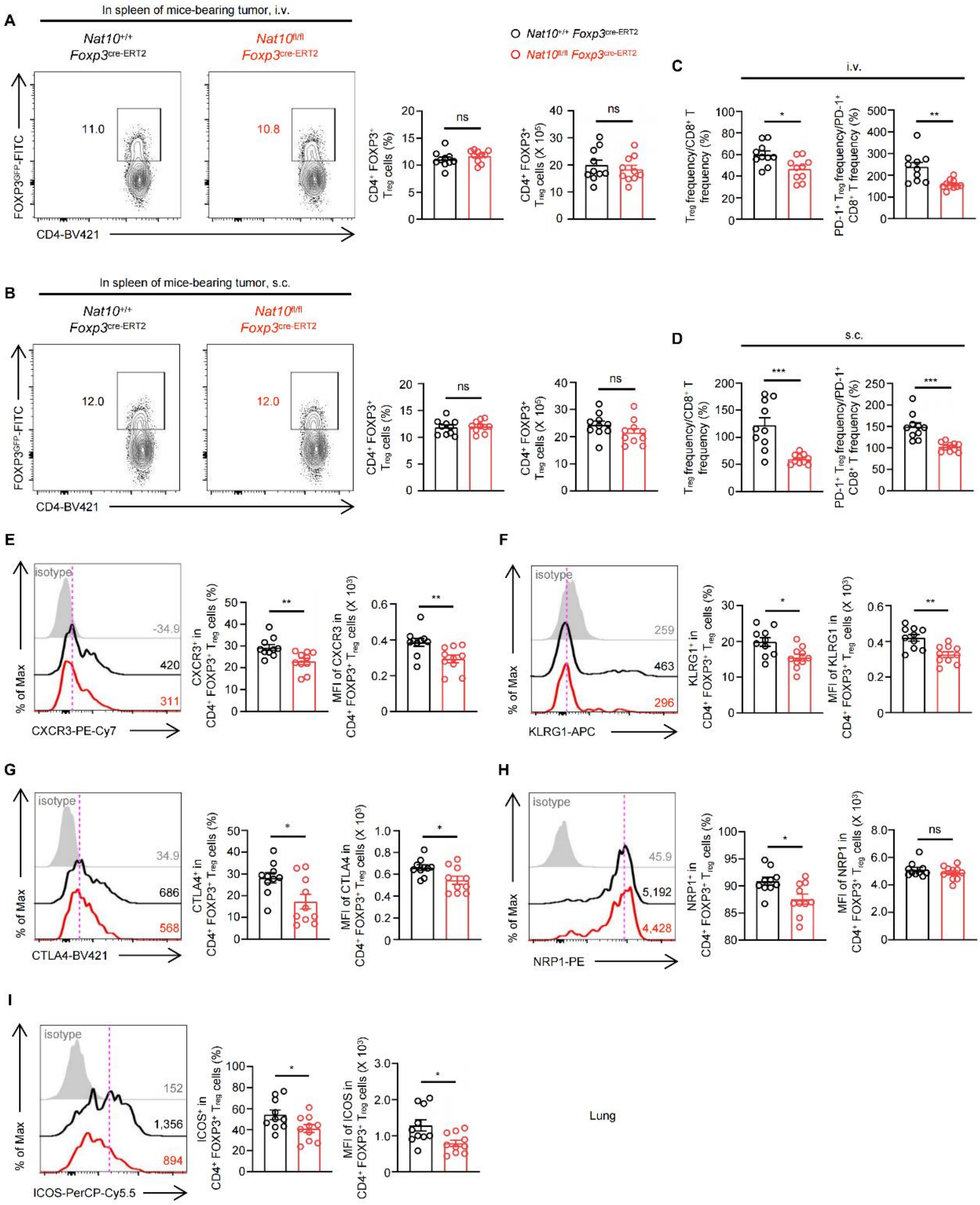

Supplemental Figure 7. ***Nat10*** **ablation in TI T_{reg} cells does not affect peripheral spleen T_{reg} cells.**

(A) Representative flow cytometry plots (left), quantification of the percentages (middle) and number (right) of spleen T_{reg} cells in the lung tumor model (n = 10). **(B)** Representative flow cytometry plots (left), quantification of the percentages (middle)

and number (right) of spleen T_{reg} cells in the subcutaneous tumor model (n = 10). **(C)** Ratio of T_{reg} cells to $CD8^+$ T cells (left) and PD-1^+ T_{reg} cells to $CD8^+$ T cells (right) in the lung tumor model (n = 10). **(D)** Ratio of T_{reg} cells to $CD8^+$ T cells (left) and PD-1^+ T_{reg} cells to $CD8^+$ T cells (right) in the subcutaneous tumor model (n = 10). **(E-I)** Representative histograms (left), quantification of the percentages (middle) and MFI (right) of CXCR3 **(E)**, KLRG1 **(F)**, CTLA4 **(G)**, NRP1 **(H)**, and ICOS **(I)** in TI T_{reg} cells from *Nat10*$^{+/+}$ *Foxp3*$^{\text{cre-ERT2}}$ and *Nat10*$^{\text{fl/fl}}$ *Foxp3*$^{\text{cre-ERT2}}$ mice intravenously injected with B16F10 cells (n = 10). Data represent 2-3 independent experiments, mean ± SEM. P values were calculated using two-tailed unpaired Student's *t*-test **(A-I)**.

Supplemental Figure 8

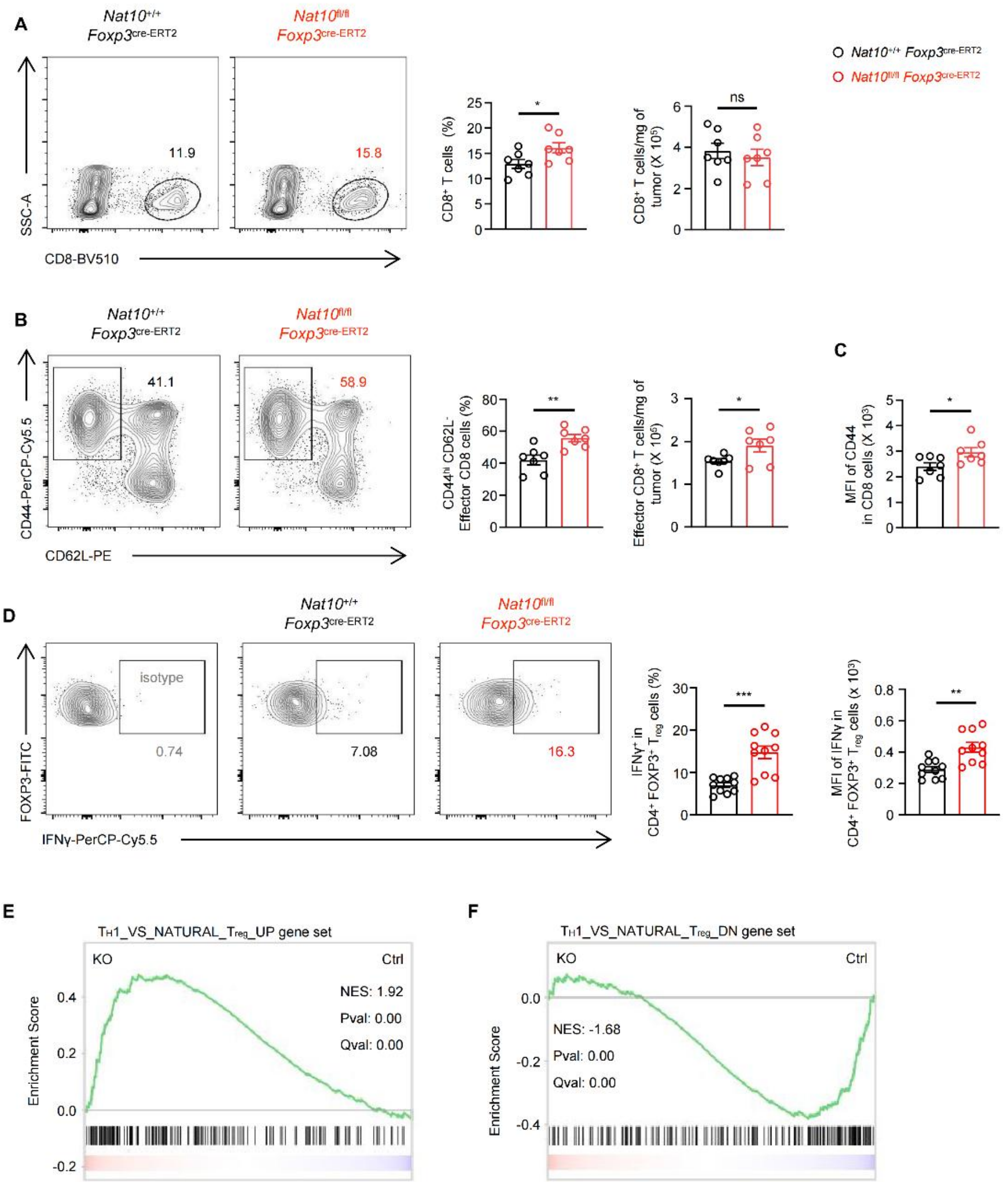

Supplemental Figure 8. In mice with *Nat10*-deficient T_{reg} cells, TI $CD8^+$ T cells significantly increase and exhibit the effector state.

(A) Representative flow cytometry plots (left), quantification of the percentages (middle) and number (right) of TI $CD8^+$ T cells in the lung tumor model (n = 7). **(B and C)** Representative flow cytometry plots (left), quantification of the percentages (middle) and number (right) of effector $CD8^+$ T cells **(B)**, and MFI of CD44 **(C)** in the lung tumor model (n = 7). **(D)** Representative flow cytometry plots (left), quantification of the

percentages (middle) and MFI (right) of IFNγ in TI T_{reg} cells after *ex vivo* stimulation with anti-CD3 and anti-CD28 from *Nat10*$^{+/+}$ *Foxp3*$^{cre\text{-}ERT2}$ and *Nat10*$^{fl/fl}$ *Foxp3*$^{cre\text{-}ERT2}$ mice intravenously injected with B16F10 cells (n = 10). **(E and F)** GSEA analysis shows the enrichment of T_H1 vs T_{reg} cell-associated upregulated **(E)** and downregulated **(F)** genes in the transcriptome of *Nat10*-deficient (KO) vs Ctrl T_{reg} cells. Data represent 2-3 independent experiments, mean ± SEM. P values were calculated using two-tailed unpaired Student's *t*-test **(A-D)**.

Supplemental Figure 9

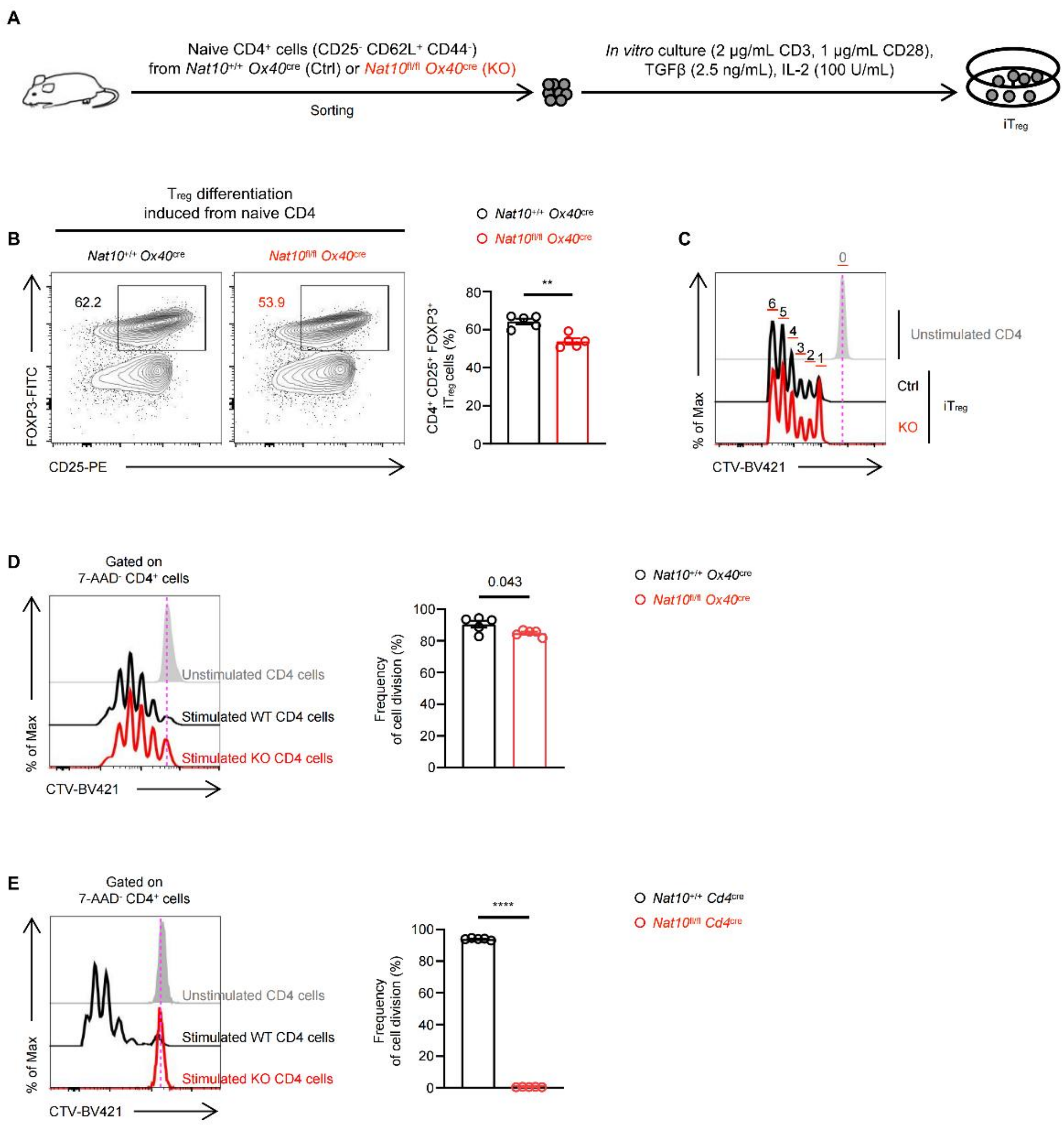

Supplemental Figure 9. NAT10 promotes iT$_{reg}$ cell differentiation.

(A) Schematic of the experiment. **(B)** Representative flow cytometry plots (left), quantification of the percentages (right) of induced iT$_{reg}$ cells from the activated CD4$^+$ T cells of *Nat10*$^{+/+}$ *Ox40*^{cre} and *Nat10*$^{fl/fl}$ *Ox40*^{cre} mice (n = 5). **(C)** Representative histogram of iT$_{reg}$ cells division from the activated CD4$^+$ T cells of *Nat10*$^{+/+}$ *Ox40*^{cre} and *Nat10*$^{fl/fl}$ *Ox40*^{cre} mice. **(D)** Representative histogram (left) and quantification of the percentages (right) of CD4$^+$ T cells division from the naïve CD4$^+$ T cells of *Nat10*$^{+/+}$ *Ox40*^{cre} and *Nat10*$^{fl/fl}$ *Ox40*^{cre} mice (n = 5). **(E)** Representative histogram (left) and

quantification of the percentages (right) of $CD4^+$ T cells division from the naïve $CD4^+$ T cells of *Nat10*$^{+/+}$ *Cd4*^{cre} and *Nat10*$^{fl/fl}$ *Cd4*^{cre} mice (n = 5). Data represent 2-3 independent experiments, mean ± SEM. P values were calculated using two-tailed unpaired Student's *t*-test **(B, D, E)**.

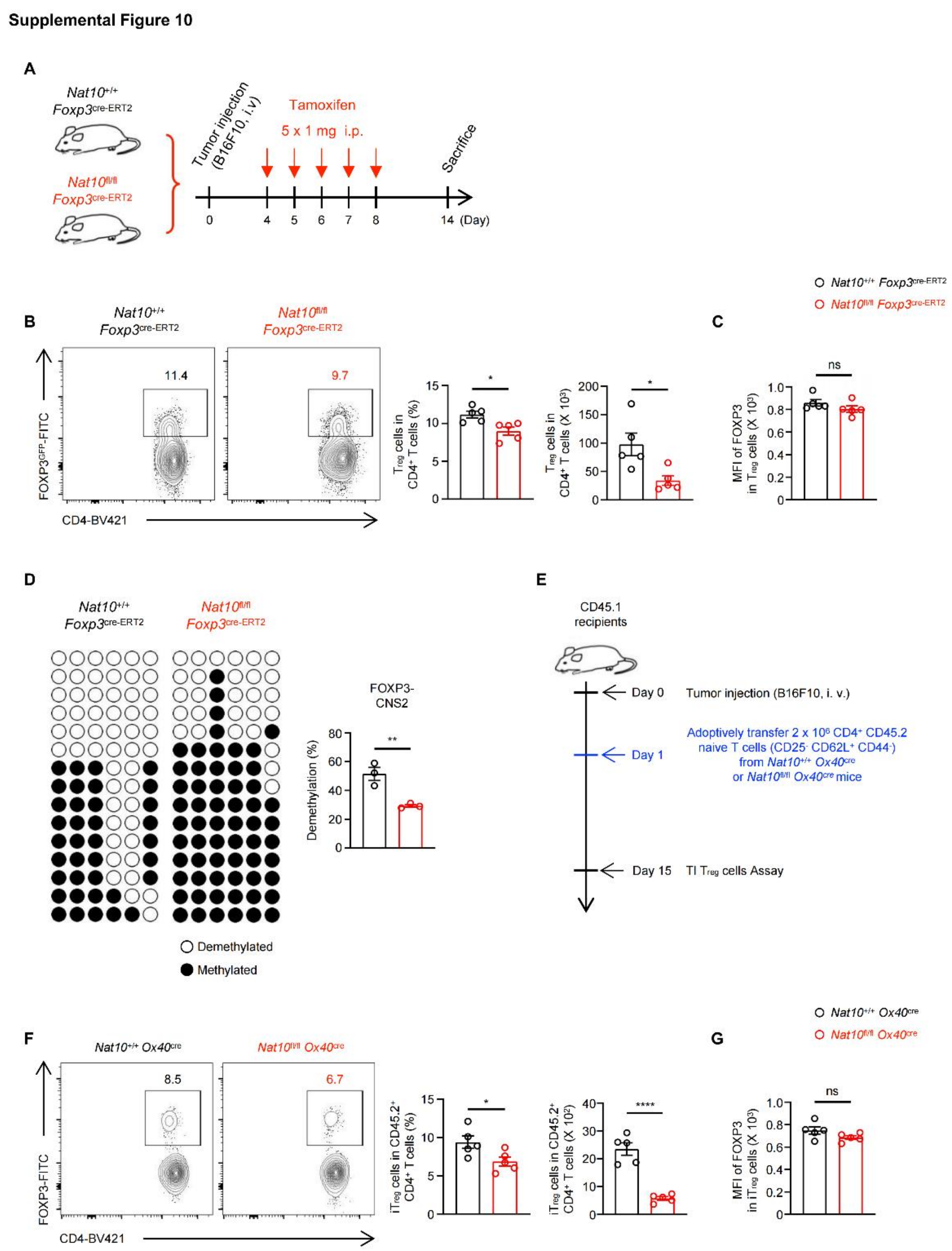

Supplemental Figure 10. ***Nat10*** **ablation impairs iT$_{reg}$ cell differentiated from CD4^{+} T cells.**

(A) Schematic of the experiment. **(B and C)** Representative flow cytometry plots (left), quantification of the percentage (middle) and number (right) of T$_{reg}$ cells **(B)**, and MFI of FOXP3 **(C)** in *Nat10*$^{+/+}$ *Foxp3*$^{cre\text{-}ERT2}$ and *Nat10*$^{fl/fl}$ *Foxp3*$^{cre\text{-}ERT2}$ mice intravenously injected with B16F10 cells (n = 5). **(D)** TSDR comparative analysis showing the

frequency of demethylation in TI T_{reg} cells (n = 3). **(E)** Schematic of the experiment. **(F and G)** Representative flow cytometry plots (left), quantification of the percentage (middle) and number (right) of iT_{reg} cells **(F)**, and MFI of FOXP3 **(G)** in CD45.1 mice intravenously injected with B16F10 cells and naïve $CD4^+$ T cells (n = 5). Data represent 2-3 independent experiments, mean ± SEM. P values were calculated using two-tailed unpaired Student's *t*-test **(B-D, F, G)**.

Supplemental Figure 11

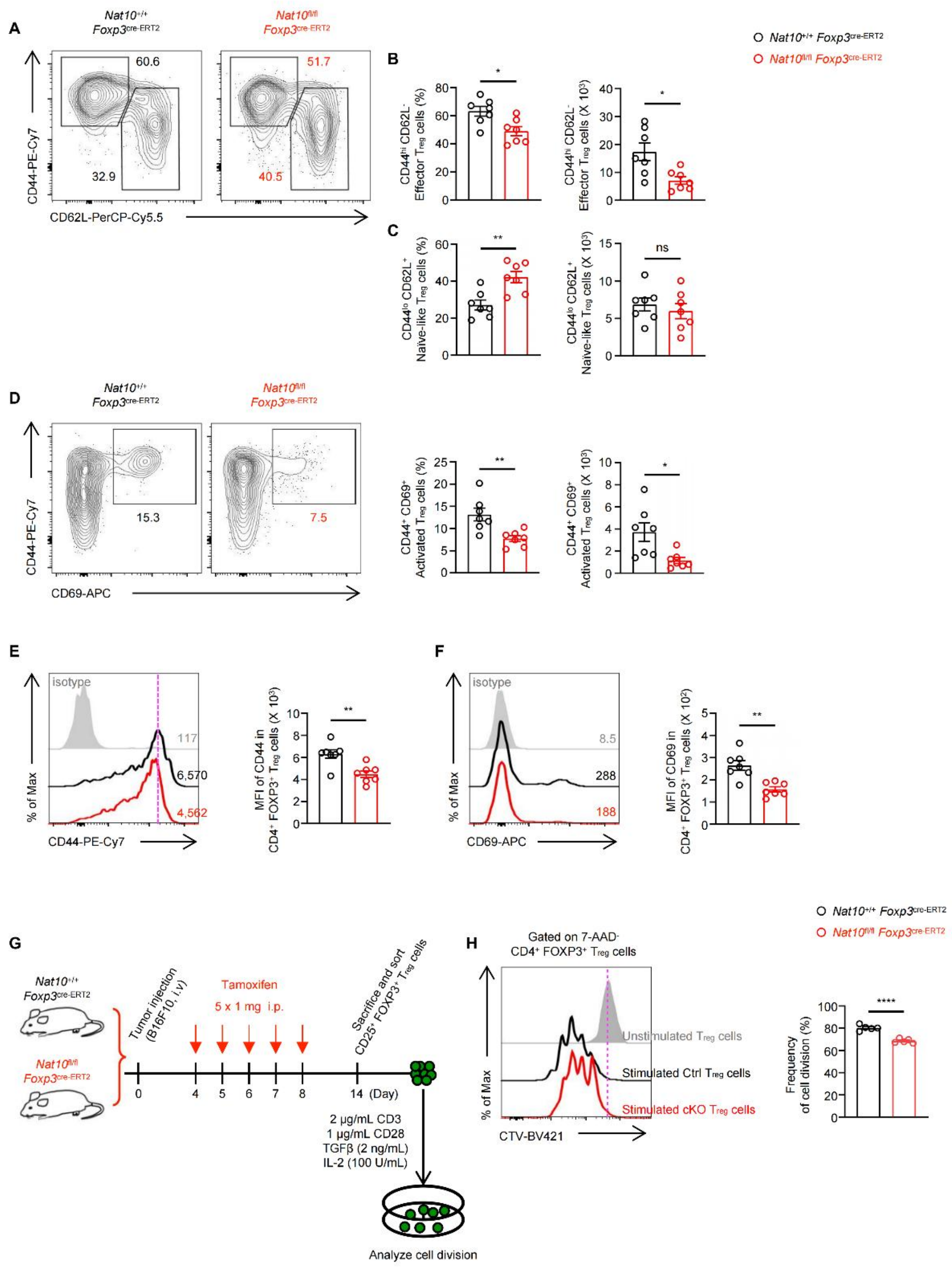

Supplemental Figure 11. ***Nat10*** **ablation leads to TI T$_{reg}$ cells located at the inactivated or naïve-like state.**

(A-C) Representative flow cytometry plots **(A),** quantification of the percentages (left) and number (right) of CD44^{hi} CD62L^{-} effector T$_{reg}$ cells (**B**) and CD44^{lo} CD62L^{+} naïve-like T$_{reg}$ cells (**C**) from *Nat10*$^{+/+}$ *Foxp3*$^{cre\text{-}ERT2}$ and *Nat10*$^{fl/fl}$ *Foxp3*$^{cre\text{-}ERT2}$ mice

intravenously injected with B16F10 cells (n = 7). **(D-F)** Representative flow cytometry plots (left), quantification of the percentages (middle) and number (right) of CD44^{+} CD69^{+} activated T_{reg} cells **(D)**, and MFI of CD44 **(E)** and CD69 **(F)** from *Nat10*$^{+/+}$ *Foxp3*$^{cre\text{-}ERT2}$ and *Nat10*$^{fl/fl}$ *Foxp3*$^{cre\text{-}ERT2}$ mice intravenously injected with B16F10 cells (n = 7). **(G)** Schematic of the experiment. **(H)** Representative histogram (left) and quantification of the percentages (right) of T_{reg} cells division from the TI T_{reg} cells of *Nat10*$^{+/+}$ *Foxp3*$^{cre\text{-}ERT2}$ and *Nat10*$^{fl/fl}$ *Foxp3*$^{cre\text{-}ERT2}$ mice intravenously injected with B16F10 cells (n = 5). Data represent 2-3 independent experiments, mean ± SEM. P values were calculated using two-tailed unpaired Student's *t*-test **(B-F, H)**.

Supplemental Figure 12

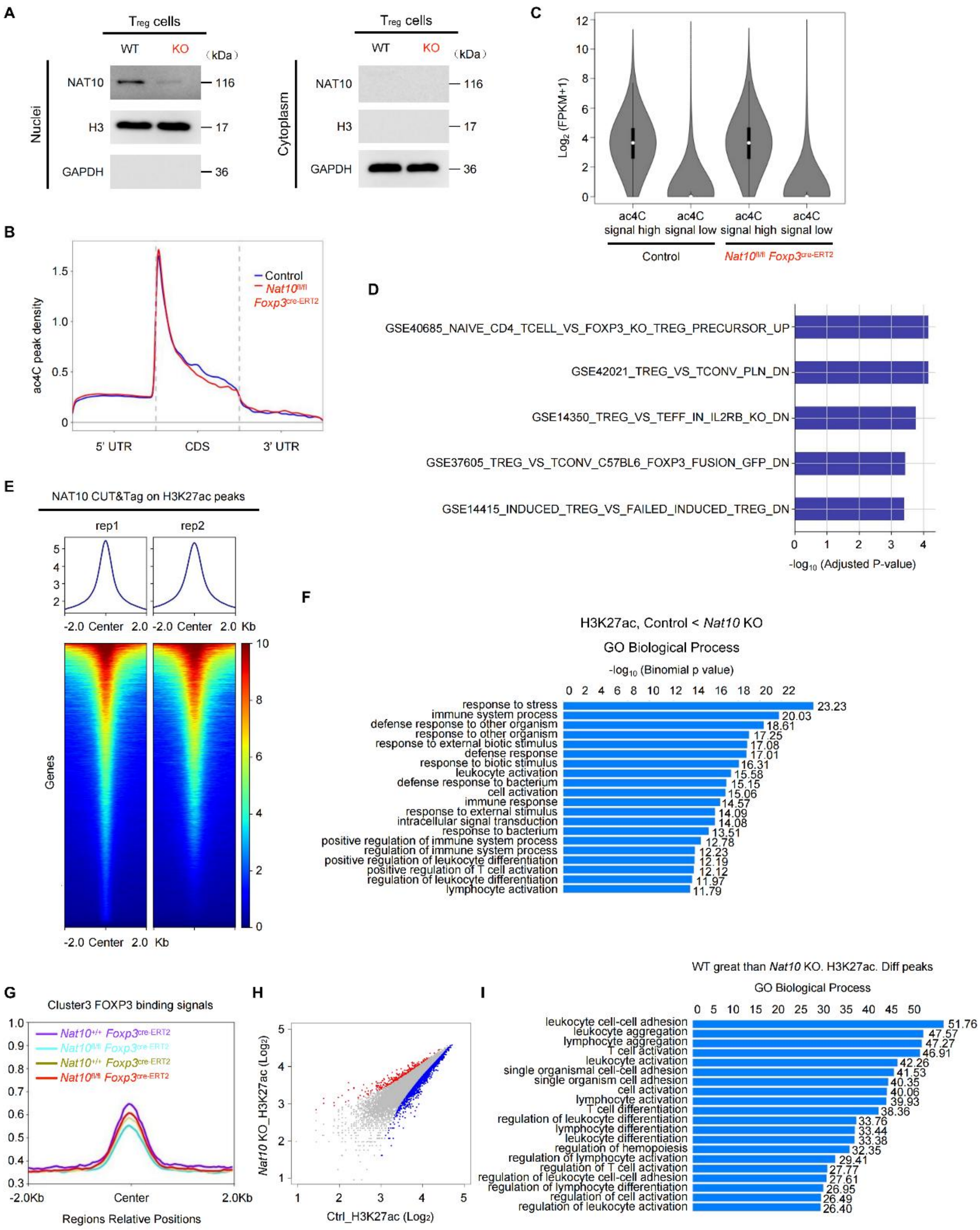

Supplemental Figure 12. ***Nat10*** **ablation does not affect ac4C signal in TI T_{reg} cells.** **(A)** Western blot showing NAT10 protein expression in cellular nuclei or cytoplasm. **(B)** ac4C peak density between *Nat10*$^{+/+}$ *Foxp3*$^{cre\text{-}ERT2}$ and *Nat10*$^{fl/fl}$ *Foxp3*$^{cre\text{-}ERT2}$ in TI T_{reg} cells. **(C)** Difference of ac4C signal between *Nat10*$^{+/+}$ *Foxp3*$^{cre\text{-}ERT2}$ and *Nat10*$^{fl/fl}$ *Foxp3*$^{cre\text{-}ERT2}$ in TI T_{reg} cells based on the respective ac4C signal density-enriched genes.

(D) Gene set enrichment analysis of genes within 2k of cluster 3 peak regions (related to Figure 4). **(E)** Heat maps showing the binding of NAT10 on H3K27ac peak regions (related to Figure 4). **(F)** Gene set enrichment analysis of regions with upregulated H3K27ac enrichment in *Nat10*$^{fl/fl}$ *Foxp3*$^{cre\text{-}ERT2}$ in TI T_{reg} cells (related to Figure 4). **(G)** Comparing FOXP3 binding signals in cluster 3 between *Nat10*$^{+/+}$ *Foxp3*$^{cre\text{-}ERT2}$ and *Nat10*$^{fl/fl}$ *Foxp3*$^{cre\text{-}ERT2}$ in TI T_{reg} cells (related to Figure 4). **(H)** Gene set enrichment analysis of regions with loss of H3K27ac enrichment in *Nat10*$^{fl/fl}$ *Foxp3*$^{cre\text{-}ERT2}$ TI T_{reg} cells. **(I)** GO biological process of H3K27ac enrichment upon genetic deletion of *Nat10*.

Supplemental Figure 13

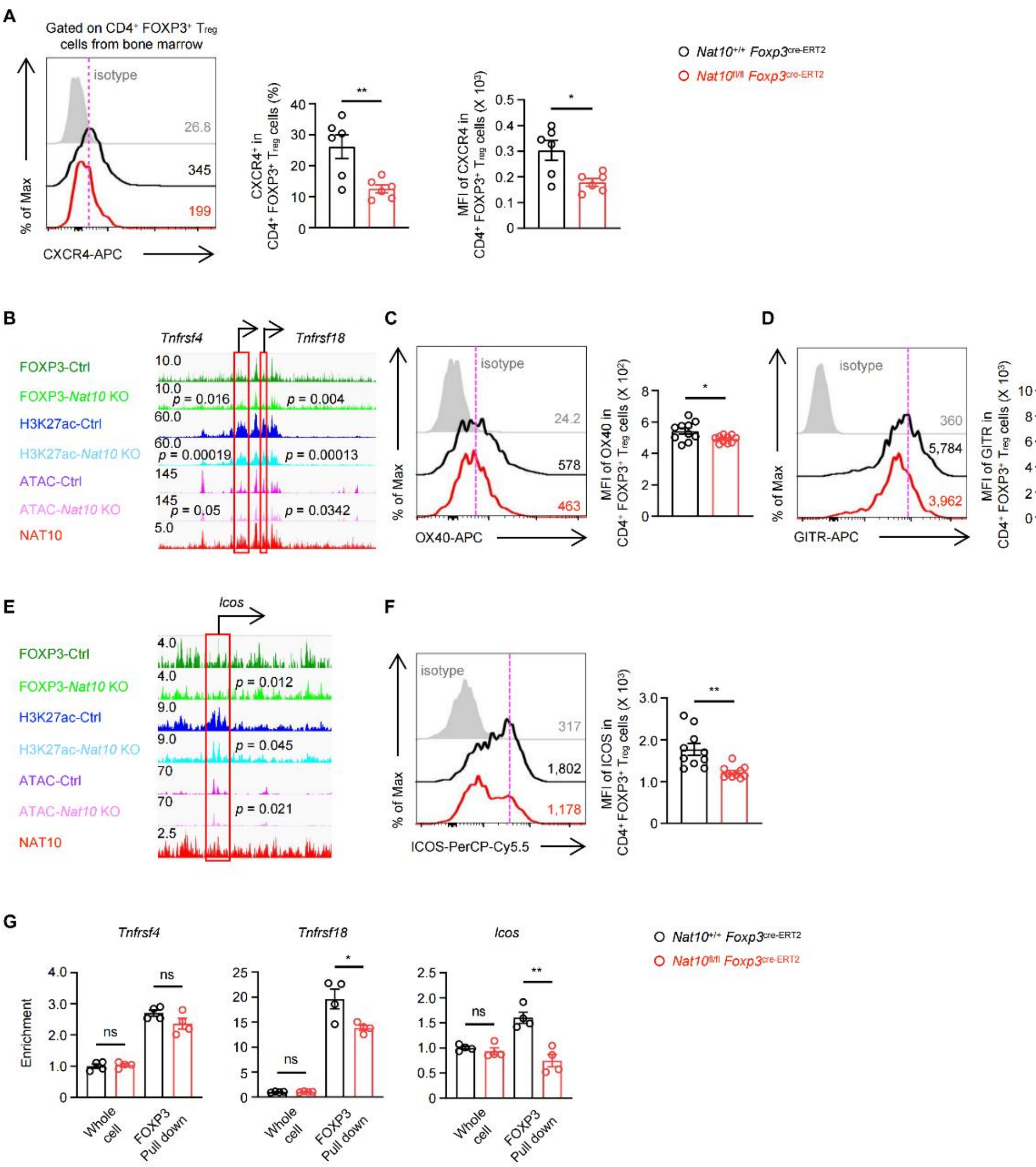

Supplemental Figure 13. NAT10 interacts with FOXP3 to regulate co-stimulatory signals.

(A) Representative histogram (left), quantification of the percentages (middle) and MFI (right) of CXCR4 in T_{reg} cells from the bone marrow of *Nat10*$^{+/+}$ *Foxp3*$^{cre\text{-}ERT2}$ and *Nat10*$^{fl/fl}$ *Foxp3*$^{cre\text{-}ERT2}$ mice intravenously injected with B16F10 cells (n = 6). **(B)** NAT10-CUT&Tag, FOXP3-CUT&Tag, H3K27ac-CUT&Tag, and ATAC-seq tracks at the *Tnfrsf4*, and *Tnfrsf18* gene locus. **(C and D)** Representative histograms (left) and

quantification of the MFI (right) of OX40 **(C)** and GITR **(D)** in TI T_{reg} cells (n = 10). **(E)** NAT10-CUT&Tag, FOXP3-CUT&Tag, H3K27ac-CUT&Tag, and ATAC-seq tracks at the *Icos* gene locus. **(F)** Representative histogram (left) and quantification of the MFI (right) of ICOS in TI T_{reg} cells (n = 10). **(G)** qPCR showing the enrichment of FOXP3 at the promoter of *Tnfrsf4*, *Tnfrsf18* and *Icos* in TI T_{reg} cells (n = 4). Data represent 2-3 independent experiments, mean ± SEM. P values were calculated using two-tailed unpaired Student's *t*-test **(A, C, D, F, G)**.

Supplemental Figure 14

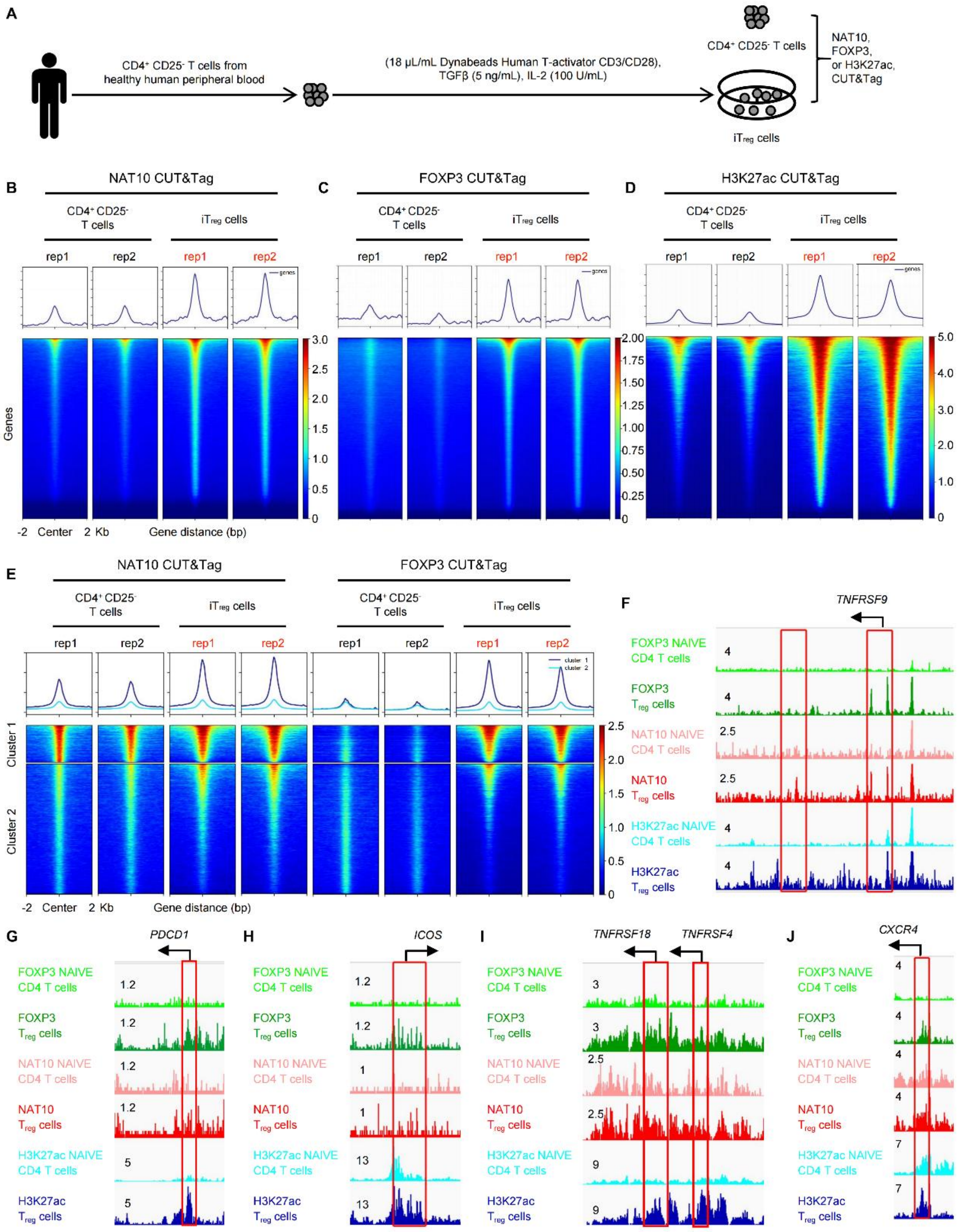

Supplemental Figure 14. In human, NAT10 interacts with FOXP3 to regulate the expression of activation-, immunosuppression-associated markers in iT$_{reg}$ cells that are differentiated from CD4$^+$ CD25$^-$ T cells.

(A) Schematic of the experiment. **(B-D)** Heat maps showing increased enrichment of

NAT10, FOXP3, and H3K27ac peaks in iT_{reg} cells compared to $CD4^+$ $CD25^-$ T cells. **(E)** Heat maps of NAT10 CUT&Tag peaks grouped into two clusters by K-means clustering. Regions co-occupied by NAT10 and FOXP3 in iT_{reg} cells displayed the enhanced signal intensity upon activation. **(F-J)** Genome browser tracks of NAT10-CUT&Tag, FOXP3-CUT&Tag, and H3K27ac-CUT&Tag at the *TNFRSF9* **(F)**, *PDCD1* **(G)**, *ICOS* **(H)**, *TNFRSF4* and *TNFRSF18* **(I)**, and *CXCR4* **(J)** locus.

Supplemental Figure 15

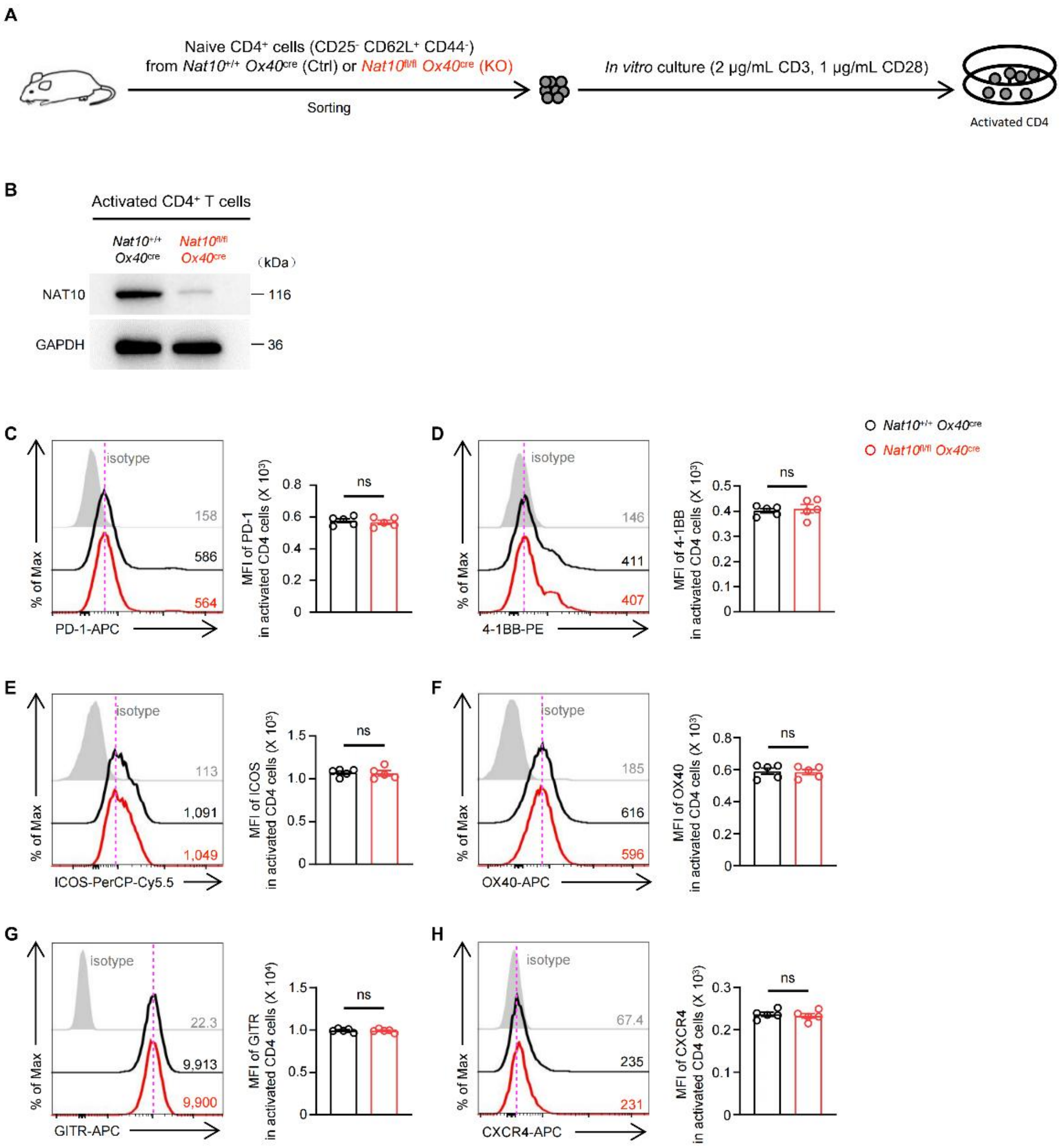

Supplemental Figure 15. NAT10 is not required for the expression of activation- and immunosuppression- associated markers during CD4$^+$ T cell activation.

(A) Schematic of the experiment. **(B)** Western blot showing NAT10 protein expression by using the *in vitro* activated CD4$^+$ T cells of *Nat10*$^{+/+}$ *Ox40*^{cre} and *Nat10*$^{fl/fl}$ *Ox40*^{cre} mice. **(C-H)** Representative histograms (left) and quantification of the MFI (right) of PD-1 **(C)**, 4-1BB **(D)**, ICOS **(E)**, OX40 **(F)**, GITR **(G)**, and CXCR4 **(H)** in activated CD4$^+$ T cells of *Nat10*$^{+/+}$ *Ox40*^{cre} and *Nat10*$^{fl/fl}$ *Ox40*^{cre} mice (n = 5). Data represent 2-3 independent experiments, mean ± SEM. P values were calculated using two-tailed unpaired Student's *t*-test **(C-H)**.

Supplemental Figure 16

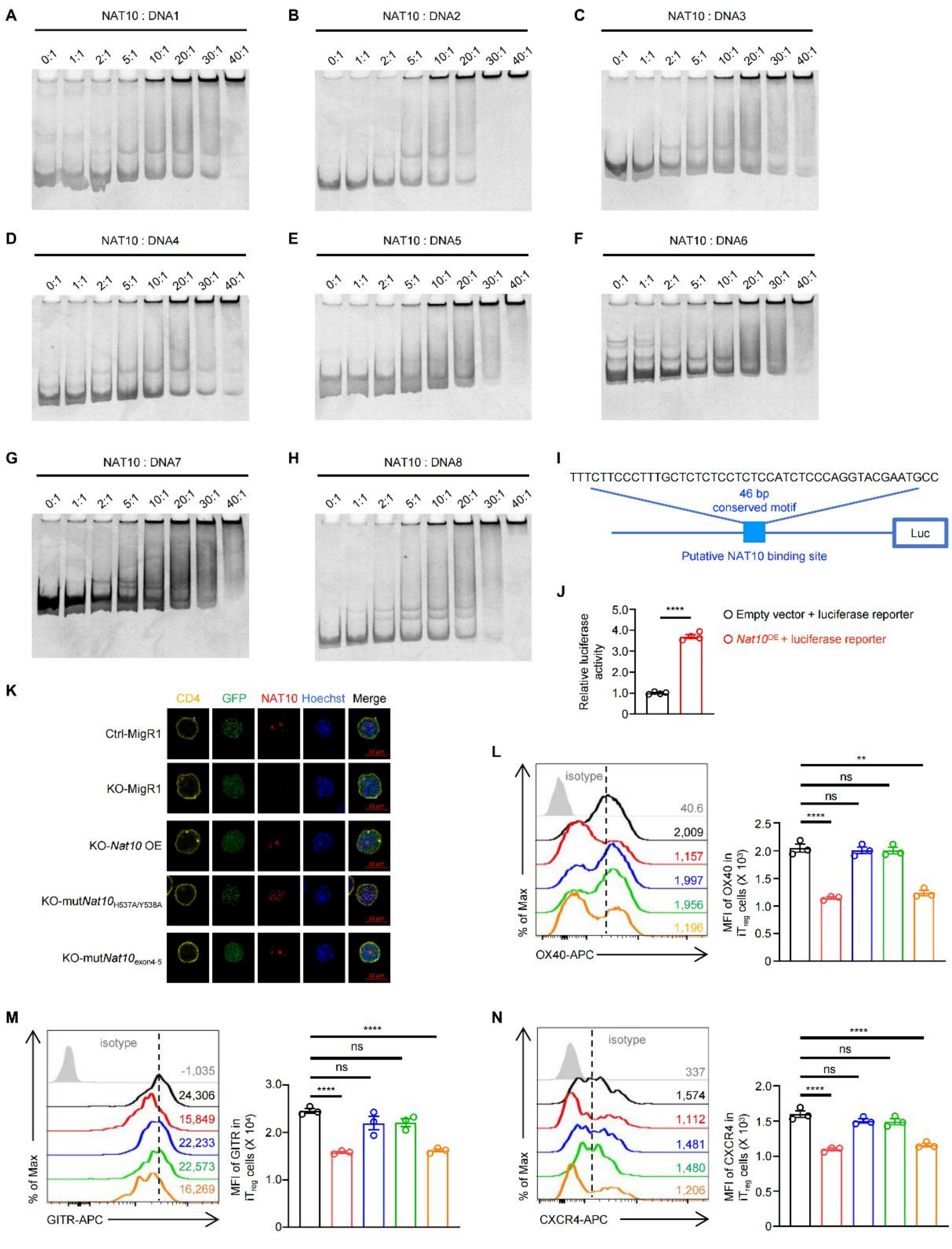

Supplemental Figure 16. EMSA analysis of NAT10 binding to DNA.

(A-H) EMSA analysis of WT NAT10 binding to DNA. **A**, *Pdcd1* (DNA1). **B**, *Pdcd1* (DNA2). **C**, *Cxcr4* (DNA3). **D**, *Cxcr4* (DNA4). **E**, *Tnfrsf9* (DNA5). **F**, *Tnfrsf4* (DNA6). **G**, *Tnfrsf18* (DNA7). **H**, *Icos* (DNA8). **(I)** The putative NAT10 recognition site in the activation-associated gene promoter of TI T_{reg} cells. **(J)** Luciferase activity of

HEK293T cells transfected with empty vector or plasmid expressing *Nat10* (*Nat10*^{OE}) and with a luciferase reporter containing the activation-associated gene promoter of TI T_{reg} cells (46 bp conserved motif) (n = 4). **(K)** Immunofluorescence staining shows the localization of WT NAT10 or different mutant protein in iT_{reg} cells of *Nat10*$^{+/+}$ *Ox40*^{cre} and *Nat10*$^{fl/fl}$ *Ox40*^{cre} mice. **(L-N)** Representative histograms (left) and quantification of the MFI (right) of OX40 **(L)**, GITR **(M)**, and CXCR4 **(N)** in iT_{reg} cells of *Nat10*$^{+/+}$ *Ox40*^{cre} and *Nat10*$^{fl/fl}$ *Ox40*^{cre} mice (n = 3). Data represent 2-3 independent experiments, mean ± SEM. P values were calculated using two-tailed unpaired Student's *t*-test **(J)** and one-way ANOVA **(L-N)**.

Supplemental Figure 17

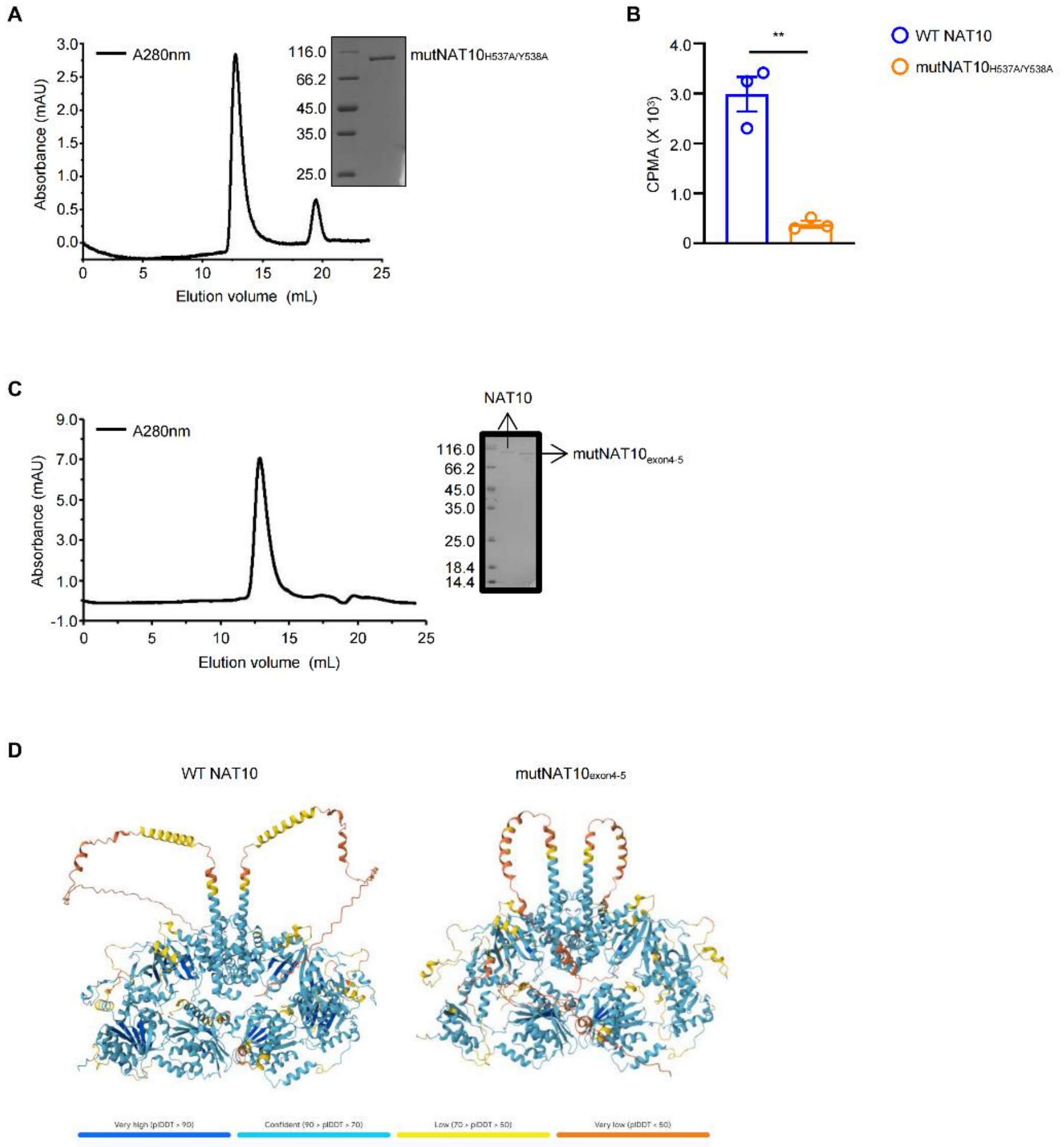

Supplemental Figure 17. Purification of the NAT10$_{H537A/Y538A}$ and NAT10$_{exon4-5}$ mutant, enzyme activity assay and the predicted structure.

(A) Purification of the mutNAT10$_{H537A/Y538A}$. The molecular size peak chart and electrophoresis results show that the mutNAT10$_{H537A/Y538A}$ is successfully purified. **(B)** Enzyme activity test of the mutNAT10$_{H537A/Y538A}$ (n = 3). **(C)** Purification of the mutNAT10$_{exon4-5}$. The molecular size peak chart and electrophoresis test results show that the mutant is successfully purified. **(D)** The structure of WT NAT10 and mutNAT10$_{exon4-5}$ were predicted by using AlphaFold3. Data represent 2-3 independent experiments, mean ± SEM. P values were calculated using two-tailed unpaired Student's *t*-test **(B)**.

Supplemental Figure 18

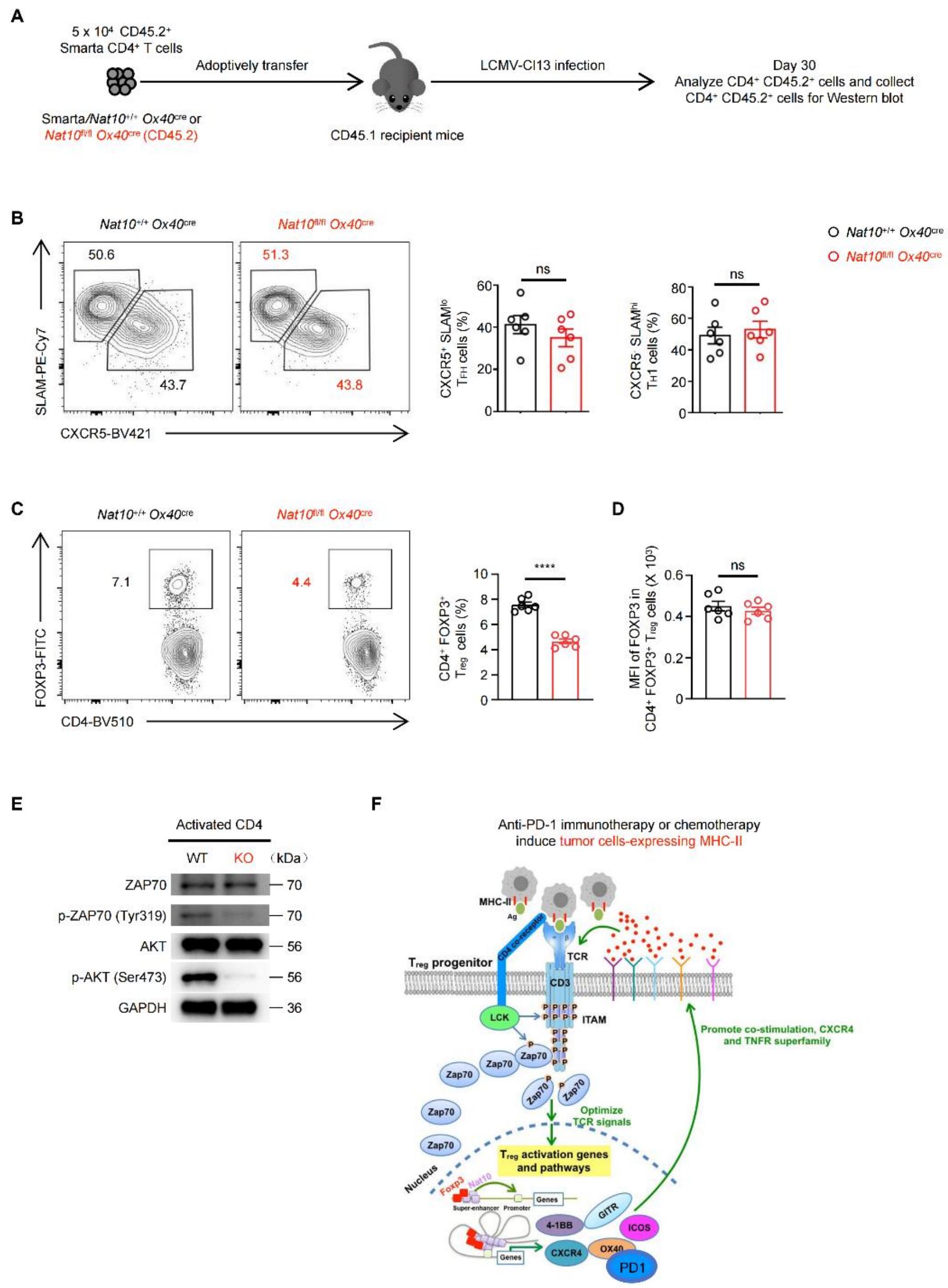

Supplemental Figure 18. ***Nat10*** **ablation shows greatly compromised T_{reg} cells expansion in response to chronic inflammation microenvironment.**

(A) Schematic of the experiment. **(B)** Representative flow cytometry plots (left) and quantification of the percentage (right) of $CXCR5^{+}$ $SLAM^{lo}$ T_{FH} cells and $CXCR5^{-}$ $SLAM^{hi}$ $T_{H}1$ cells (n = 6). **(C and D)** Representative flow cytometry plots (left) and quantification of the percentage (right) of T_{reg} cells **(C)**, and MFI of FOXP3 **(D)** in

CD45.1 mice intravenously injected with CD45.2^{+} Smarta CD4^{+} T cells (n = 6). **(E)** Western blot of ZAP70, phosphorylated of ZAP70 (p-ZAP70), AKT, phosphorylated Akt (p-AKT), and GAPDH using the activated CD45.2^{+} CD4^{+} T cells from panel A. **(F)** Proposal working model of the mechanism in anti-PD-1 immunotherapy or chemotherapy inducing tumor cell-expressing MHC-II. Data represent 2-3 independent experiments, mean ± SEM. P values were calculated using two-tailed unpaired Student's *t*-test **(B-D)**.

Supplemental Figure 19

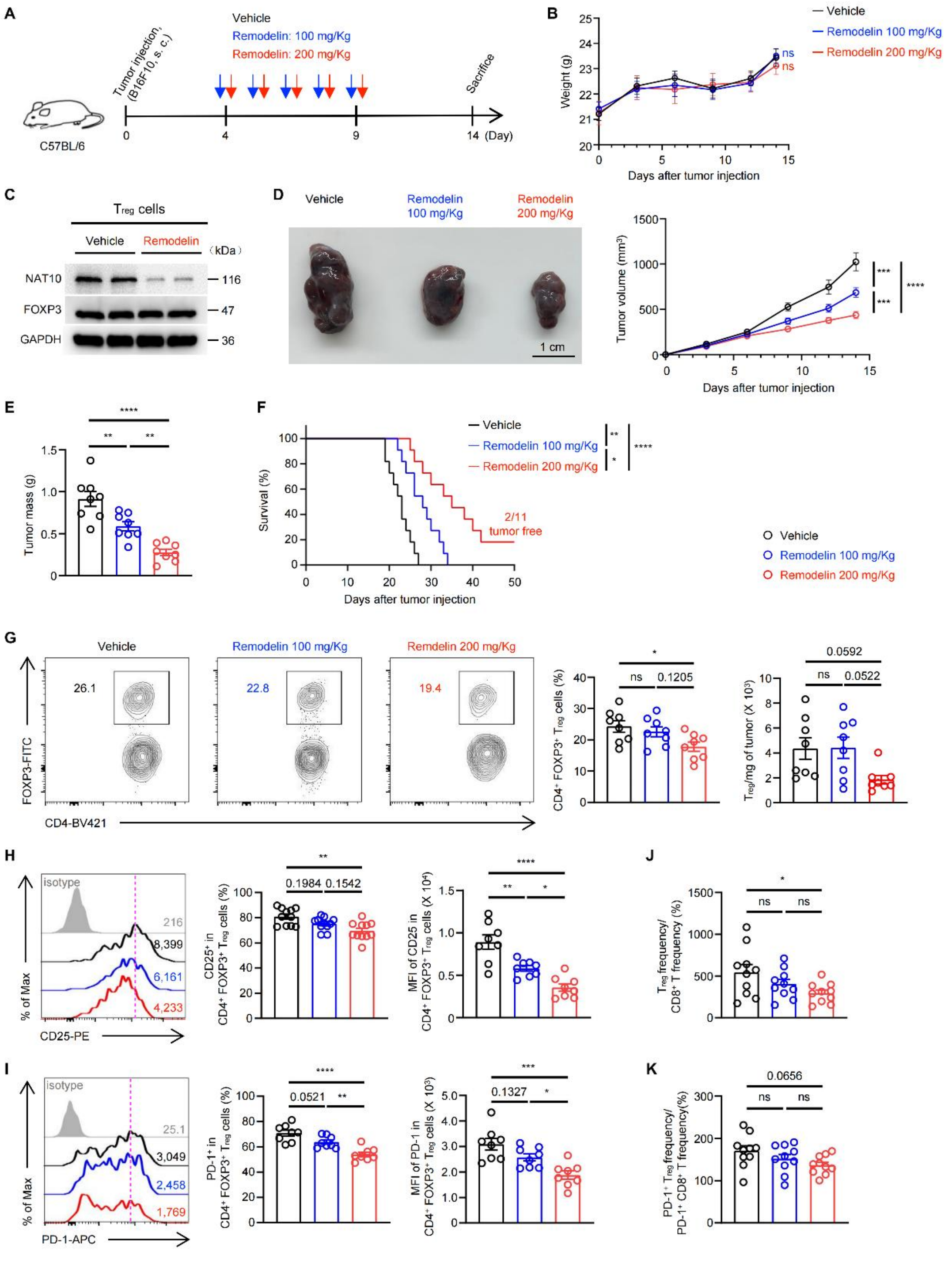

Supplemental Figure 19. Inhibition of NAT10 protein can damage TI T_{reg} cells and promote anti-tumor immunity.

(A) Schematic of the experiment. **(B)** Body weight changes of B16F10 tumor-bearing C57BL/6 mice treated with Vehicle, Remodelin (100 mg/Kg), and Remodelin (200

mg/Kg) (n = 10). **(C)** Western blot for the NAT10 and FOXP3 protein level in T_{reg} cells from B16F10 tumor-bearing C57BL/6 mice treated with Vehicle and Remodelin (200 mg/Kg). **(D and E)** Tumor growth rate **(D)** and tumor mass **(E)** in B16F10 tumor-bearing C57BL/6 mice treated with Vehicle, Remodelin (100 mg/Kg), and Remodelin (200 mg/Kg) at day 14 post-subcutaneous injection with B16F10 cells (n = 8). **(F)** Survival curve of B16F10 tumor-bearing C57BL/6 mice treated with Vehicle, Remodelin (100 mg/Kg), and Remodelin (200 mg/Kg) (n = 11). **(G)** Representative flow cytometry plots (left), quantification of the percentage (middle) and number (right) of TI T_{reg} cells from C57BL/6 mice treated with Vehicle, Remodelin (100 mg/Kg), and Remodelin (200 mg/Kg) (n = 8). **(H and I)** Representative histograms (left), quantification of the percentages (middle) and MFI (right) of CD25 **(H)** and PD-1 **(I)** in TI T_{reg} cells from B16F10 tumor-bearing C57BL/6 mice treated with Vehicle, Remodelin (100 mg/Kg), and Remodelin (200 mg/Kg) (n = 8). **(J and K)** Ratio of T_{reg} cells to $CD8^+$ T cells **(J)** and PD-1^+ T_{reg} cells to $CD8^+$ T cells **(K)** in B16F10 tumor-bearing C57BL/6 mice treated with Vehicle, Remodelin (100 mg/Kg), and Remodelin (200 mg/Kg) (n = 10). Data represent 2-3 independent experiments, mean ± SEM. P values were calculated using one-way ANOVA **(E, G-K),** two-way ANOVA **(B, D)** and log-rank test **(F)**.

Supplemental Figure 20

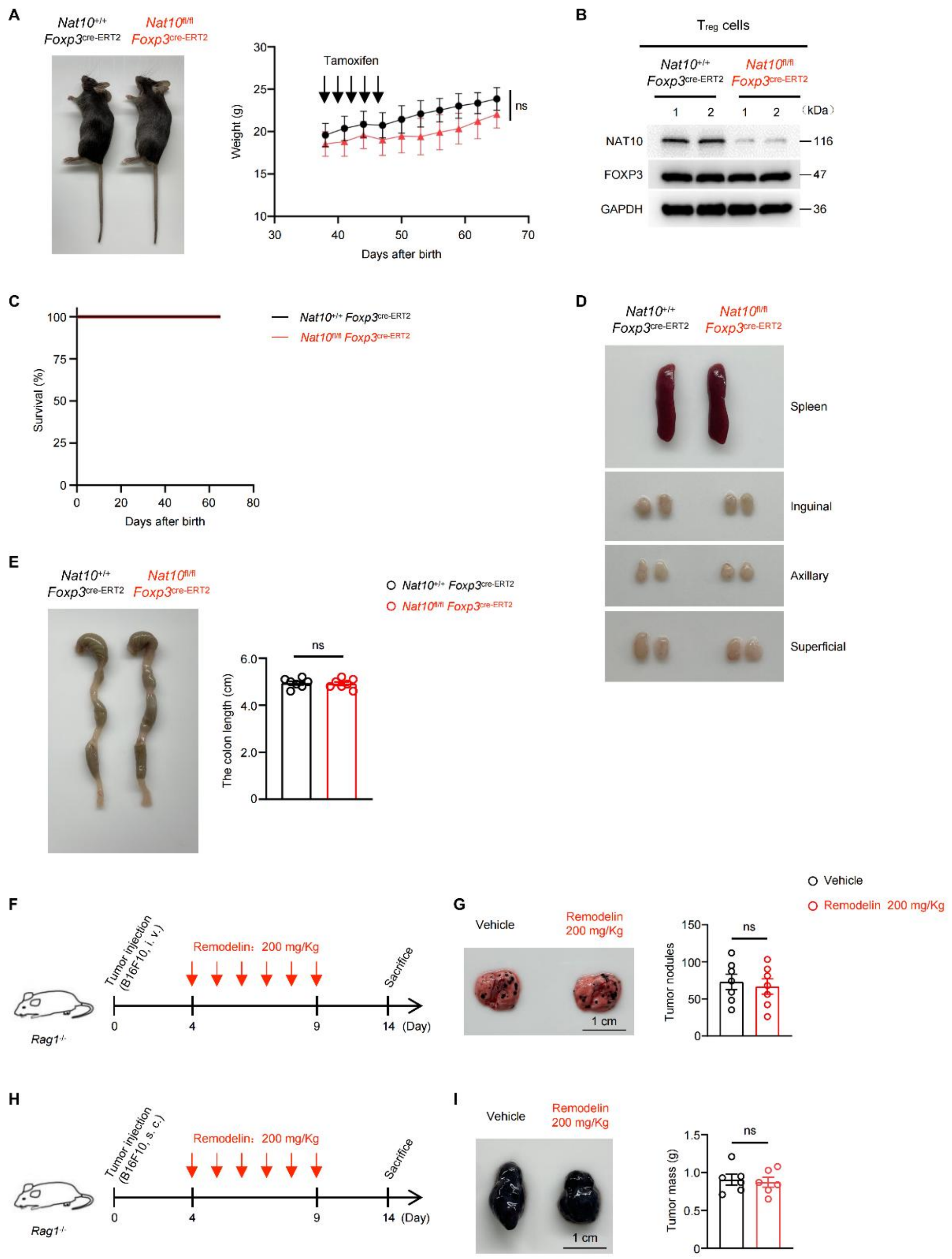

Supplemental Figure 20. NAT10 inhibitors control tumor growth through lymphocytes in metastatic lung cancer and subcutaneous tumor, without leading lethal autoimmune diseases.

(A) Image (left) and body weight changes (right) of *Nat10*$^{+/+}$ *Foxp3*$^{\text{cre-ERT2}}$ and *Nat10*$^{\text{fl/fl}}$

Foxp3$^{\text{cre-ERT2}}$ mice treated with tamoxifen (n = 6). **(B)** Western blot for the NAT10 and FOXP3 protein level in T_{reg} cells from *Nat10*$^{+/+}$ *Foxp3*$^{\text{cre-ERT2}}$ and *Nat10*$^{\text{fl/fl}}$ *Foxp3*$^{\text{cre-ERT2}}$ mice treated with tamoxifen. **(C)** Survival curve of *Nat10*$^{+/+}$ *Foxp3*$^{\text{cre-ERT2}}$ and *Nat10*$^{\text{fl/fl}}$ *Foxp3*$^{\text{cre-ERT2}}$ treated with tamoxifen (n = 6). **(D)** Images of the spleen, inLN (inguinal lymph node), axLN (axillary lymph node), and scLN (superficial neck lymph node) from *Nat10*$^{+/+}$ *Foxp3*$^{\text{cre-ERT2}}$ and *Nat10*$^{\text{fl/fl}}$ *Foxp3*$^{\text{cre-ERT2}}$ mice treated with tamoxifen. **(E)** Image (left) and quantification of the length (right) of colon from *Nat10*$^{+/+}$ *Foxp3*$^{\text{cre-ERT2}}$ and *Nat10*$^{\text{fl/fl}}$ *Foxp3*$^{\text{cre-ERT2}}$ mice treated with tamoxifen (n = 7). **(F)** Schematic of the experiment. **(G)** Quantification of tumor nodules in the lungs of *Rag1*$^{-/-}$ mice treated with Vehicle and 200 mg/Kg Remodelin at day 14 post-intravenous injection with B16F10 cells (n = 7). **(H)** Schematic of the experiment. **(I)** Quantification of tumor mass of *Rag1*$^{-/-}$ mice treated with Vehicle and 200 mg/Kg Remodelin at day 14 post-subcutaneous injection with B16F10 cells (n = 6). Data represent 2-3 independent experiments, mean ± SEM. P values were calculated using two-tailed unpaired Student's *t*-test **(E, G, I),** two-way ANOVA **(A)** and log-rank test **(C)**.

Supplemental Figure 21

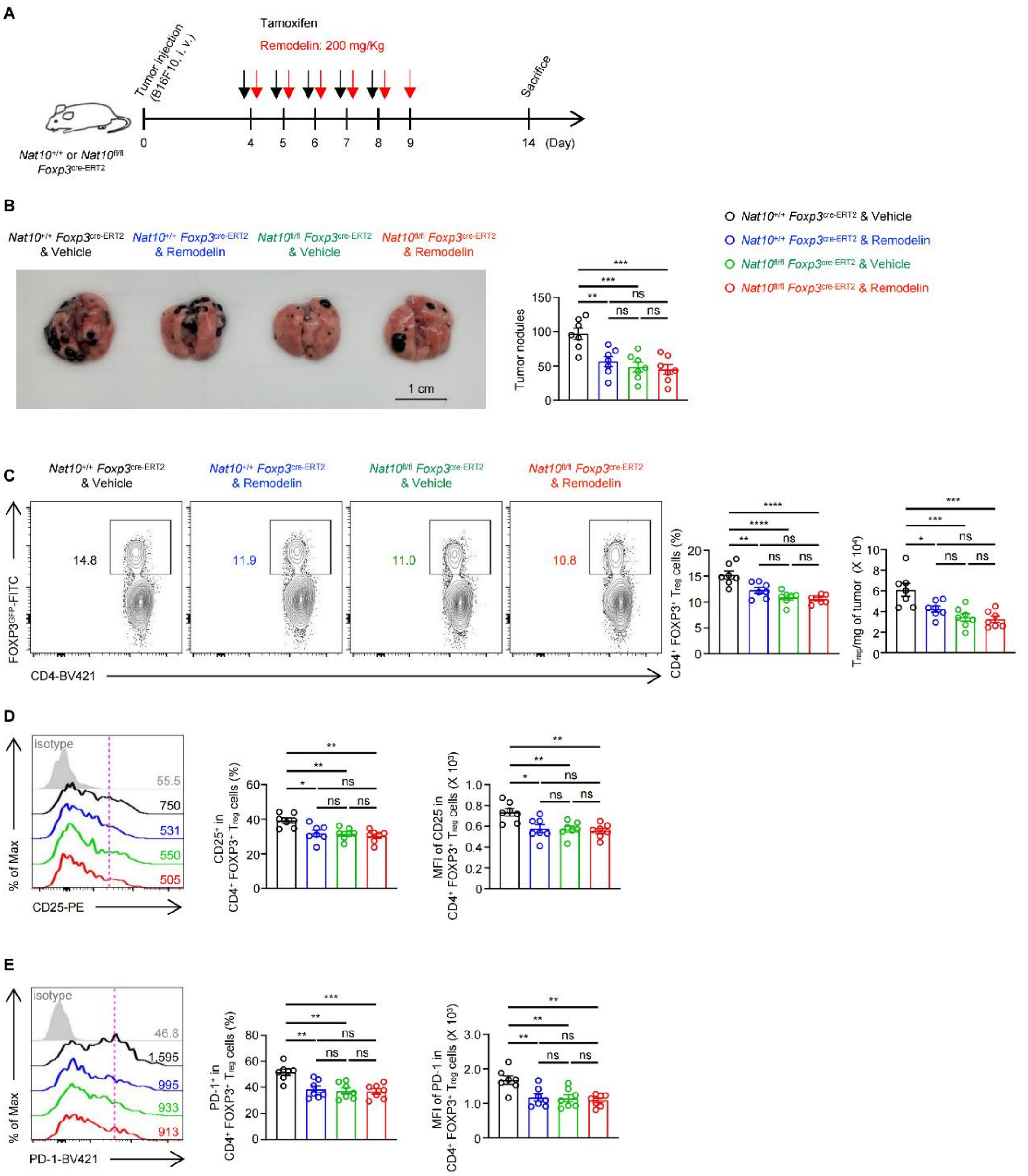

Supplemental Figure 21. Genetic ablation of *Nat10* in T_{reg} cells with Remodelin treatment does not show more significant impairments on tumor progression.

(A) Schematic of the experiment. **(B)** Quantification of tumor nodules in the lungs of *Nat10*$^{+/+}$ *Foxp3*$^{cre\text{-}ERT2}$ & Vehicle, *Nat10*$^{+/+}$ *Foxp3*$^{cre\text{-}ERT2}$ & Remodelin, *Nat10*$^{fl/fl}$ *Foxp3*$^{cre\text{-}ERT2}$ & Vehicle, and *Nat10*$^{fl/fl}$ *Foxp3*$^{cre\text{-}ERT2}$ & Remodelin treated mice (n = 7). **(C)** Representative flow cytometry plots (left), quantification of the percentage (middle)

and number (right) of TI T_{reg} cells in *Nat10*$^{+/+}$ *Foxp3*$^{cre\text{-}ERT2}$ & Vehicle, *Nat10*$^{+/+}$ *Foxp3*$^{cre\text{-}ERT2}$ & Remodelin, *Nat10*$^{fl/fl}$ *Foxp3*$^{cre\text{-}ERT2}$ & Vehicle, and *Nat10*$^{fl/fl}$ *Foxp3*$^{cre\text{-}ERT2}$ & Remodelin treated mice (n = 7). **(D and E)** Representative histograms (left), quantification of the percentages (middle) and MFI (right) of CD25 **(D)** and PD-1 **(E)** in TI T_{reg} cells from *Nat10*$^{+/+}$ *Foxp3*$^{cre\text{-}ERT2}$ & Vehicle, *Nat10*$^{+/+}$ *Foxp3*$^{cre\text{-}ERT2}$ & Remodelin, *Nat10*$^{fl/fl}$ *Foxp3*$^{cre\text{-}ERT2}$ & Vehicle, and *Nat10*$^{fl/fl}$ *Foxp3*$^{cre\text{-}ERT2}$ & Remodelin treated mice (n = 7). Data represent 2-3 independent experiments, mean ± SEM. P values were calculated using one-way ANOVA **(B-E)**.

Supplemental Figure 22

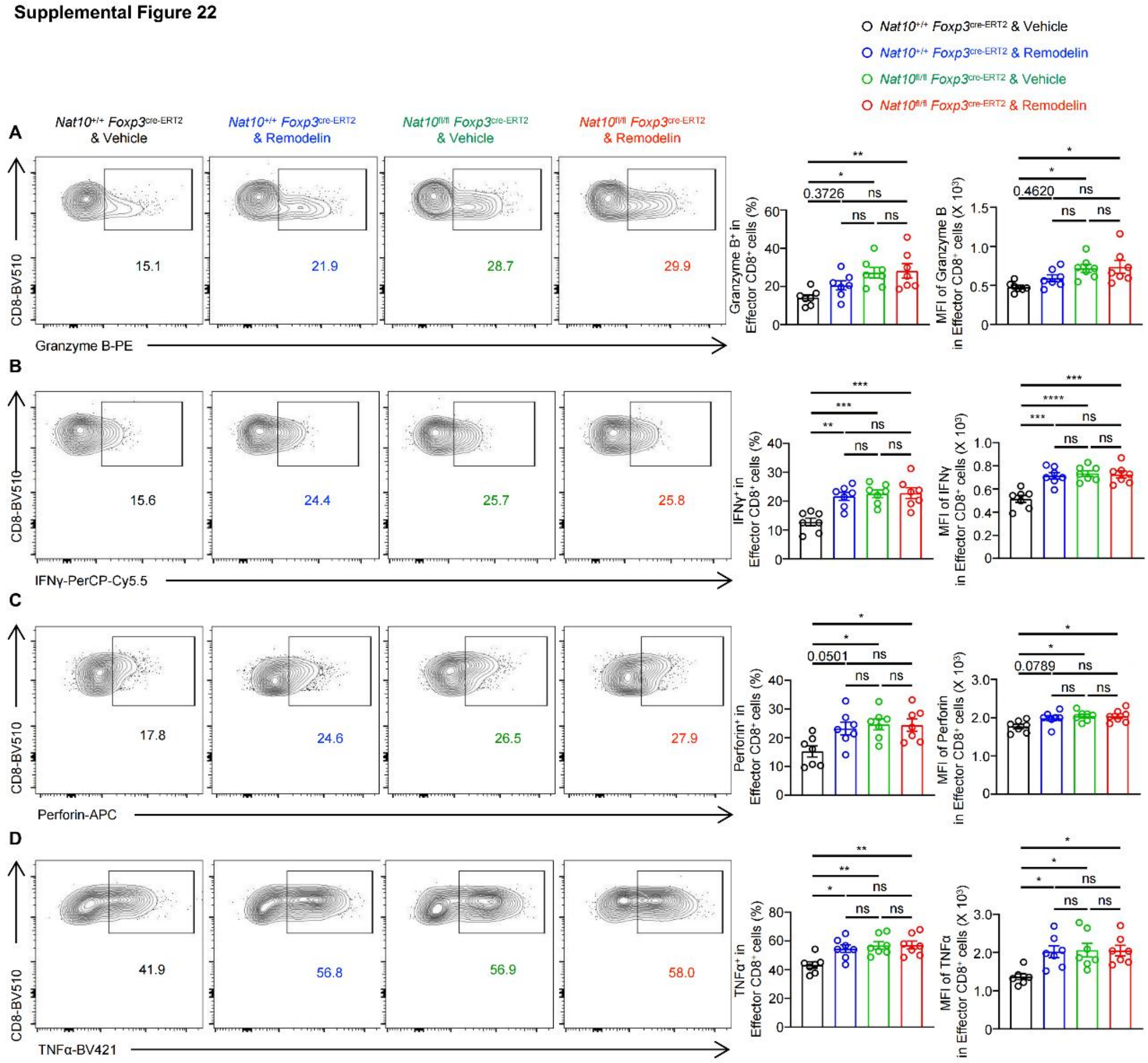

Supplemental Figure 22. Genetic ablation of *Nat10* in T_{reg} cells with Remodelin treatment does not show enhanced cytotoxic function of $CD8^+$ T cells.

(A-D) Representative flow cytometry plots (left), quantification of the percentages (middle) and MFI (right) of Granzyme B **(A)**, IFNγ **(B)**, Perforin **(C)**, and TNFα **(D)** in TI $CD8^+$ cells after *ex vivo* stimulation from *Nat10*$^{+/+}$ *Foxp3*$^{cre\text{-}ERT2}$ & Vehicle, *Nat10*$^{+/+}$ *Foxp3*$^{cre\text{-}ERT2}$ & Remodelin, *Nat10*$^{fl/fl}$ *Foxp3*$^{cre\text{-}ERT2}$ & Vehicle, and *Nat10*$^{fl/fl}$ *Foxp3*$^{cre\text{-}ERT2}$ & Remodelin treated mice (n = 7). Data represent 2-3 independent experiments, mean ± SEM. P values were calculated using one-way ANOVA **(A-D)**.

Supplemental Figure 23

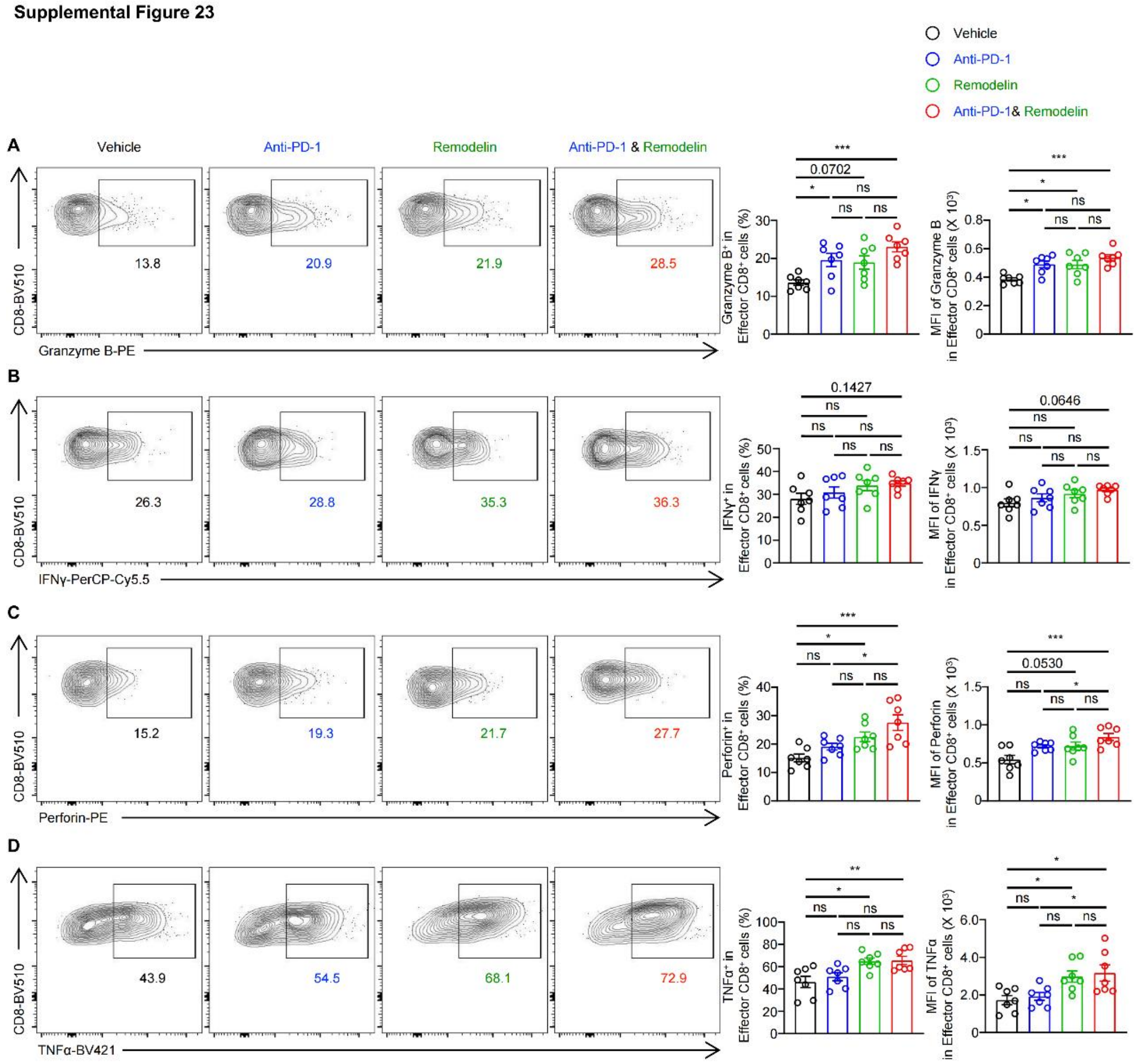

Supplemental Figure 23. Inhibition of NAT10 synergizes with anti-PD-1 to enhance CD8⁺ T cells anti-tumor immunity.

(A-D) Representative flow cytometry plots (left), quantification of the percentages (middle) and MFI (right) of Granzyme B **(A)**, IFNγ **(B)**, Perforin **(C)**, and TNFα **(D)** in TI CD8⁺ cells after *ex vivo* stimulation from B16F10 tumor-bearing C57BL/6 mice treated with Vehicle, anti-PD-1, Remodelin, and anti-PD-1 & Remodelin (n = 7). Data represent 2-3 independent experiments, mean ± SEM. P values were calculated using one-way ANOVA **(A-D)**.

Supplemental Figure 24

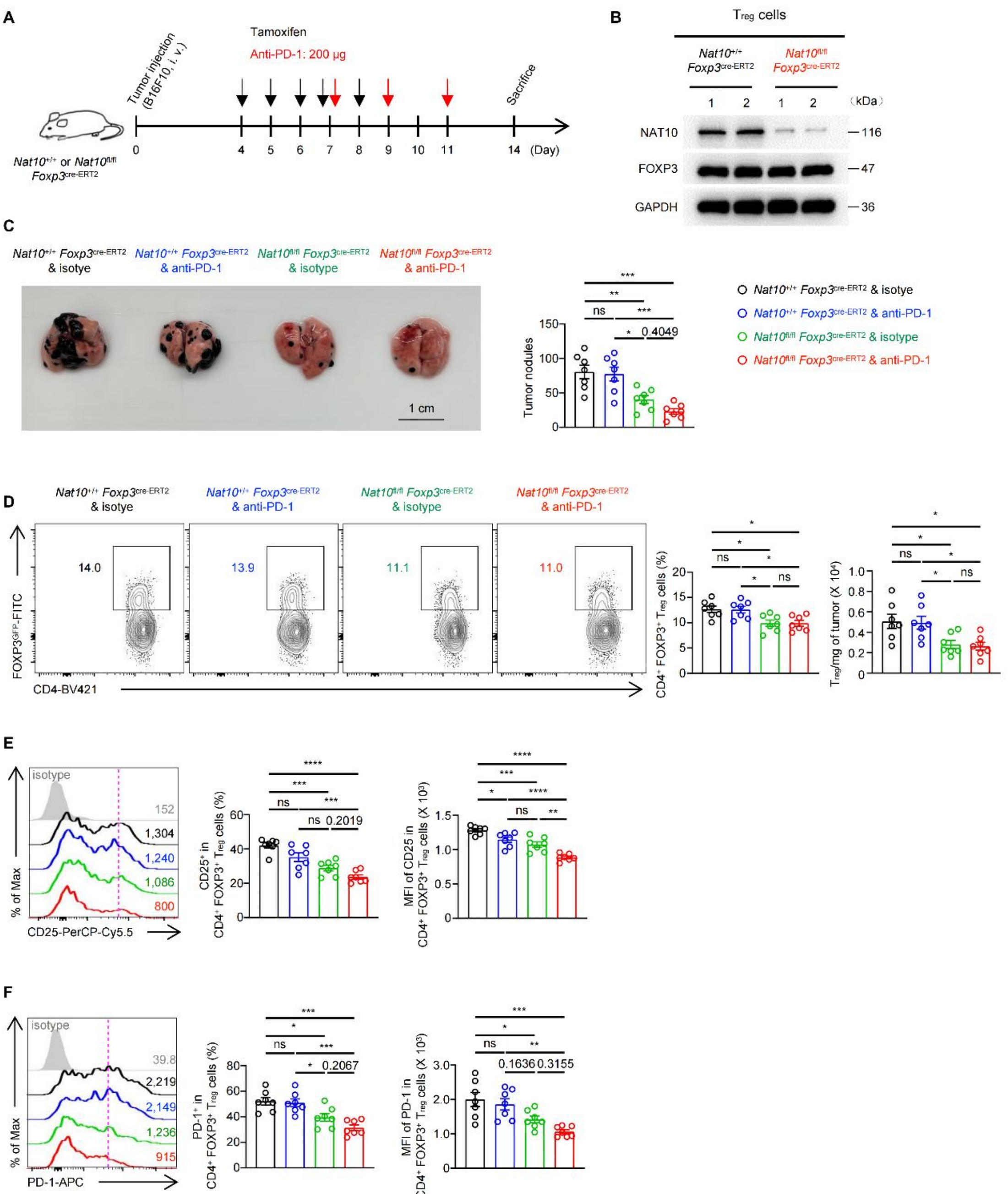

Supplemental Figure 24. Genetic ablation of *Nat10* in T_{reg} cells synergizes with anti-PD-1 therapy to enhance control of tumor growth.

(A) Schematic of the experiment. **(B)** Western blot for the NAT10 and FOXP3 protein level in T_{reg} cells from *Nat10*$^{+/+}$ *Foxp3*$^{cre\text{-}ERT2}$ and *Nat10*$^{fl/fl}$ *Foxp3*$^{cre\text{-}ERT2}$ mice treated with tamoxifen. **(C)** Quantification of tumor nodules in the lungs of *Nat10*$^{+/+}$ *Foxp3*$^{cre\text{-}}$

^{ERT2} & isotype, *Nat10*$^{+/+}$ *Foxp3*$^{cre-ERT2}$ & anti-PD-1, *Nat10*$^{fl/fl}$ *Foxp3*$^{cre-ERT2}$ & isotype, and *Nat10*$^{fl/fl}$ *Foxp3*$^{cre-ERT2}$ & anti-PD-1 treated mice (n = 7). **(D)** Representative flow cytometry plots (left), quantification of the percentage (middle) and number (right) of TI T_{reg} cells in *Nat10*$^{+/+}$ *Foxp3*$^{cre-ERT2}$ & isotype, *Nat10*$^{+/+}$ *Foxp3*$^{cre-ERT2}$ & anti-PD-1, *Nat10*$^{fl/fl}$ *Foxp3*$^{cre-ERT2}$ & isotype, and *Nat10*$^{fl/fl}$ *Foxp3*$^{cre-ERT2}$ & anti-PD-1 treated mice (n = 7). **(E and F)** Representative histograms (left), quantification of the percentages (middle) and MFI (right) of CD25 **(E)** and PD-1 **(F)** in TI T_{reg} cells from *Nat10*$^{+/+}$ *Foxp3*$^{cre-ERT2}$ & isotype, *Nat10*$^{+/+}$ *Foxp3*$^{cre-ERT2}$ & anti-PD-1, *Nat10*$^{fl/fl}$ *Foxp3*$^{cre-ERT2}$ & isotype, and *Nat10*$^{fl/fl}$ *Foxp3*$^{cre-ERT2}$ & anti-PD-1 treated mice (n = 7). Data represent 2-3 independent experiments, mean ± SEM. P values were calculated using one-way ANOVA **(C-F)**.

Supplemental Figure 25

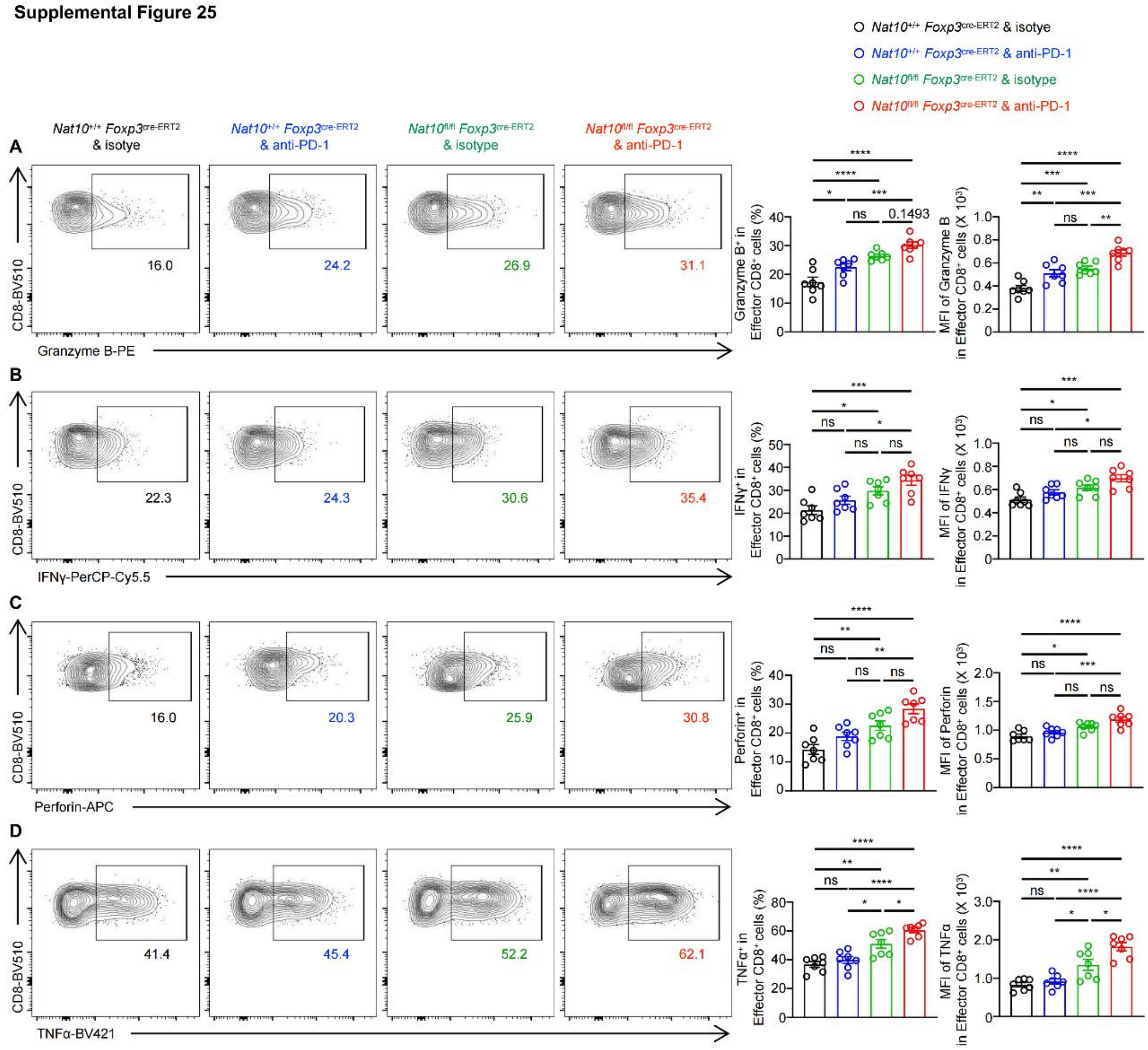

Supplemental Figure 25. Genetic ablation of *Nat10* in T_{reg} cells synergizes with anti-PD-1 therapy to enhance $CD8^+$ T cells anti-tumor immunity.

(A-D) Representative flow cytometry plots (left), quantification of the percentages (middle) and MFI (right) of Granzyme B **(A)**, IFNγ **(B)**, Perforin **(C)**, and TNFα **(D)** in TI $CD8^+$ cells after *ex vivo* stimulation from *Nat10*$^{+/+}$ *Foxp3*$^{cre\text{-}ERT2}$ & isotype, *Nat10*$^{+/+}$ *Foxp3*$^{cre\text{-}ERT2}$ & anti-PD-1, *Nat10*$^{fl/fl}$ *Foxp3*$^{cre\text{-}ERT2}$ & isotype, and *Nat10*$^{fl/fl}$ *Foxp3*$^{cre\text{-}ERT2}$ & anti-PD-1 treated mice (n = 7). Data represent 2-3 independent experiments, mean ± SEM. P values were calculated using one-way ANOVA **(A-D)**.

Supplemental Table 1

RNA epigenetic modifier genes
ALKBH5, *APOBEC3C*, *APOBEC3D*, *CBLL1*, *CELF1*, *CTR9*, *DDX17*, *DHX9*, *EXOSC1*, *EXOSC4*, *EXOSC7*, *EXOSC9*, *FTO*, *HNRNPU*, *KHDRBS1*, *LEO1*, *METTL3*, *MOV10*, *NAT10*, *NCL*, *NSUN6*, *NUP98*, *PAF1*, *PQBP1*, *PRPF31*, *PTBP1*, *PUF60*, *RBM17*, *RBM4*, *BM5*, *RBM7*, *RBM8A*, *RNPS1*, *RPUSD3*, *SF3B1*, *SF3B3*, *SFPQ*, *SLU7*, *SRSF1*, *SRSF3*, *SRSF6*, *SYNCRIP*, *TAF10*, *TAF12*, *TAF2*, *TAF7*, *TAF9*, *TAF9B*, *THRAP3*, *TRA2B*, *TRUB2*, *TYW5*, *WTAP*, *YTHDC1*

Supplemental Table 2

Sample	Tumor type	Sex	Age	Stage	Histology	Smoking	Prior treatment	Mutational
133791	Adenocarcinoma	F	49	pT1N0 M0 Ia	CK7 (+)<br>TTF-1 (+)<br>Napsin-A (+)<br>P40 (-)<br>PDL1 （-）	No	No	No
133926	Adenocarcinoma	F	74	pT1cN 2M0 IIIa	CK7 (+)<br>TTF-1 (+)<br>Napsin-A (+)<br>P40 (-)<br>PDL1 8%<br>Syn（-）<br>P53(-)	No	No	No
134659	Adenocarcinoma	M	67	cT4N2 M1c IVb	CK7 (-)<br>TTF-1 (-)<br>Napsin-A (-)<br>Ki-67 70%<br>Syn（-）<br>CK19 （+）	No	No	No
134306 (Figure 1E)	Adenocarcinoma	M	64	cT1N2 M1a IVa	CK7 (-)<br>Napsin-A (-)	No	No	No
135450	Adenocarcinoma	F	53	cT4N3 M1c IVb	CK7 (+)<br>TTF-1 (+)<br>Napsin-A (+)	No	No	No

					PDL1 70%			
136068	Adenocarcinoma	M	63	cT2aN2 M1a IVa	CK7 (+) TTF-1 (+) Napsin-A (+) P40 (-) PDL1 （-） Ki-67 30% cMET(++)	No	No	No
136070	Adenocarcinoma	M	80	rT0N0 M1a IVa	TTF-1 (+) Napsin-A (+) PDL1 1%	1pack*50 years	lobectomy, four cycles of carboplatin, pemetrexed, and sintilimab	No
135962 (Figure 1E)	Adenocarcinoma	M	59	pT1N0 M0 Ia	CK7 (+) TTF-1 (+) Napsin-A (+) P40 (-) Ki-67 (+)	1pack*40 years	No	No
122266	Adenocarcinoma	F	50	pT2aN 0M0 Ib	CK7 (+) TTF-1 (+) Napsin-A (+) CK5/6 (-) PDL1 （-） Ki-67 40% ALK(-)PDL1 1%	No	No	No
4108	Adenocarcinoma	F	51	pT1cN 0M0 Ia	CK7 (+) TTF-1 (+) Napsin-A (+) P40 (-) Ki-67 (+)	No	No	No
1697	Adenocarcinoma	F	71	pT1aN 0M0 Ia	CK7 (+) Napsin-A (+) P40 (-) Ki-67 (+)	No	No	No
3631	Adenocarcinoma	F	59	pT2aN 0M0 Ib	CK7 (+) TTF-1 (+) Napsin-A (+) P40 (-) Ki-67 (+)	No	No	No

2867	Adenocarcinoma	M	68	pT4N2<br>M0 IIIa	CK7 (+)<br>TTF-1 (+)<br>Napsin-A (+)<br>P40 (-)<br>Ki-67 (+)	No	No	No
0570	Adenocarcinoma	F	74	pT2aN<br>0M0 Ib	TTF-1 (+)<br>Napsin-A (+)<br>Ki-67 (+)	No	No	No
1165	Adenocarcinoma	F	50	pT2aN<br>0M0 Ib	CK7 (+)<br>TTF-1 (+)<br>Napsin-A (+)<br>P40 (-)<br>Ki-67 (+)	No	No	No
2849	Adenocarcinoma	M	70	pT3N0<br>M0 IIb	CK7 (+)<br>TTF-1 (+)<br>Napsin-A (+)<br>P40 (-)<br>Ki-67 (+)	No	No	No
2855	Adenocarcinoma	M	70	pT3N0<br>M0 IIb	CK7 (+)<br>TTF-1 (+)<br>Napsin-A (+)<br>P40 (-)<br>Ki-67 (+)	1pack*42<br>years	No	No
3791	Adenocarcinoma	M	54	pT1cN<br>0M0 Ia	CK7 (+)<br>TTF-1 (+)<br>Napsin-A (+)<br>P40 (-)<br>Ki-67 (+)	No	No	No
4158	Adenocarcinoma	F	74	pT1cN<br>0M0 Ia	TTF-1 (+)<br>Napsin-A (+)<br>P40 (-)<br>Ki-67 (+)	No	No	No

Supplemental Table 3

**Gene name**		**Length of sequence**	**Sequence**
Pdcd1	DNA1	313bp	GCGCATGGAATTGTGGCCTAGACAAGCTTATACTCTTGTGCTGCCTTAACATCTTTGACCTGCAATGATG<br>TTCCTTTGCTCATATGACGTCCCAGTCACCTTCACCCCAGCCTCTTCACTACCGTGAAAACAGGATGGGA<br>CTGGGAGCTGGGTGCTGGGTGCTTACCACCACAGGCCCTCTCCTGGGTTGCAGGCTAAGGCTGGTTGGGA<br>AGCCAGGGACATTTTCCCTCCACGTACCCTCTTAAGATAGCCCATGGTTTGCTGTCAAGACTGGATGGAG<br>GAAACAGACCTGAACATGGTAGATGGTCTGAGG

Pdcd1	DNA2	286bp	CGCCTATCCCCTCCCACTGTGGTGTCCTTAATCTCTCGCAGAGGCTGCAGCTCTTGATGTGGCTACTCCACCACAAACCAGAGCTGACCACAGGGGCGCAGGACCCAGGCGTCCGAGCTACCAAGCACCCATGCTGCGCCTCACCCAAAAGGTTTCTTCCCTTTGCTCTCTCCTCTCCATCTCCCAGGTACGAATGCCCTCCTTAACTGGTCTGGAAGGGAGAACCAGGCTTCTGTTTCAGTCTGGCCCTGCCGGATGGAGGAGGCTGGAGATGCAGCAGGGAATC
Cxcr4	DNA3	334bp	ATTGTGACTTTCTTCCTGGGAGTATATCTGCTTCTGGATGGGTCCGCAAGTGCTTTCTGCCACACCTGACCCTAATGTCATTATATGAATATGTAGTAACACACACTTGCTTGGAAGAGTTTTGGTAGTAATCACTCCTGACAGCTCAGAGTATAGAACCCTGGCGGAGGTTCCAACATTGCCGCCTACTGGTTAGGCAGGCTCCAAGTCACACACCCCCATCCCCAGTCTTGTCTCAAGTTCTCCACCCGTAAACTCCAGGTCTAAAATTCTATCCACGTGGGTAAGGATGGATGCGCGACCCGCAGAAACACCCTGGCTGGCATCCTGCTTT
Cxcr4	DNA4	301bp	CCCCAACTCTGAGAACATTATCCGGCCAGGCTCTGGACCCATACTGACTTAAAACACAACAGCAGTGGCTCTTGGACTTCTGTTTGCCCGCGCCCTGCGCCCCGCCCCCGCCAGTCTCCTCCCGCCCCGTCCGAGCTGCGCATGCGCCGCCCCGGGAGCGTGTTTTTATAAAAGTCCGGTTCGGGCCAGAAACTTCAATTTTGTTGCCTGGTGCAGCAGGTAGCAGTGAAACCTCTGAGGCGTTTGGTGCTCCGGTAACCACCACGGCTGTAGAGCGAGTGTTGCCATGGAACCGATCAGT
Tnfrsf9	DNA5	420bp	TGGGGGCACGGGTGTGTGTGGGCAGTCACATGCAAGTGCATGTGTGTAGGTCAGAGGAGTCCCCGTGGGAGTAGGTGCTCTCCTTCTACCACATGGTTCACAGGGATTAAACGCAAGCCTTCAAGCTTCAGCAGCAGGTGCTTTTCGCCCAGCTACCTCGCCAGCCCACATCTAAGAACCCCTCCCTACGTCCTGGGTCGGAAGGGTTGAGACTTTCCCCAGTGGTAGTGTCTACGTTTTACACTTTTGTGTTTGACATTTAAAGCTATAAGTTAGCTAGCTATAAAGTGTCAGAACTTAAGGTCGAGACTTTTTCTTTTTGGGGCTAGGGAGCTGTGCTTGCCATATTCCAGTGTTACTCAAAGAAACAGTCTCCCTTCTCTGTCTACCCACATTTTTATTGGCTCTGTGCTCATGAGGG
Tnfrsf4	DNA6	473bp	CCAAAGCACTTCTTAGCTTATCATGGGACTCTGCATACGCCTGTGCCAAATACACAGGAACACGTTCACATACCTTCTTGCCTGTCCGCCTACTCTTCTTGCCCCACCTCCATAGTTCTTATAGCCACACCCTGCAAGGAAAAACCCCAGACTCCTGTGAAGGCAGAAAGCAGACAAGGATGTATGTGTGGGTTCAGCAGCCCACAGCCCTTCTGCTGCTGGGACTCACACTTGGAGTTACAGCAAGGCGGCTCAACTGTGTTAAACATACCTACCCCAGTGGTCACAAGTGCTGTCGTGAGTGCCAGCCAGGTGAGTAGCCTGGCTTTGAGATGTAGTGGCCGGGTGGGGGAGGGGTGATTCTTGGGGCAATGGAGACACAGGTCCAGAATACGTGGGGGTAAATGTCAGAGATTATTGTCACAAATGTGACAATGTCACAGACAGAGGCTGGTCCTGGGAGAGCCACGGTG
Tnfrsf18	DNA7	405bp	CTGCTGAACCTCTCCCCGAATTGAACACCCAATGTCTATGCCTGGGTTTACTTCCTTTTCATTCTGGTGCTGTCCCACAGTACATTCTCTATACCCCCACCTTATCTCAAAAACTCCCTTTACTGAGGCCCCATGTCAGTCTGGGGATTCCCCACAGGGTCGGACTGCCAGGACCCTCTGCTGCTTGGAACACGTTCACTGAATCCTGTGAAACAAACCACGAGGAAACCCAGGGAGCTGCTCGTCAAATCGAGGAGATAACGGCGAGCAGATGAAAATCTCTCTGCCCACCATCCAGTTTTGGAGCTGCTTGTTCTTGGTAGTAGTCGCTGGCAGTAGGTACCTAGAAGTGGGTGGGACAGGAAGTCTGACATAGGTGCTGACATTTGCTGATGTCATCAGCCT
Icos	DNA8	272bp	CTCTATCTCTGACAGTGACTGAACAGTTACTCGAGAATCCCACCATCTCTGGAGAAATAGCCATTTGGTGTTCTTAGCACCAGATCCTCCCCACAGAGCTCAGAGAGGTCTGGGGTGATGAGCTGACCTCTGGGTCCTCCCTTTGAGCACCTGTTTCTAGAATTCAAACTGCATTGAAAAACTCTGCATTGCTAACCTTTTGTCTGGATAGATGAATGTAAGCCATTCTCAGCATGATAAGCCTTGATTTTATGCCTCCAAGAGTGTATGTA
The conserved motif	DNA9	46bp	TTTCTTCCCTTTGCTCTCTCCTCTCCATCTCCCAGGTACGAATGCC
