# Supplementary material for: Targeting N-acetyltransferase 10 reprograms Treg function to enhance anti–PD-1 immunotherapy sensitivity in murine lung tumors
Source: J Clin Invest. 2026 Nov 2;136(21):e189034. doi: 10.1172/JCI189034 (PMC13722382; doi:10.1172/JCI189034)

Full unedited blot for Figure 2B

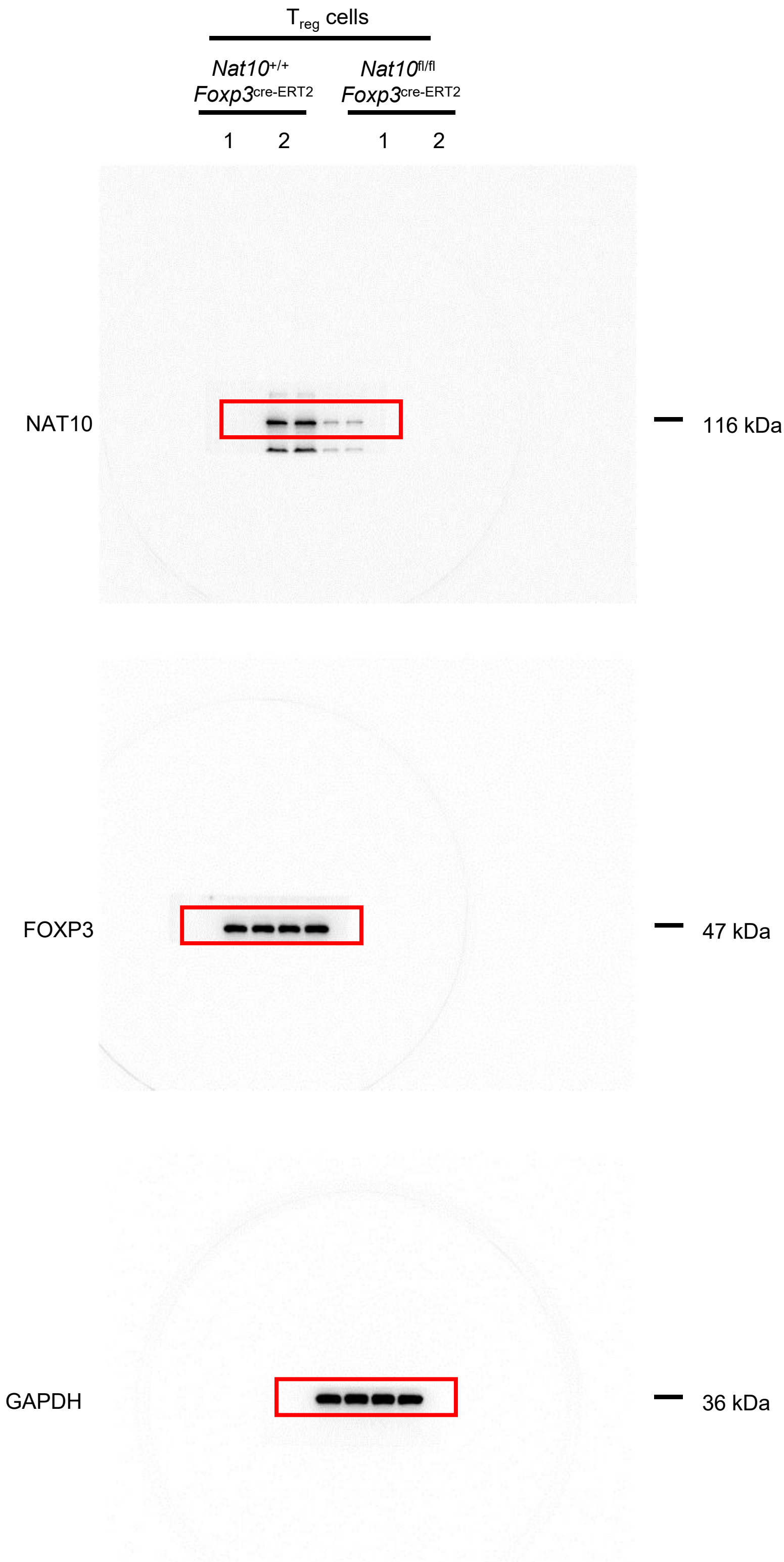

Full unedited blot for Figure 4I

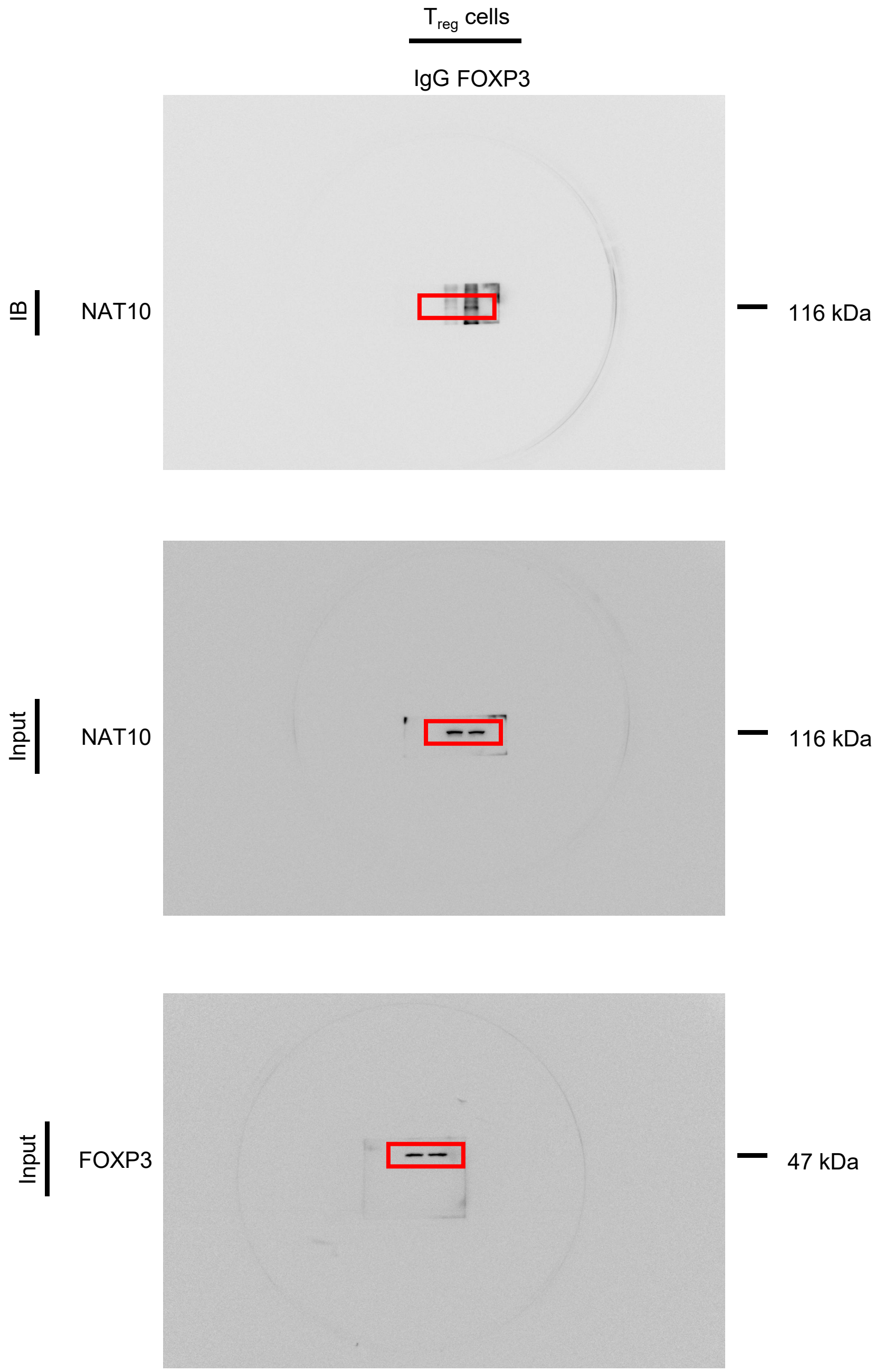

Full unedited blot for Figure 4J

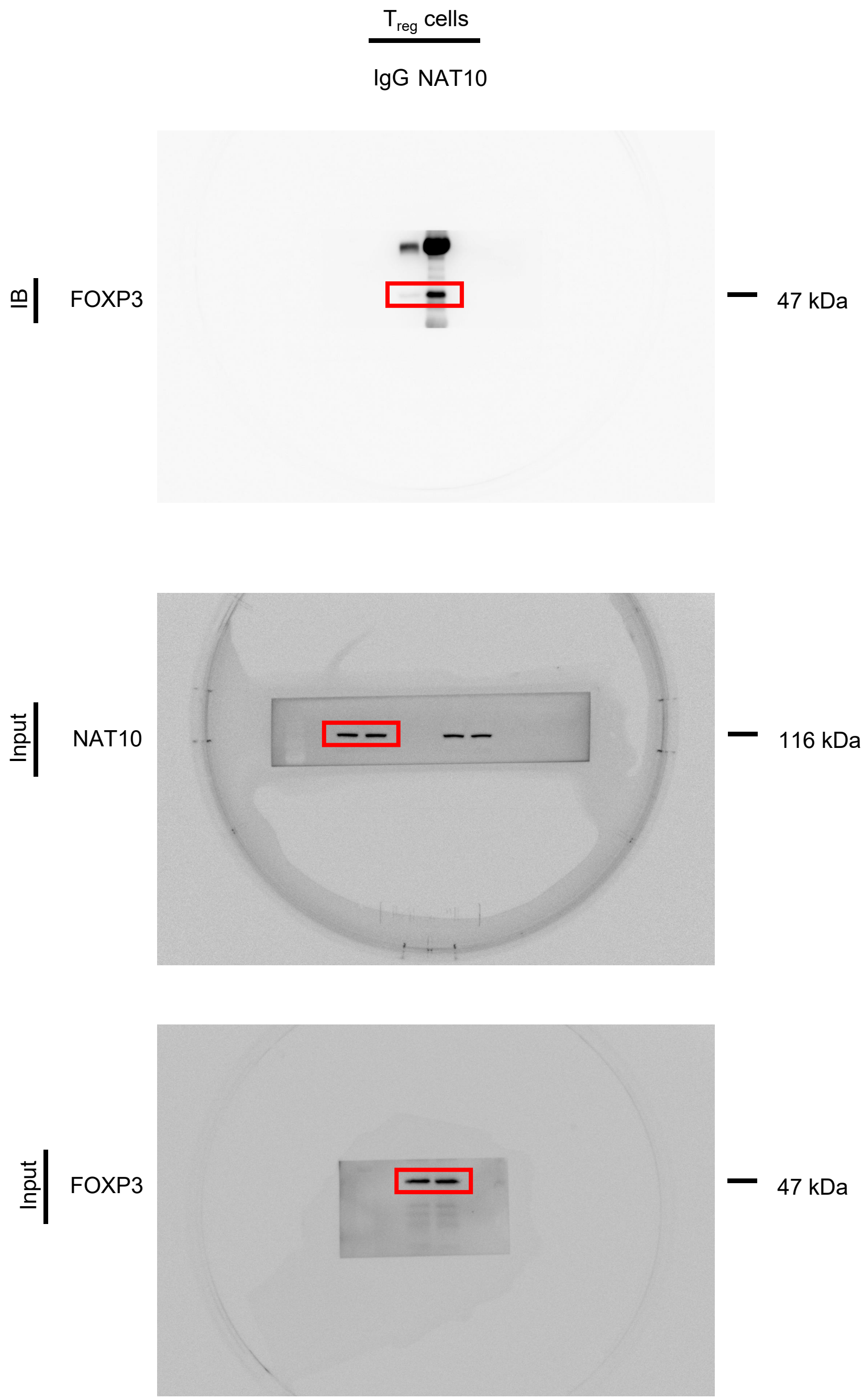

Full unedited blot for Figure 6B

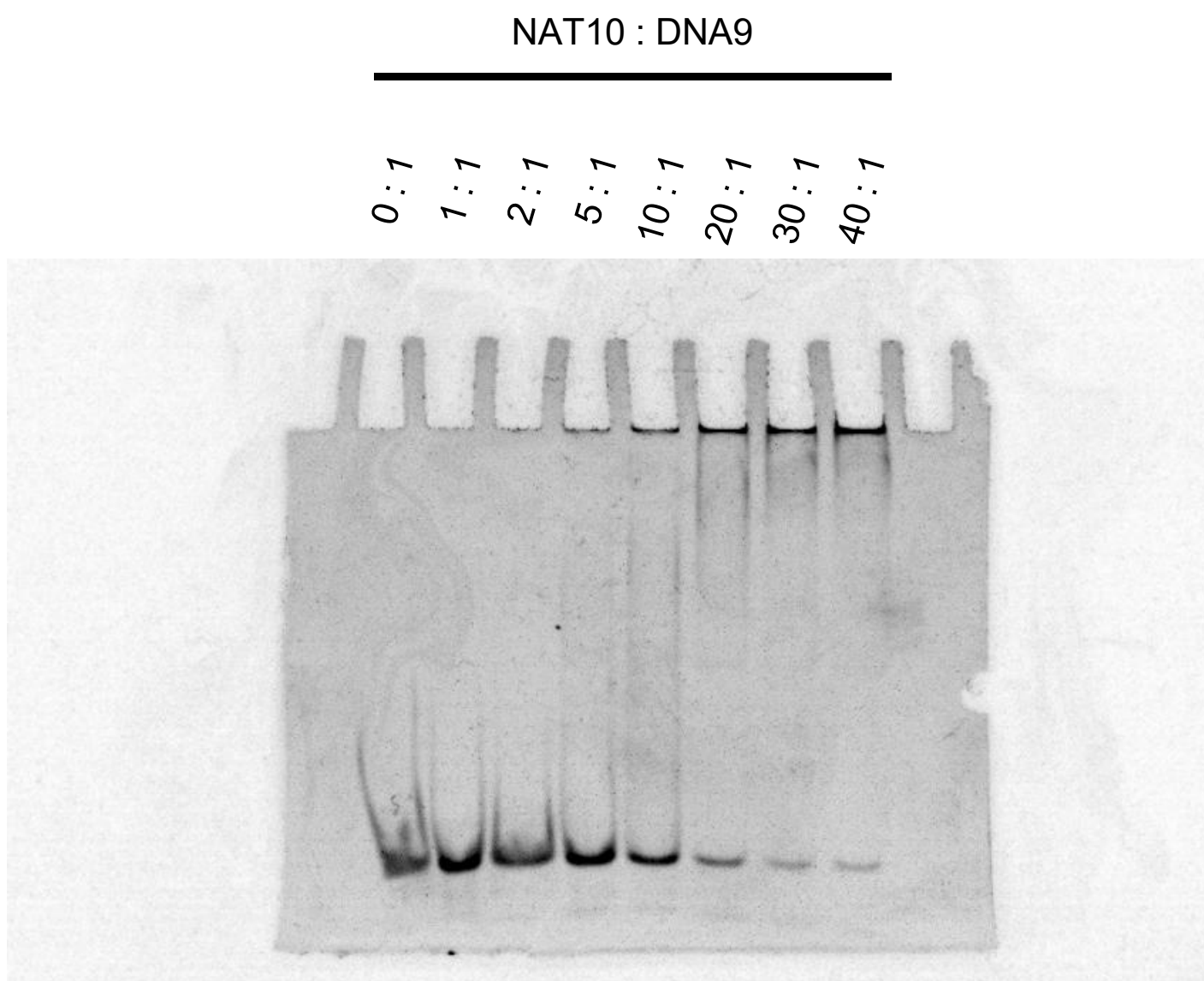

Full unedited blot for Figure 6D

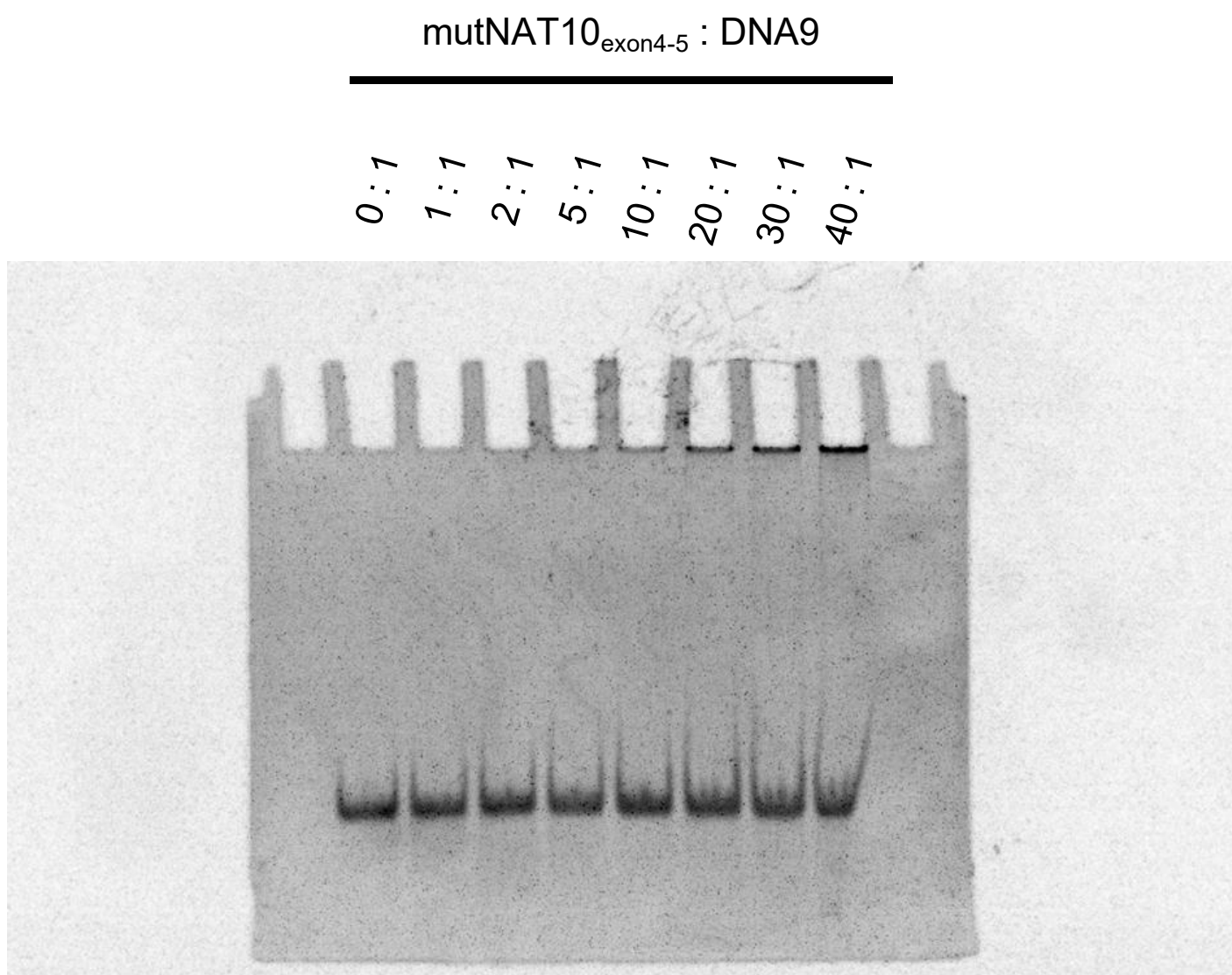

Full unedited blot for Figure S12A

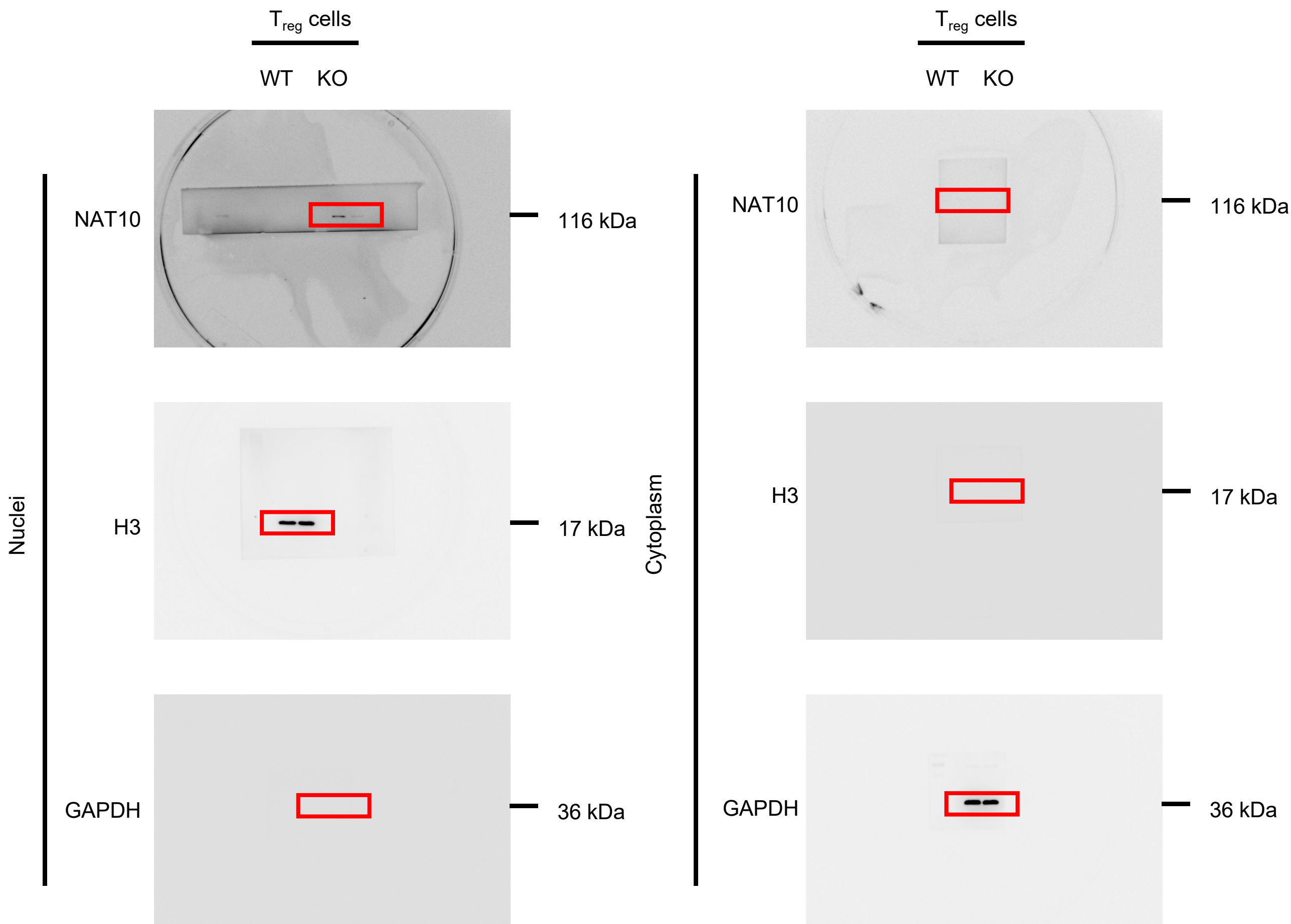

Full unedited blot for Figure S15B

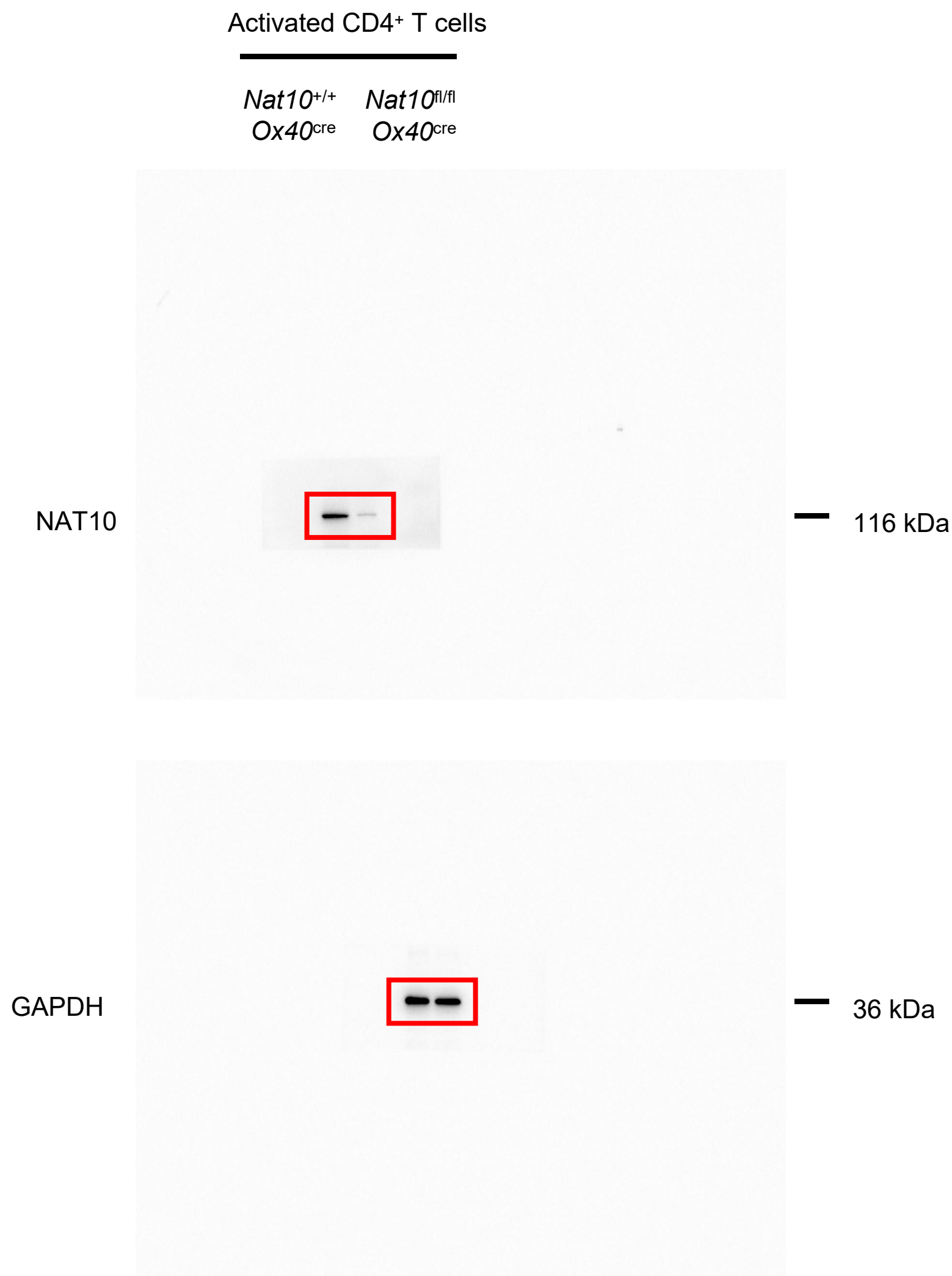

Full unedited blot for Figure S16A

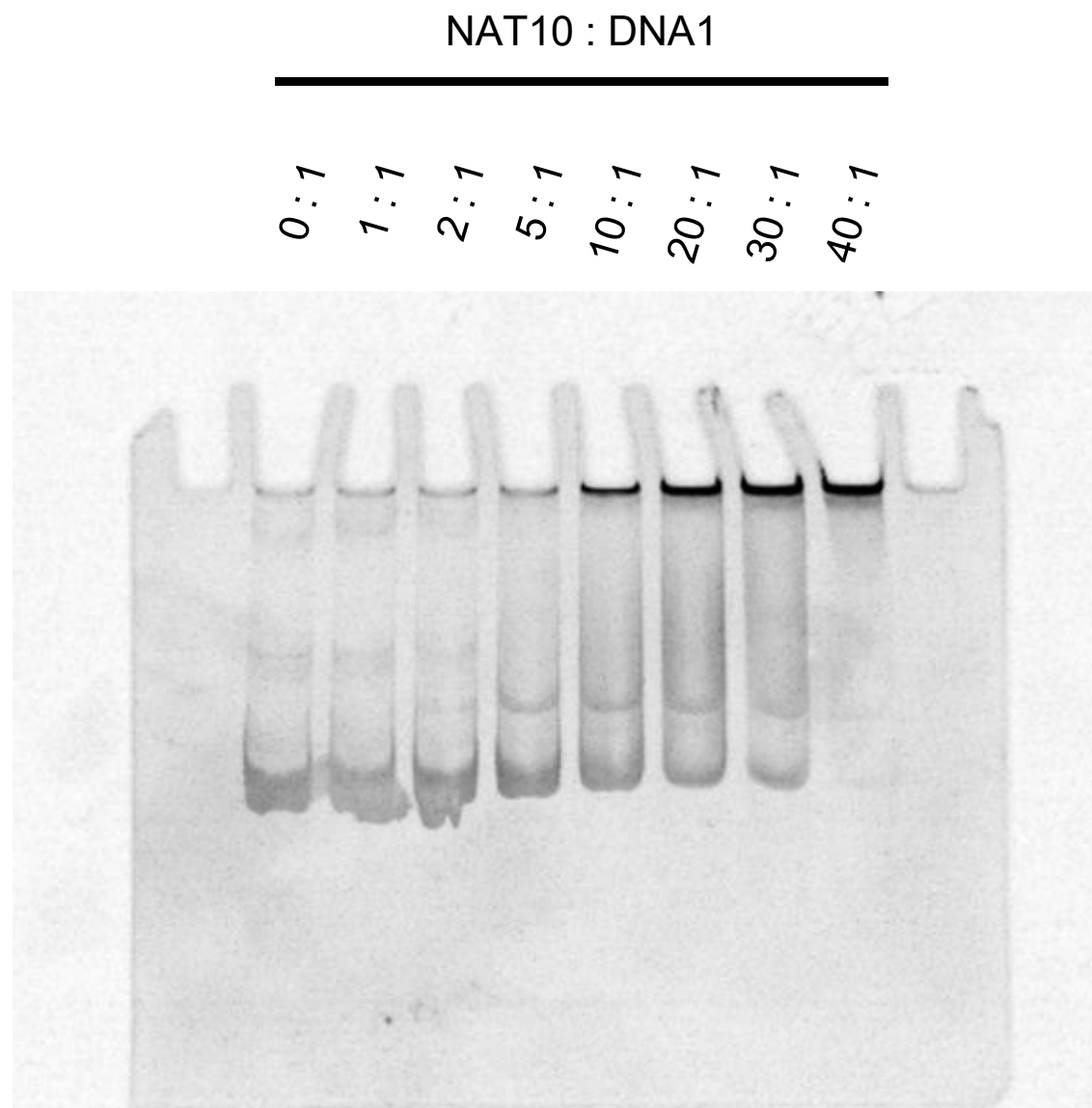

Full unedited blot for Figure S16B

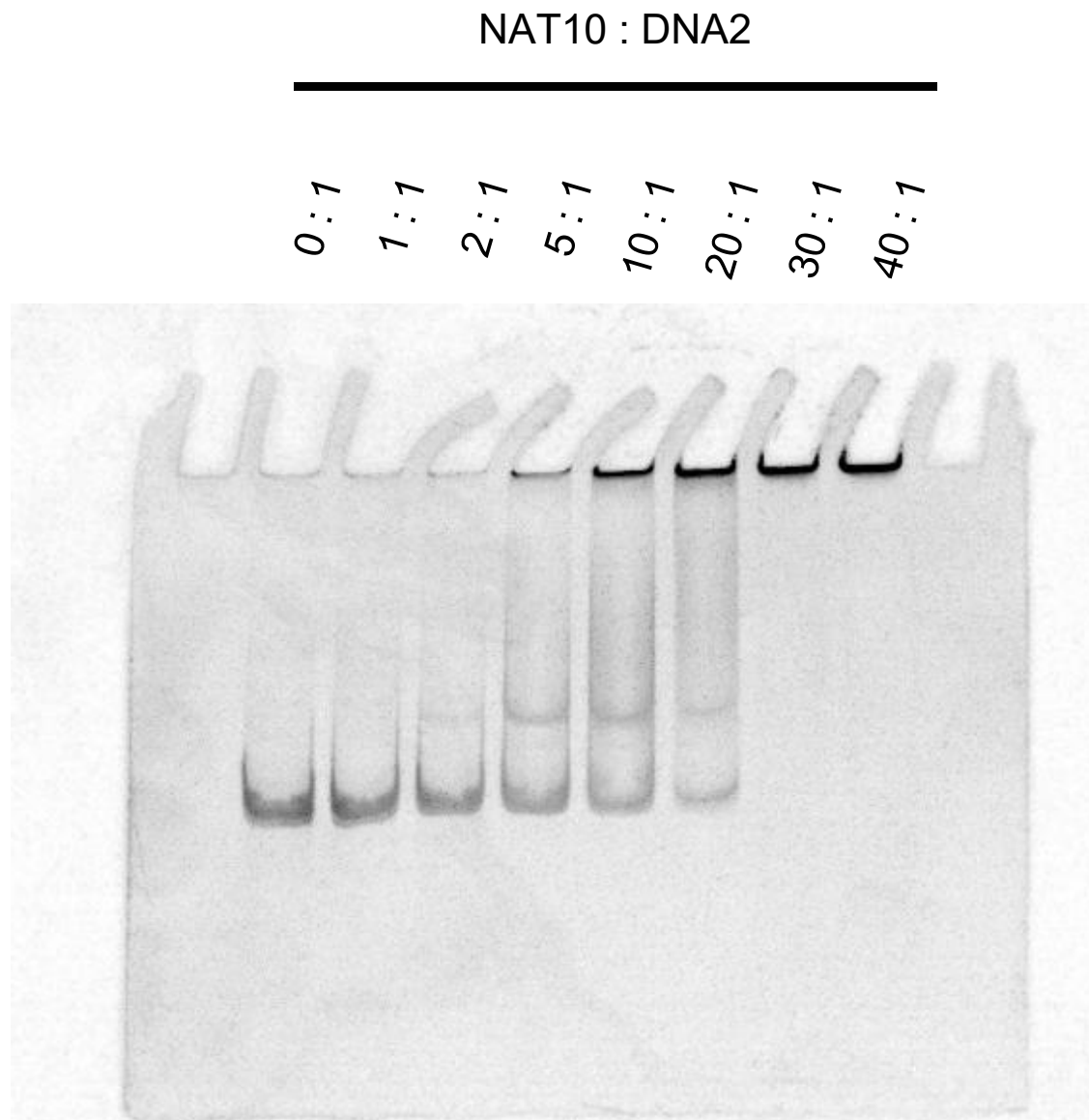

Full unedited blot for Figure S16C

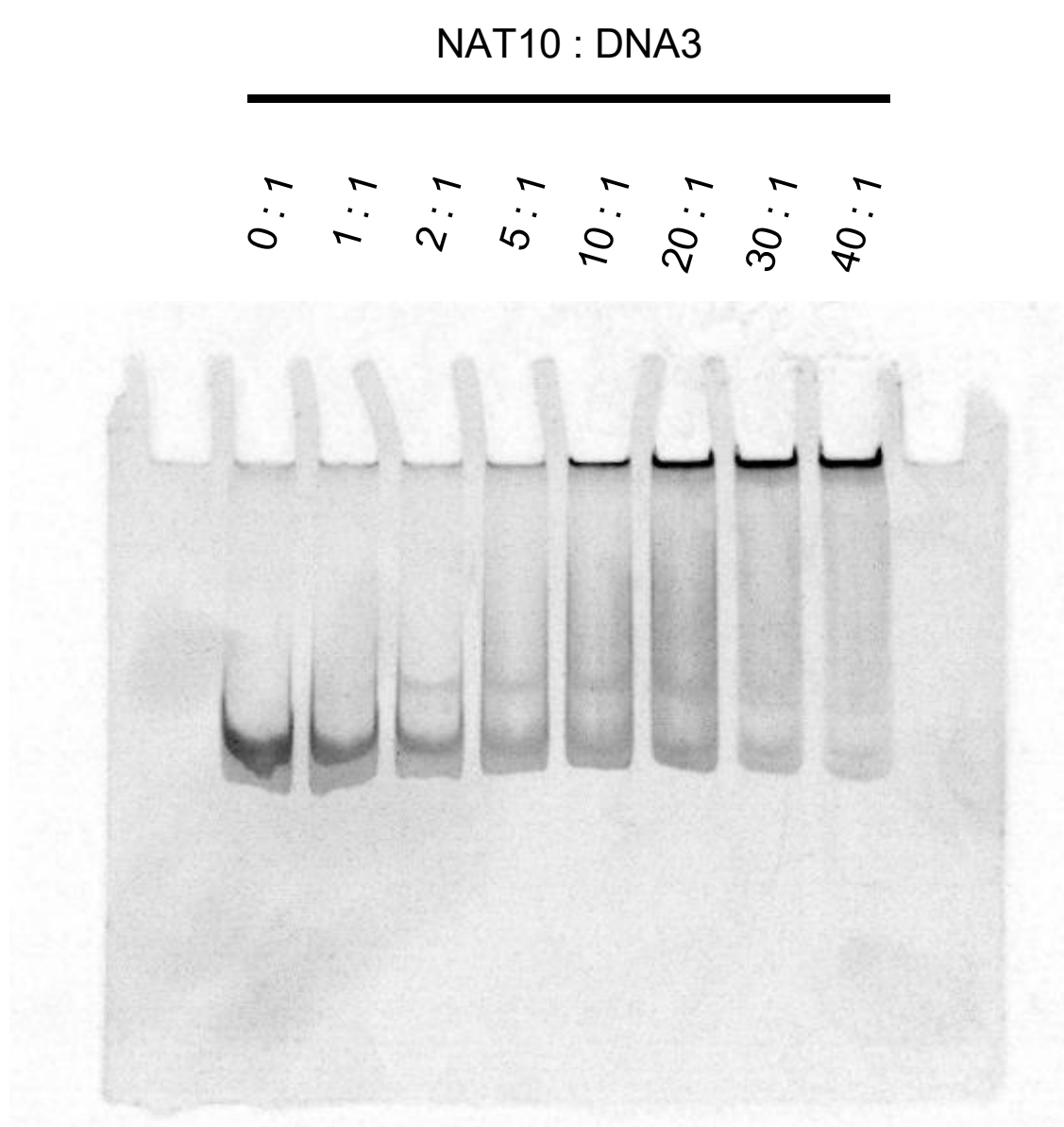

Full unedited blot for Figure S16D

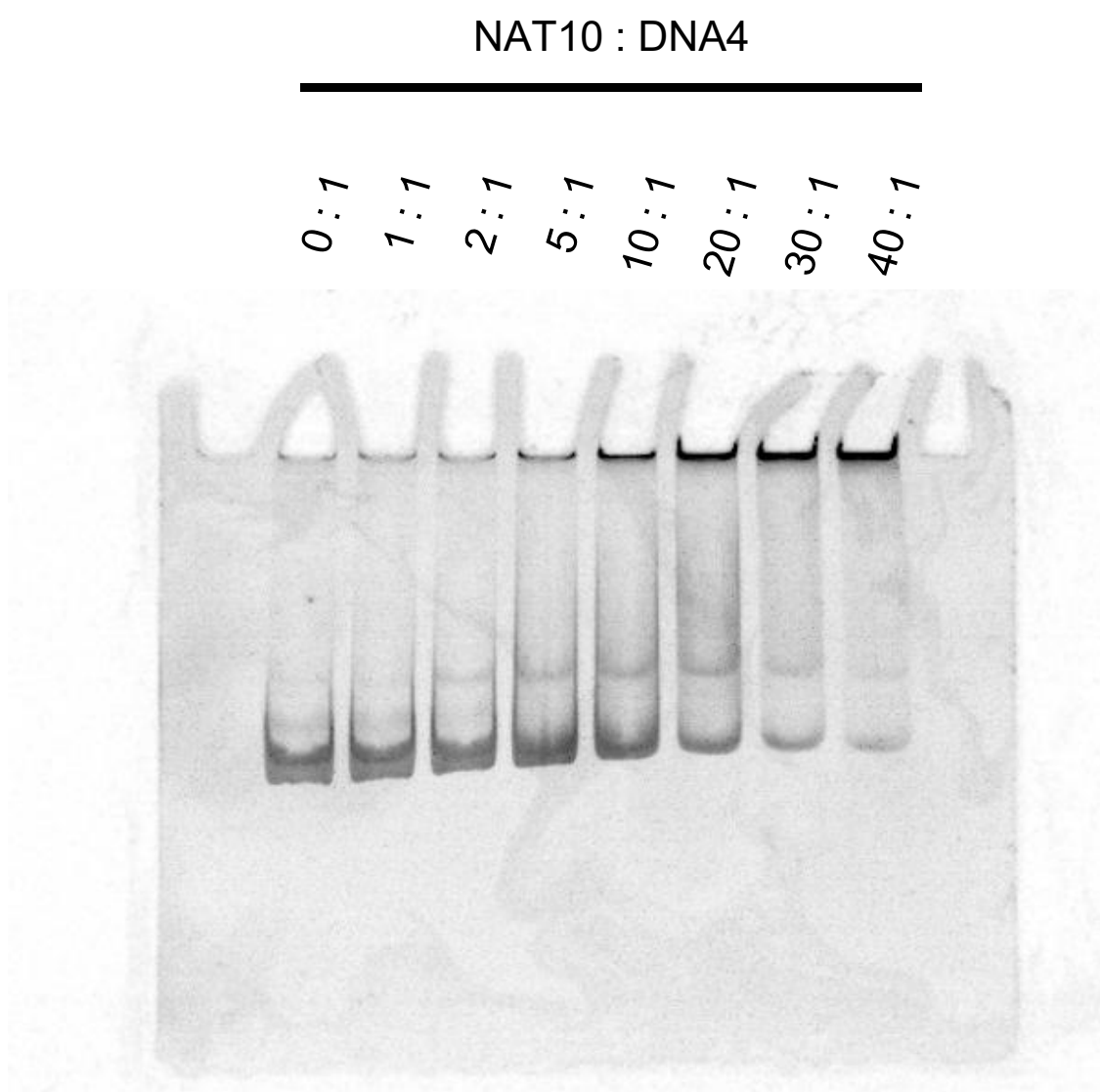

Full unedited blot for Figure S16E

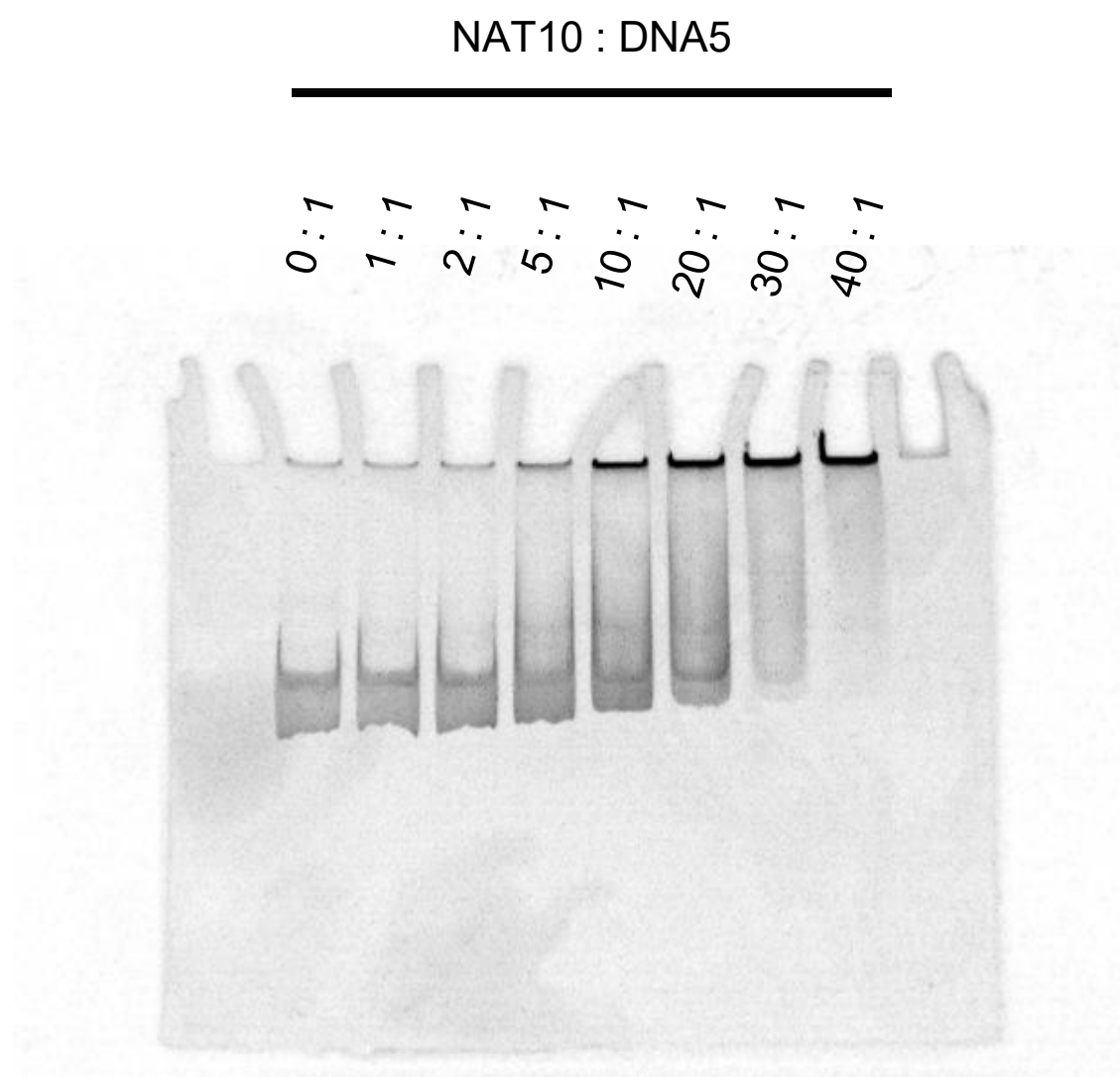

Full unedited blot for Figure S16F

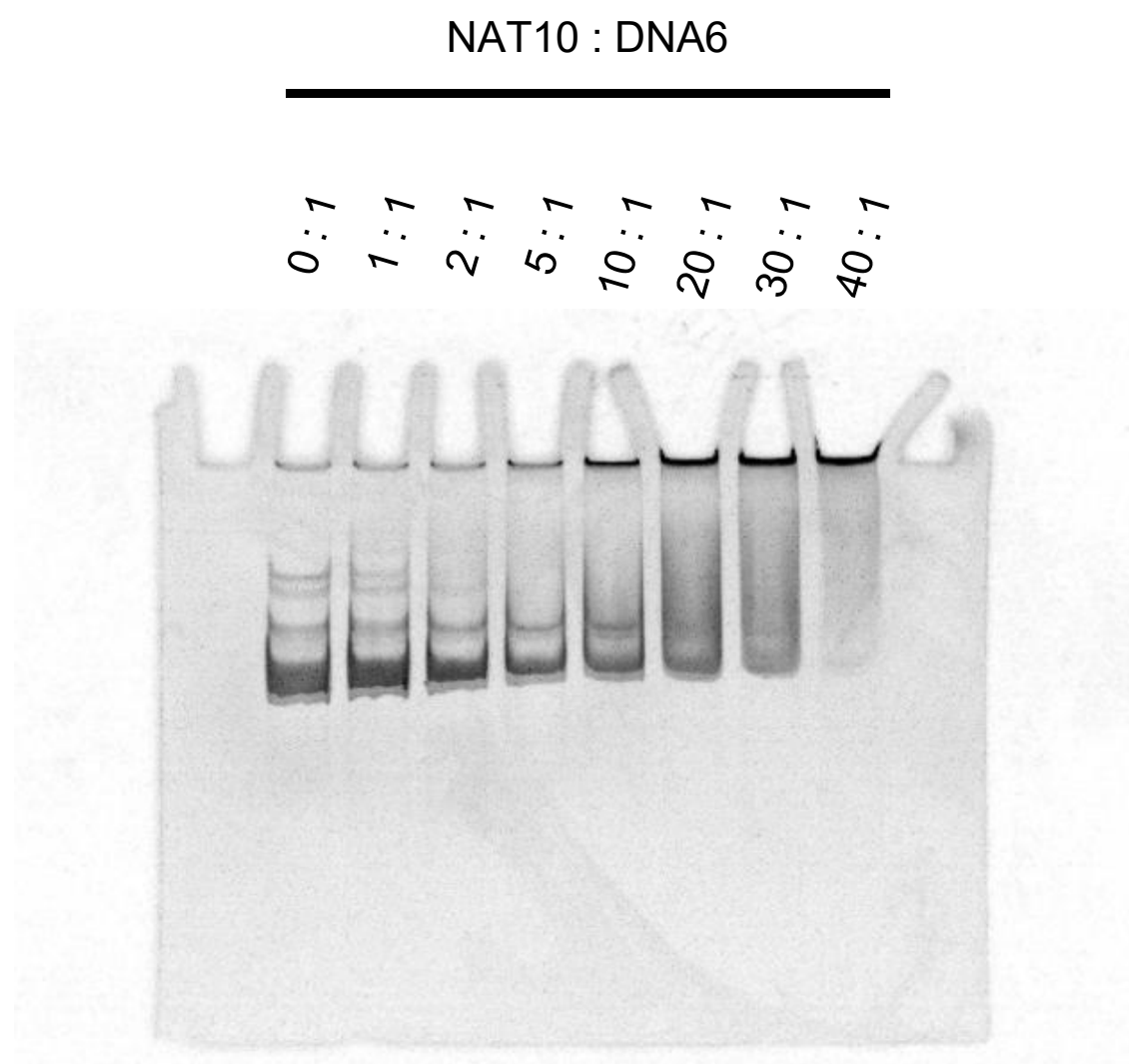

Full unedited blot for Figure S16G

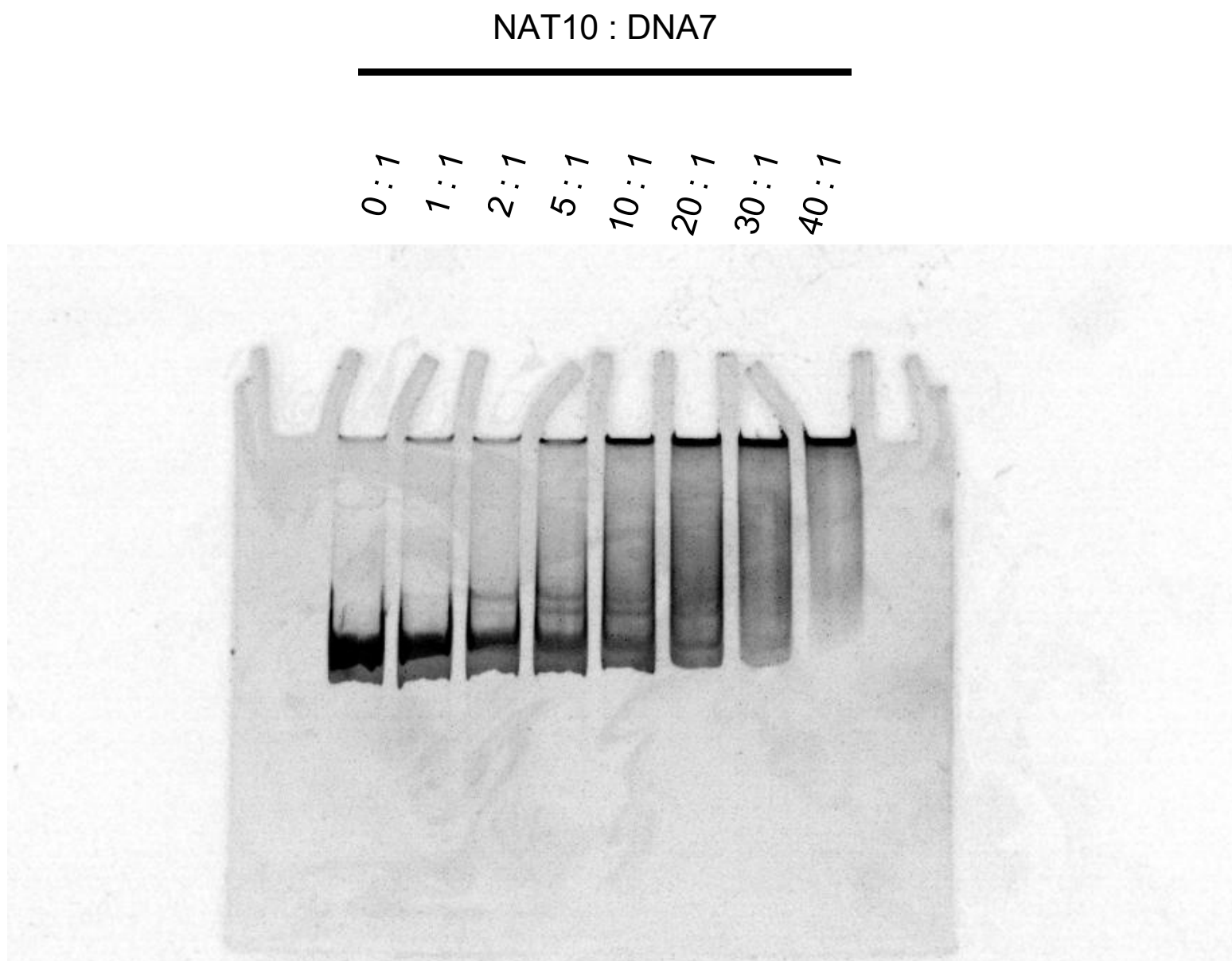

Full unedited blot for Figure S16H

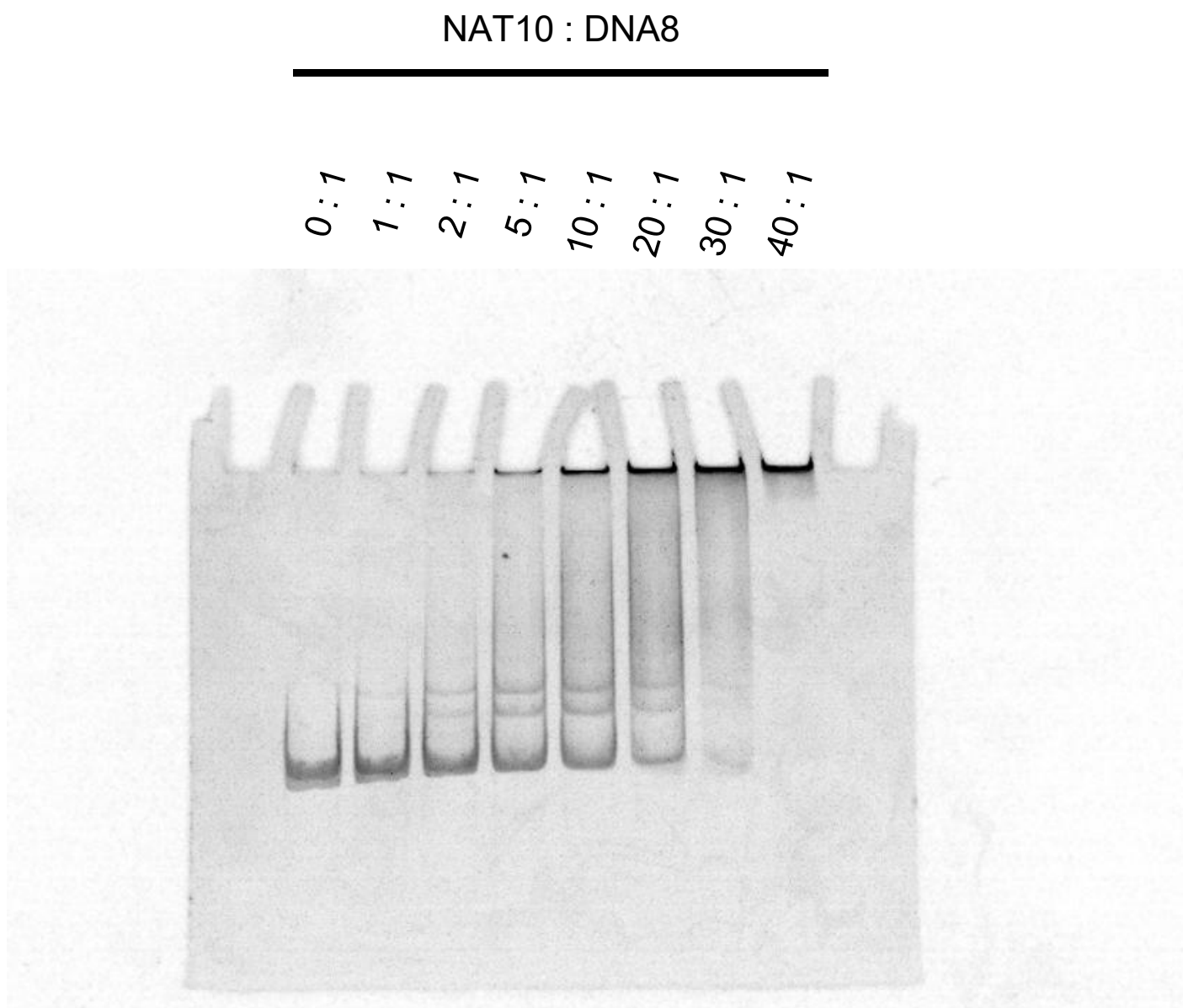

Full unedited blot for Figure S17A

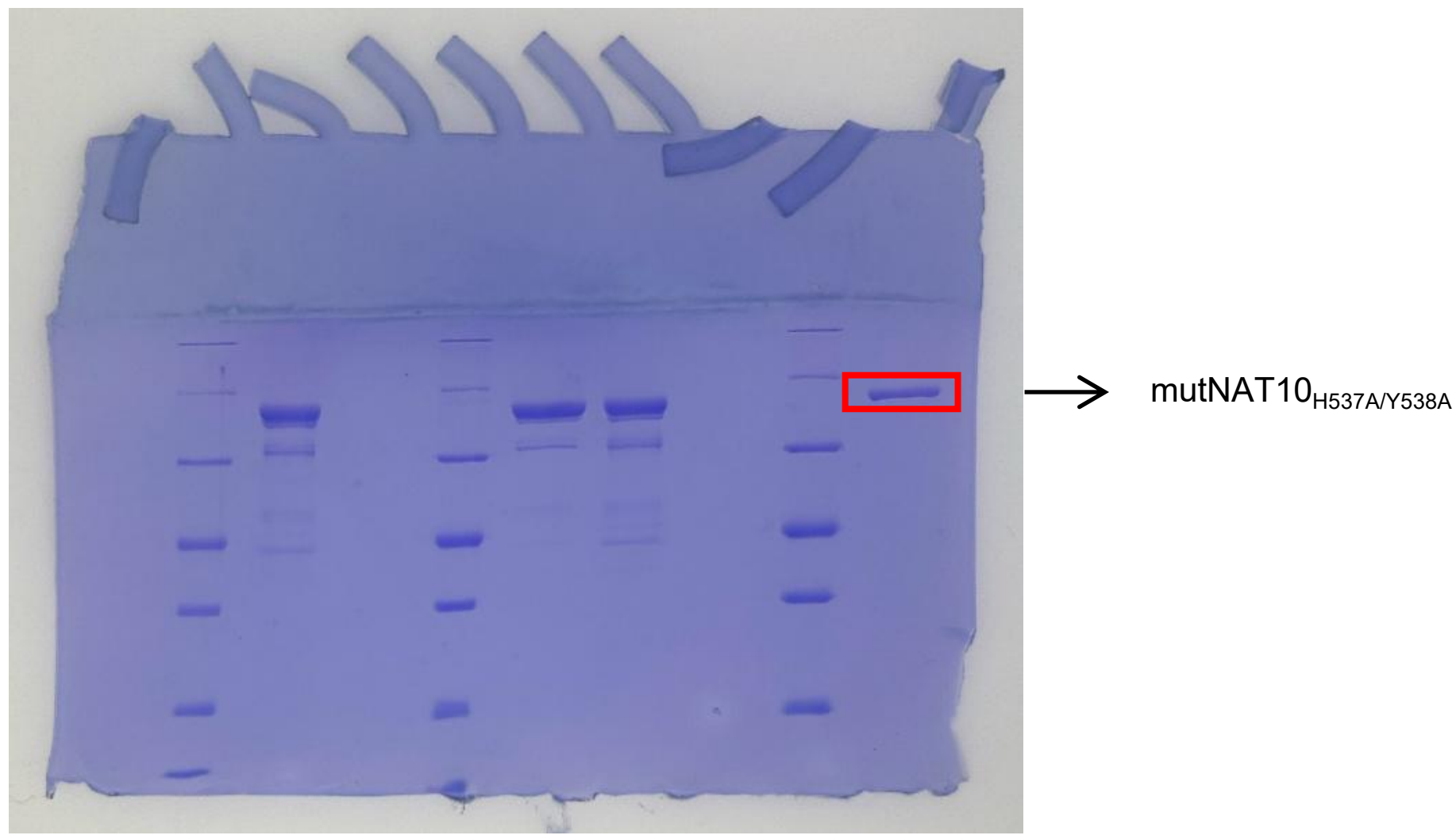

Full unedited blot for Figure S17C

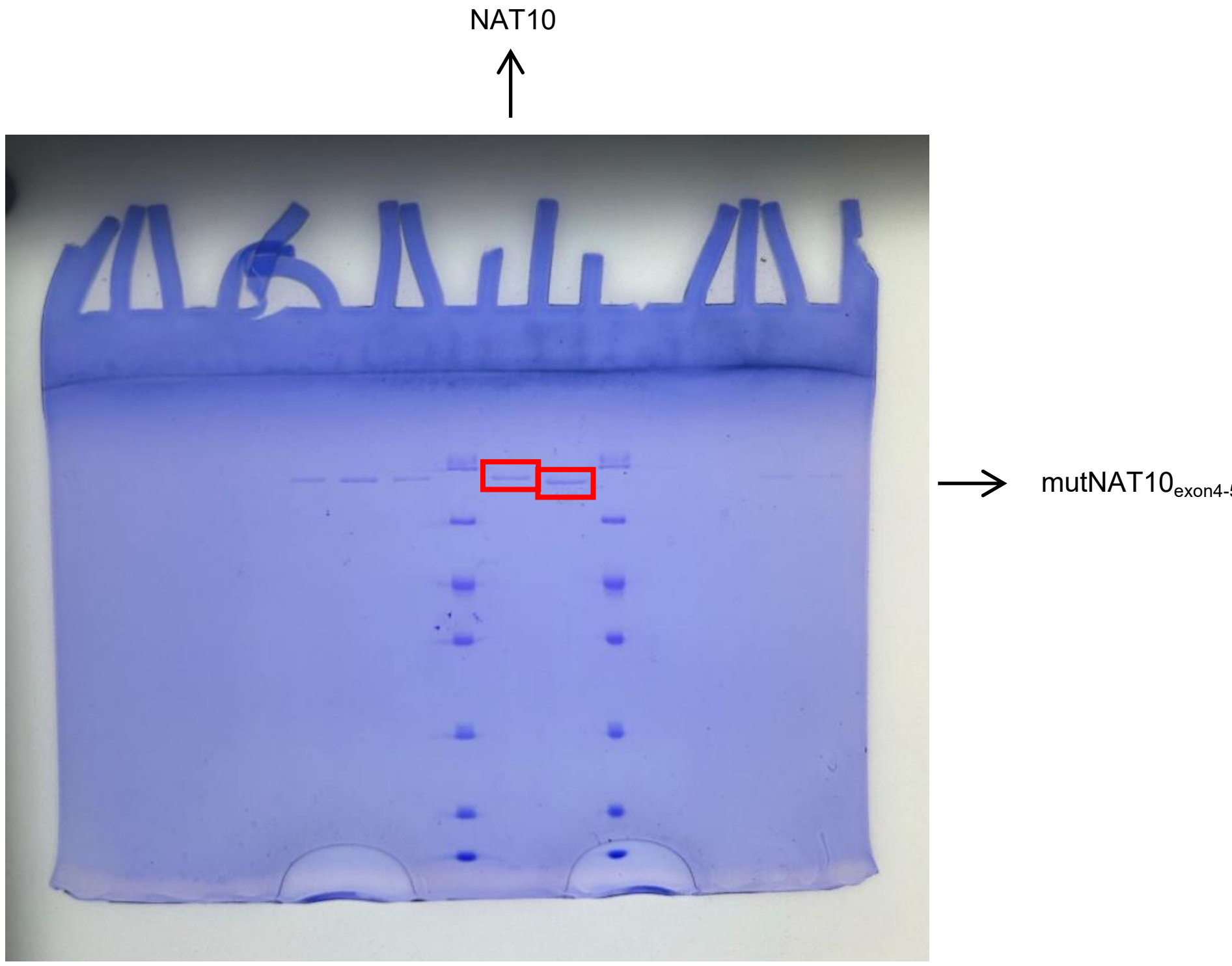

Full unedited blot for Figure S18E

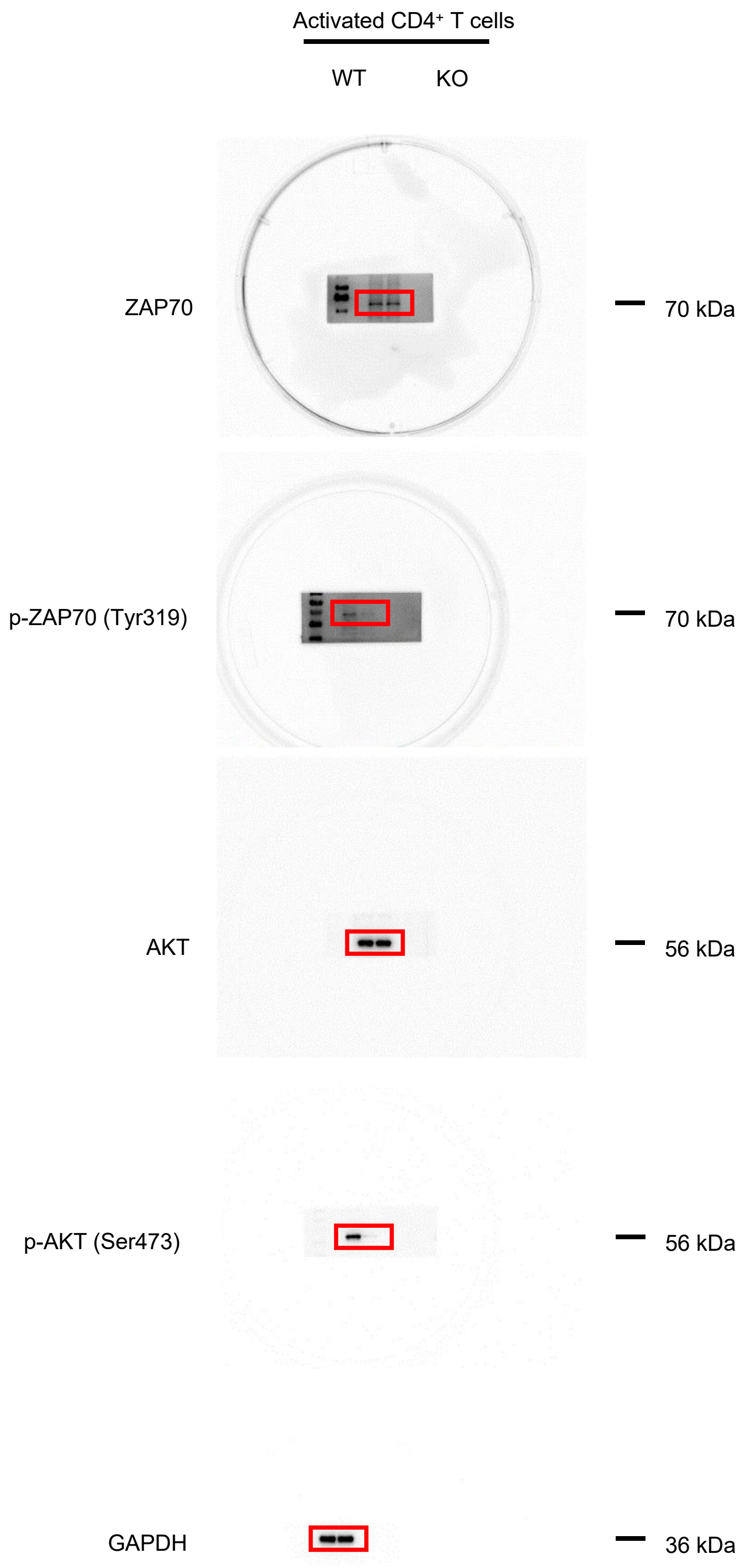

Full unedited blot for Figure S19C

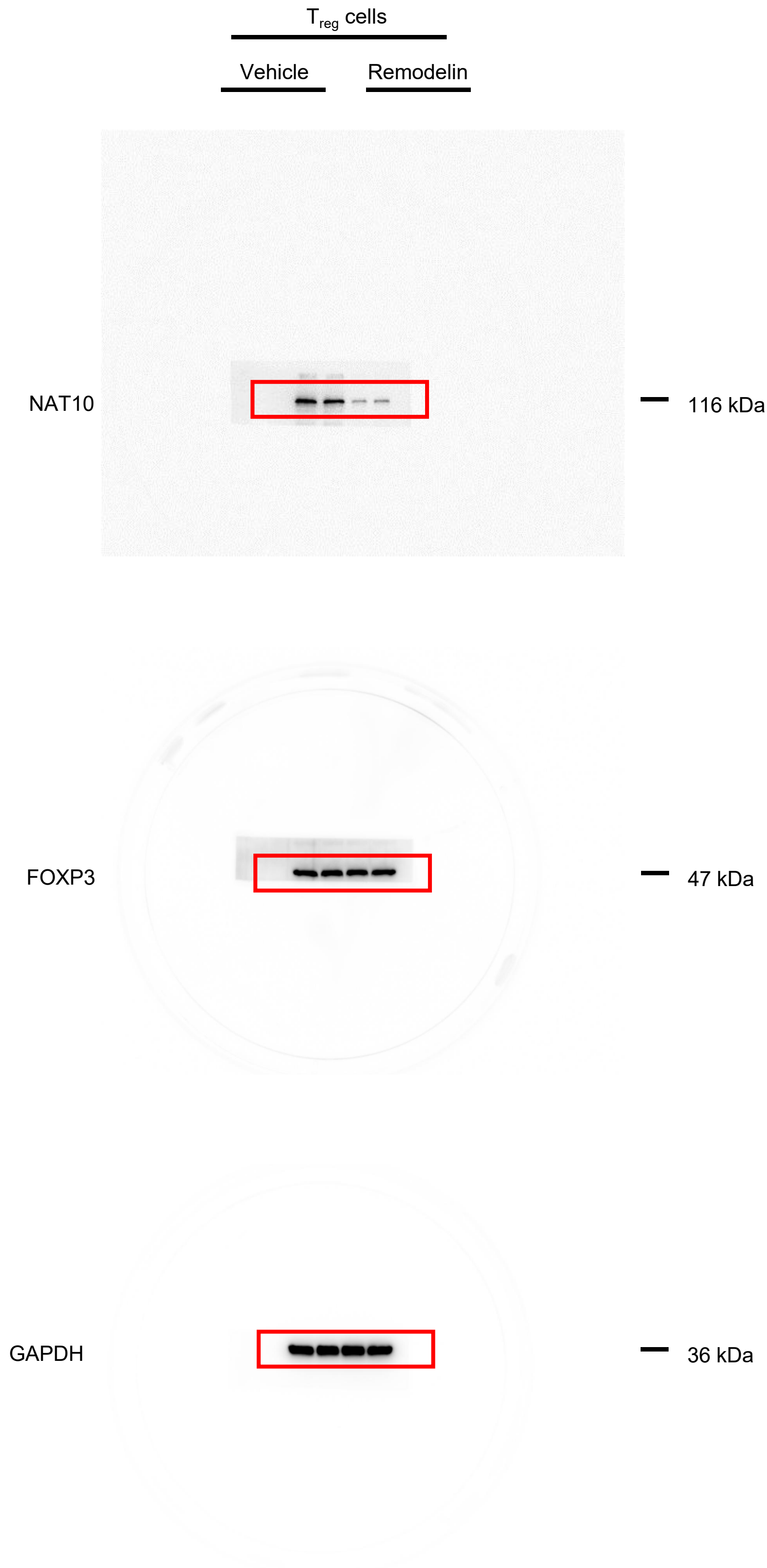

Full unedited blot for Figure S20B

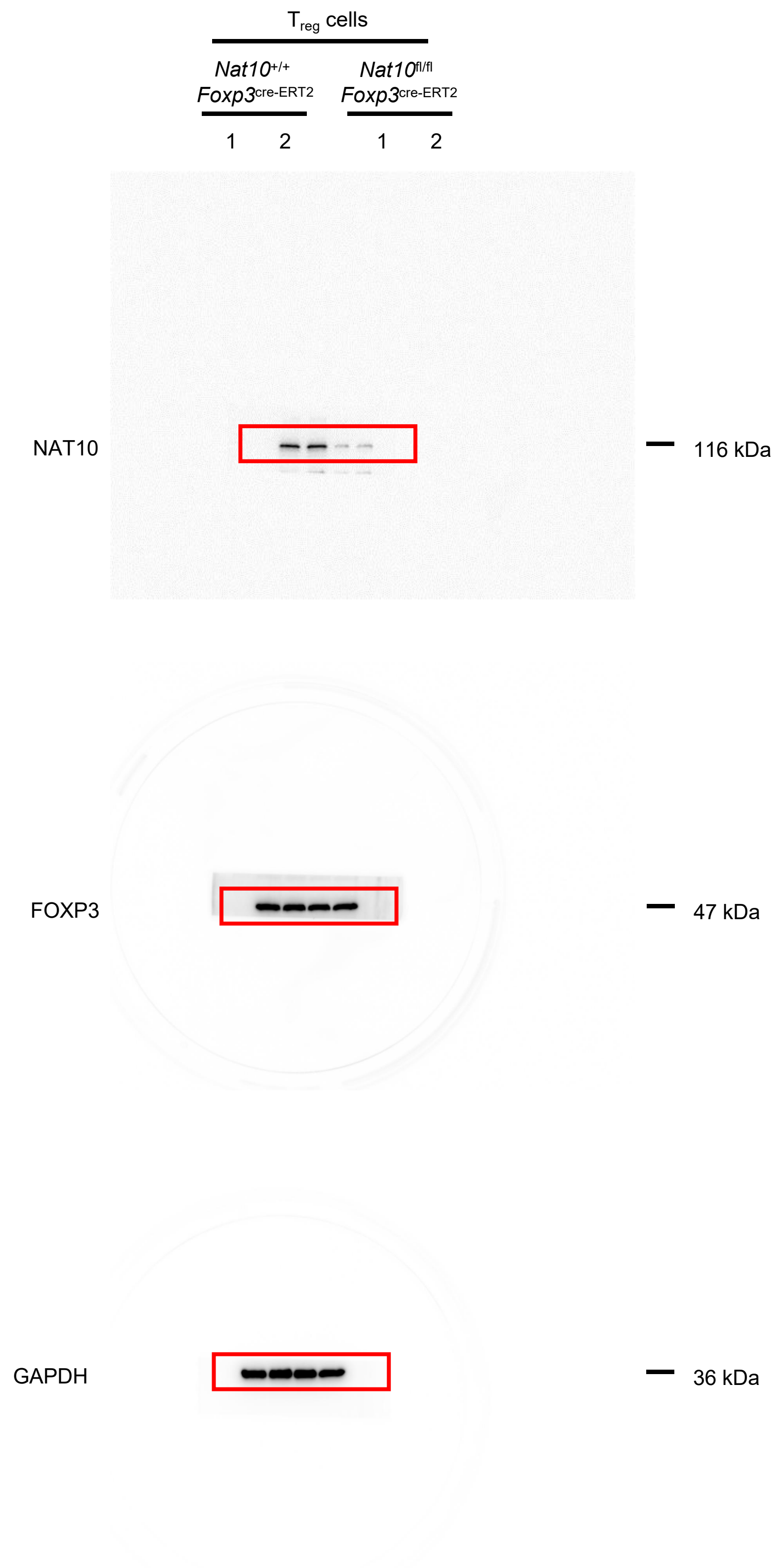

Full unedited blot for Figure S24B

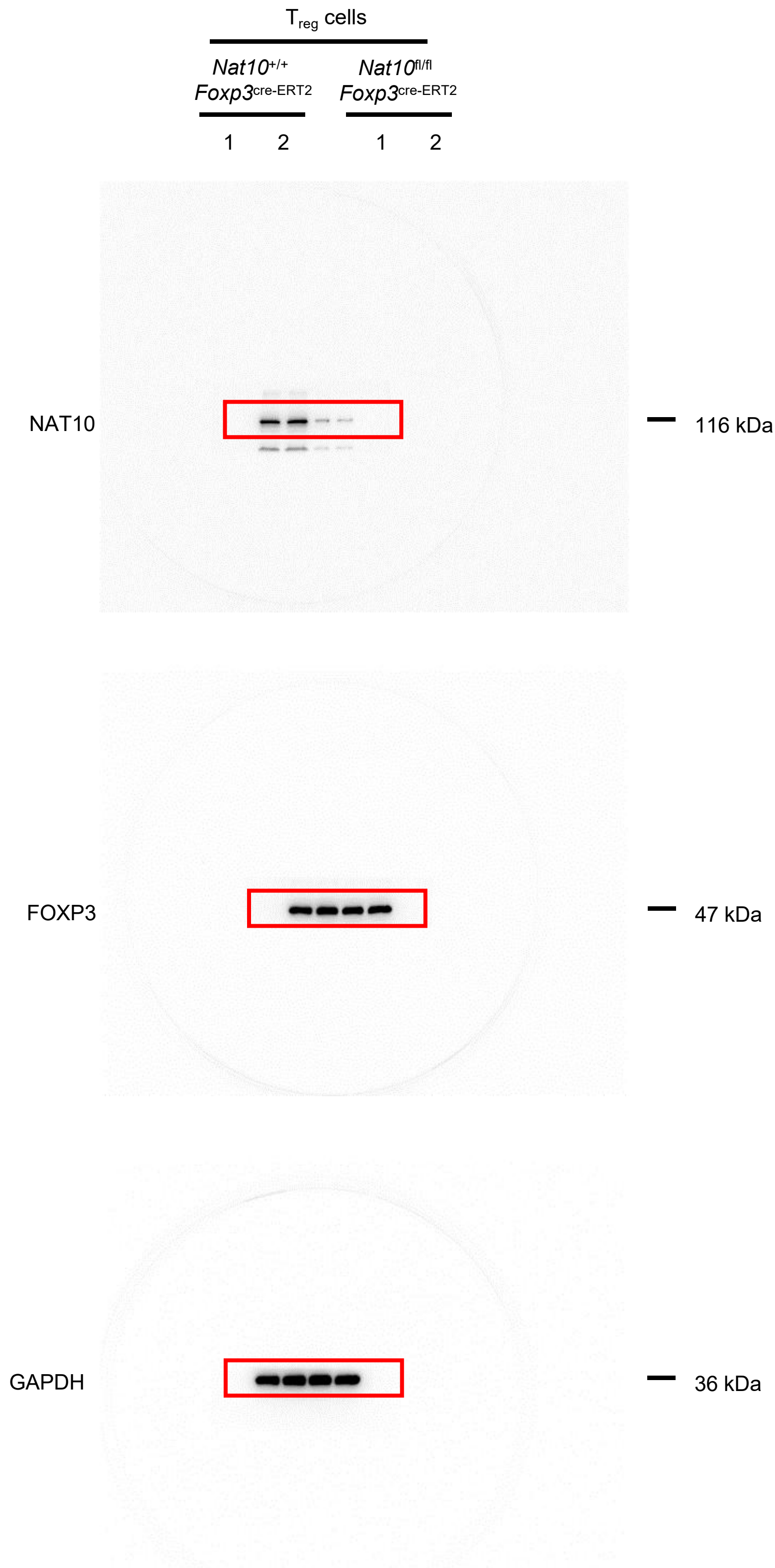

Supplement: Supplementary file 2 [file jci-136-189034-s241.pdf]
